# Phytoremediation performance of floating treatment wetlands with pelletized mine water sludge for synthetic greywater treatment

**DOI:** 10.1007/s40201-019-00372-z

**Published:** 2019-04-18

**Authors:** Suhail N. Abed, Suhad A. Almuktar, Miklas Scholz

**Affiliations:** 1 Civil Engineering Research Group, School of Computing, Science and Engineering, The University of Salford, Newton Building, Salford, England M5 4WT UK; 2 Department of Architectural Engineering, Faculty of Engineering, The University of Basrah, Al-Basrah, Iraq; 3 Division of Water Resources Engineering, Department of Building and Environmental Technology, Faculty of Engineering, Lund University, P.O. Box 118, 221 00 Lund, Sweden; 4 Department of Civil Engineering Science, School of Civil Engineering and the Built Environment, University of Johannesburg, Kingsway Campus, PO Box 524, Aukland Park, Johannesburg 2006 South Africa

**Keywords:** Eco–technology, Ferric oxide, *Phragmites australis*, Phytoremediation, Nutrient, Trace element

## Abstract

**Purpose:**

Buckets containing floating reed (*Phragmites australis*) simulated floating treatment wetlands (FTWs) and were used to improve the remediation performance of synthetic greywater (SGW). The aim of the study was to investigate the behaviour of FTWs for treatment of key contaminants within artificial greywater.

**Methods:**

Pelletized ochre based on acid mine water sludge was introduced to selected FTWs, because of its capability in sequestration phosphorus and other trace elements. The impact of the following four operational variables were tested in the experimental set–ups of the FTWs (four replicates each): pollutant strength (high– (HC) and low– (LC) concentrations), treatment time (2– or 7–days of hydraulic retention time (HRT)), presence or absence of macrophytes (*P. australis*) and cement–ochre pellets.

**Results:**

The results showed that 5 − day biochemical oxygen demand (BOD) and chemical oxygen demands (COD) were significantly (*p* < 0.05) reduced in all wetlands. Nitrate–nitrogen (NO_3_–N) concentrations were significantly (*p* < 0.05) higher, and those measurements for PO_4_–P were significantly (p < 0.05) lower than the corresponding ones determined for the influent. The existence of ochre pellets with *P. australis* significantly (*p* < 0.05) decreased B, Cd, Cr, Cu, Mg, Ni and Zn concentrations, but increased Al, Ca, Fe and K concentrations in the effluent, with the exception of sodium (Na).

**Conclusions:**

The FTW performances can be improved by utilising ochre–cement pellets to increase the pH of greywater. The presence of *P. australis* acts as a buffer to neutralise the pH of SGW. Rhizomes and biofilms mitigate increases in turbidity, TSS and colour values.

**Electronic supplementary material:**

The online version of this article (10.1007/s40201-019-00372-z) contains supplementary material, which is available to authorized users.

## Background

Urbanisation and industrial development coupled with increasing world population have led to intensive demands on water resources. Consequently, increased volumes of wastewater have provided the motivation to explore economical methods of collection, treatment and disposal, benefiting public health and the environment [1].

Household wastewater includes two types of discharge; black and grey wastewaters. Blackwater is defined as the derived effluent from toilets, bidets and urinals. Greywater refers to untreated domestic sewage generated from bathrooms, washing basins, laundry equipment, dishwashers and kitchen sinks, workplace buildings, schools, etc. [2–4]. Occasionally, effluents generated from kitchen sinks and dishwashers are excluded from greywater [5, 6]. Reuse of domestic wastewater became common, for non–drinking purposes [7]. Since greywater is defined as all domestic wastewater without toilet discharge, it represents the major fraction of the total household wastewater, which has a low level of pathogens and organic matter [4]. The most common practice of greywater reuse is for agricultural irrigation and toilet flushing [8], reducing the overall domestic water consumption [9]. However, recycling of wastewater for industrial, recreational, environmental and urban reuse options have become common practise [10]. The characterisation of grey wastewater based on an individual sample might be very misleading since the contaminant concentrations vary over the day and on different days of the week [4]. So, researchers have tried to address this challenge by mimicking greywater characteristics artificially [11]. Synthetic greywater constituents are either containing ingredients of domestic products and/or analytical grade chemicals to resemble the composition of various types of real greywater [12].

Pathogens and nutrients in greywater are significantly less common than in black wastewater. Moreover, there is commonly a high variation in trace element concentrations [4]. However, the organic and nutrient proportions in real greywater are usually too low for high-rate biological processes to take place effectively [6, 7, 13].

Among different treatment technologies, constructed wetlands have been a widely accepted option for treatment and recycling of greywater [7, 14] due to their ability to meet the requirements of public health, aesthetics, sustainability, technical design and affordability [8]. However, removal of phosphorus is only modest in constructed wetlands [15]. Significant land requirements make wetlands expensive in some locations [16]. To enhance phosphorus (P) removal, it is common practice to add aluminium or iron salts to a tertiary treatment process. This practise results in heavy floc blanket settling [17]. On the other hand, substances containing calcium such as lime are utilised to achieve good phosphorus removals served with a high level of pH, as this technique is more economic, especially for large volumes of discharge [18]. To reduce consumptions of commercial chemicals for phosphorus removal in wastewater treatment, it was suggested to use raw minerals and industrial by–products such as fly ash [19], slag [20], concrete waste [21], animal waste [22] and ochre sludge [23–25].

Phytoremediation is a vital natural process of pollutant removals from aquatic ecosystem. Saeed et al. [26] undertook an experiment where a constructed floating mat vegetated with *P. australis* (commonly available, cheap, deep–rooting and fast−growing) and *Canna indica* showed a decrease in nitrogen due to nitrification–denitrification processes, while the phosphorus removal was influenced by filtration and sedimentation actions. Protozoa predation and oxidation processes in this system affected positively the removal rates of *Escherichia coli*. This system showed greater removal of nutrients and *E. coli* during the dry period [26]. Furthermore, the efficacy of FTWs can be enhanced by inoculation with bacteria for wastewater applications of, for example, municipal [27] as well as domestic and industrial origins [28].

The common criteria to select wetland macrophytes depend on their availability and abundance in the corresponding region of study. Sooknah and Wilkie [29] selected three floating aquatic macrophytes, namely *Eichhornia crassipes*, *Hydrocotyle umbellata*, and *Pistia stratiotes* to evaluate the quality improvement of wastewater from anaerobically digested flushed dairy manure in terms of nutrients, COD, solids and salinity. Other free–floating aquatic macrophytes such as *Ipomoea aquatica*, *Paspalum repens*, *Azolla microphylla*, *Salvinia minima Baker* and *Lemna minor* have been examined for their effectiveness in treating organic matter and nutrients in wastewater from a dairy farm, a dairy processing plant, a banana paper plant and a landfill [30]. Floating treatment wetlands planted with emergent macrophytes such as *Carex acutiformis*, *Carex virgata*, *Cyperus ustulatus*, *Juncus edgariae* and *Schoenoplectus tabernaemontani* have been investigated for their ability to tolerate high fluctuations in water depths that are typical of storm water ponds [16, 31]. Borne et al. [32] reported a significantly improved runoff water quality for floating treatment wetlands vegetated with *Carex virgata* for remediation of suspended solids, particulate zinc as well as particulate and dissolved copper compared to parallel storm water treatment ponds monitored in a field trial study. In addition, the floating treatment wetland has shown an increase in adsorption processes for insoluble copper sulphides and direct copper uptake by plants at presence of high humic content, low dissolved oxygen and natural water pH. Ladislas et al. [33] assessed the adsorption mechanisms of *Juncus effusus* and *Carex riparia* roots for remediation of cadmium, nickel and zinc from urban water runoff, which demonstrated a high capacity to adsorb dissolved metals and filter particulates. An alternative method for the selection of vegetation species is to select according to their efficiency of nutrient removal [34–36].

Floating treatment wetlands are simple in design and require no additional land resulting in savings compared to conventional constructed wetlands [16]. The immersed rhizomes and roots of macrophytes provide a large surface area to develop a biofilm, which plays a key role in the removal of suspended contaminants from the water column [37–40]. Moreover, roots of some plants supply dissolved oxygen into the waterbody benefiting the growth of aerobic microorganisms, which break organic substances down [41, 42].

This study evaluated the purification of artificially prepared greywater by floating reed beds [43, 44] in combination with cement–ochre pellets for removal of phosphate and other trace elements. The corresponding objectives were: a) to assess the effluent quality as a function of hydraulic retention time (HRT), pollutant concentration strength, existence of *P. australis*, and contribution of cement–ochre solids in treatment processes; b) to evaluate and analyse cement–ochre pellets for accumulative trace elements during the treatment period; and c) to evaluate and analyse biomass tissues of *P. australis* for accumulative trace elements and their distribution in individual plant parts.

## Methods

### Artificial greywater

Domestic greywater was made synthetically by applying adopted analytical–grade substances [45], which were supplied by Fisher Scientific Co. Ltd. (Bishop Meadow Road, Loughborough, UK). Stock solutions were the basis for synthetic greywater (SGW) with low concentrations (LC) and high concentrations (HC). Chemicals subject to a recipe (Online Resource 1) were subsequently added to tap water and mixed by a magnetic stirrer for one hour at 1200 rpm. The stock solutions were thinned with drinking water at a ratio of 1 to 100, and stored at 4 °C, and mixed again for 30 min before being used in further experiments [45].

### Cement–ochre pellets

Ochre was obtained from the mine sludge handling and treatment works at the Deerplay coal mine (53°44′06”N 2°11′49”W), which is located just north of Rochdale (OL13 8RD), UK. Drying beds were used to remove surplus water, and the concentrated ochre was stockpiled on–site. The moisture content of the ochre was 87%. Portland cement (three parts) was added to the raw ochre sludge (seven parts) to produce pellets [24, 25, 46].

### Experimental set–up design

The intent of the tested microcosm FTW cell system was to mimic constructed retention systems with floating reed beds under authentic weather conditions on top of a building at The University of Salford (53°29′09.3“N and 2°16’24.8”W). The test was performed between 1st September 2014 and 1st November 2016. The assessment commenced on 1st November 2014. The biofilm growth was unhindered for two months; September and October 2014. About 14–litre plastic buckets (purchased from B&Q, Manchester, UK) were filled with 10 l of SGW.

Washed bare–rooted *Phragmites australis* (*Cav.*) *Trin. ex Steud.* (common reed) plants were supplied by VESI Environmental Ltd. (Little Island, Co. Cork, Ireland). The corresponding roots and rhizomes (about 1 l) were submerged to a depth of about 30 cm.

The authors assessed the ability of microcosm FTW cell systems, when cement–ochre pellets and *P. australis* were present, to treat greywater. The ability of macrophytes to treat synthetic greywater (SGW) at two different pollutant loadings subject to short (2 days) and long (7 days) HRTs was assessed. The selected retention times are considered typical of floating treatment wetlands [35, 46, 47].

**Fig. 1:**
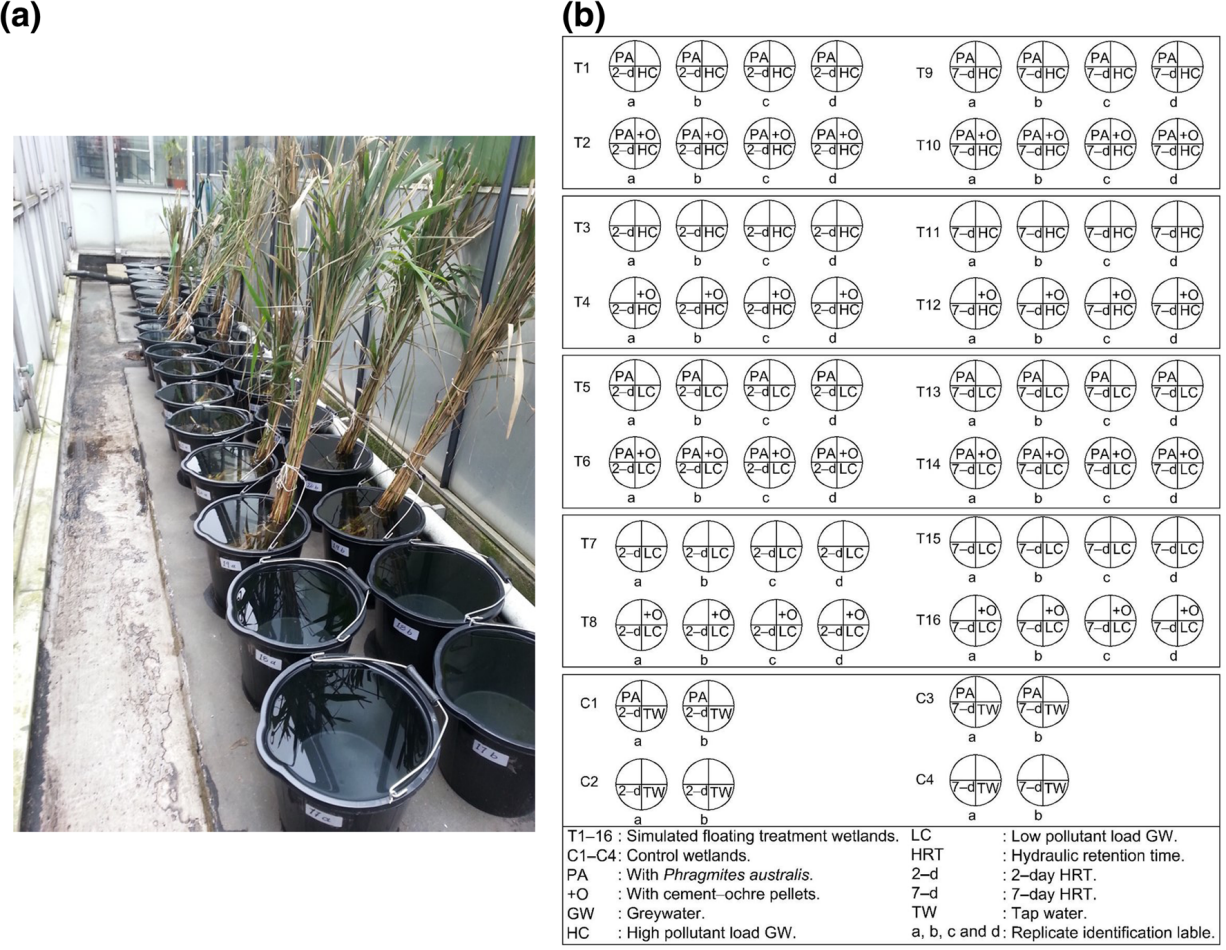
**a** photo of part of the experimental set-up at the start of the experiment; and (**b**) sketch illustrating the operational variables in the experimental set–up design of the simulated floating treatment wetlands

The set−up design shows 72 FTW microcosms (Fig. 1b). The first group was characterised by 2 days of HRT for eight sets of microcosms (T1, T2, T3, T4, T5, T6, T7 and T8). The second group of microcosms (T9, T10, T11, T12, T13, T14, T15 and T16) was subjected to 7 days of HRT. Each set had four replicates marked by the microcosm number directly followed by a letter (either a, b, c or d) to identify replicates. Ten litres of synthetic greywater with high contamination contents (HC–SGW) were treated in microcosms T1, T2, T3 and T4 for a HRT of 2 days and in microcosms T9, T10, T11, and T12 for 7–day HRT. Greywater with low contamination content (LC–SGW) was treated in microcosms T5, T6, T7 and T8 for 2–day HRT, and in microosms T13, T14, T15 and T16 for 7–day HRT. Microcosms T1, T2, T5, T6, T9, T10, T13 and T14 were vegetated with *P. australis*. Cement–ochre pellets (300 g) were designed to treat 10 l of SGW each in microcosms T2, T4, T6, T8, T10, T12, T14 and T16 (Fig. 1). Therefore, floating *P. australis* combined with cement–ochre pellets were applied in FTW microcosms T2, T6, T10 and T14 as part of the experimental investigation, while microcosms T3, T7, T11 and T15 were left with only synthetic greywater (i.e. without vegetation and without cement–ochre pellets). The experimental set–up design included four sets of microcosms with two replicates that served as controls (C), which were fed by ten litres of de–chlorinated tap water. All control sets were labelled with the capital letter C followed by the number of the corresponding microcosm, which was either 1, 2, 3 or 4, and a small letter (a or b) allowing for the identification of replicates. The vegetated control microcosm C1 and the non–vegetated control microcosm C2 were linked to 2 days of HRT, while vegetated C3 and non–vegetated C4 benefitted from 7 days of HRT. The simulated greywater in the FTW microcosms (T) was exchanged completely (without disturbing the biofilm) with fresh SGW after the specific time of retention.

### Water quality assessment

Water quality examinations were performed according to APHA [48]. The spectrophotometer DR 2800 Hach Lange (www.hach.com) was used for the assessment of parameters such as chemical oxygen demand (COD), ammonia–nitrogen (NH_4_–N), nitrate–nitrogen (NO_3_–N), ortho–phosphate–phosphorus (PO_4_–P), total suspended solids (TSS) and colour. The 5–day biochemical oxygen demand (BOD) was determined with the OxiTop IS 12–6 system according to Wissenschaftlich–Technische Werkstätten (WTW), Weilheim, Germany. Turbidity was determined with a Turbicheck Turbidity Meter (Lovibond Water Testing, Tintometer Group). Electric conductivity (EC) was measured by the conductivity meter METTLER TOLEDO FIVE GOTM (Keison Products, Chelmsford, Essex, England, UK). Hydrogen ion (pH) and redox potential (Eh) were determined with a sensION+ benchtop multi–parameter meter (Hach Lange, Düsseldorf, Germany). Dissolved oxygen (DO) was recorded by a HQ30d Flexi Meter (Hach Lange, Düsseldorf, Germany).

### Trace element analysis

Minerals and trace elements of inflow and outflow greywater were analysed following the SW–846 Test Method 6010D [49] by applying Inductively Coupled Plasma–Optical Emission Spectrometry (ICP–OES) using Varian 720–ES (Agilent Technologies UK Ltd., Wharfedale Road, Wokingham, Berkshire, UK).

The USEPA Method 200.7 was used to analyse the raw ochre sludge and the cement–ochre pellets [50]. Trace elements were also analysed for *P. australis* tissues such as roots, rhizomes, stems and leaves. Solid samples were acid−digested following the USEPA Method 3050B [51].

### Statistical evaluation

Microsoft Excel and the Statistical Package for the Social Sciences (IBM–SPSS) Statistics Version 23 (www.ibm.com) were used to analyse data at a 95% confidence level. The independent sample T–test was used to evaluate sample averages from two independent groups, if their distribution was found to be normal using the normality test of Shapiro–Wilks. The non–parametric test (Mann–Whitney test) was used to match two independent samples when their distributions were not normal. Furthermore, the non–parametric Kruskal–Wallis technique was applied for data that were non–normal distributed. An assessment of the consistency of variances was performed by applying Levene’s test for both parametric and non–parametric techniques. Correlations were assessed with Spearman’s test.

## Results and discussion

### Temperature, pH, redox potential and electronic conductivity comparisons

The influent water quality was illustrated in Online Resource 2, while Online Resource 3 shows the effluents characteristics of both greywater types. The mean values of inflow temperature were between 16.9 °C and 17.7 °C for HC–SGW and LC–SGW. The average pH value was 8.4 ± 1.61 for influent HC–SGW. In comparison, the mean pH was almost neutral (6.9 ± 0.48) for the influent LC–SGW. It was clearly noted that the pH correlated negatively and significantly (r = − 0.967; *p* < 0.001) with Eh in all treatment systems, which is common in aqueous ecosystems [52]. The cement–ochre pellets utilised in the current investigation had pH entries between 9.63 and 12.53 and redox potentials between −248.5 and − 98.8 mV. The mean electric conductivity for those pellets ranged between 1580 and 2300 μS/cm. Therefore, ochre pellets placed in the treatment systems always significantly increased pH and EC of the outflows. This can be explained by, for example, a positive significant correlation (r = 0.717, *p* = 0.030) between pH and Ca. However, the existence of *P. australis* in the purification of SGW significantly (*p* < 0.05) decreased the pH value of the outflow compared to the inflow pH. The drop in pH can be explained by the production of carbon dioxide during rhizome breathing and/or organic acids [14], which are by–products of biodegradation of organic substances in water by microorganisms [52, 53].

In this study, the statistical analysis showed that it is a challenge for *P. australis* to cope with the effect of ochre pellets in terms of pH and EC. An increase in HRT when treating SGW in wetland systems using a combination of *P. australis* and ochre pellets raised the pH values significantly, because of the presence of ochre pellets when comparing T2 and T6 with T10 and T14, respectively (Online Resource 4). This can be explained by the fact that ochre is a mineral-based sludge [25]; its presence in wastewater causes a dynamic chemical exchange of various ions, subsequently increasing the EC and pH [17, 54].

### Turbidity, total suspended solids and colour evaluations

Outflows of all systems were agitated before sampling to evaluate real removal mechanisms (without the impact of sedimentation) by *P. australis*, ochre or their combination. Because of the relatively high level of pH associated with using ochre pellets in contact with water in systems T4, T8, T12 and T16, it could be that the precipitation of dissolved solids in SGW and ochre pigments resulted in a significant increase (*p* < 0.05) in turbidity, TSS and colour within the outflow compared to the inflow [54, 55]. The statistical analysis showed pH correlated positively and significantly with turbidity (r = 0.700, *p* = 0.036), TSS (r = 0.950, *p* < 0.001) and colour (r = 0.783, *p* = 0.013). In comparison, the existence of *P. australis* in floating systems treating SGW (T1, T5, T9 and T13) resulted in a significant drop in turbidity, TSS and colour (Table 1).

**Table 1:** Significant differences when comparing accumulated concentrations (mg/kg) of trace elements in ochre pellets before and after treating synthetic greywater (SGW) in the treatment systems (T)

	HC–SGW^a^											
	Concentrations in ochre pellets before treatment compared to:											
		2–day HRT^b^							7–day HRT^b^			
	T2^c^			T4^d^			T10^e^			T12^f^		
Element	Shapiro–Wilk (*p* value)	Statistical test^g^	Significance (*p* value)	Shapiro–Wilk (p value)	Statistical test^g^	Significance (p value)	Shapiro–Wilk (p value)	Statistical test^g^	Significance (p value)	Shapiro–Wilk (p value)	Statistical test^g^	Significance (p value)
Aluminium	0.131	T–test	<0.001	0.546	T–test	0.009	0.252	T–test	0.003	0.386	T–test	<0.001
Boron	0.102	T–test	<0.001	0.290	T–test	<0.001	0.071	T–test	<0.001	0.053	T–test	<0.001
Calcium	0.123	T–test	<0.001	0.016	M–W	<0.001	0.019	M–W	<0.001	0.908	T–test	<0.001
Cadmium	<0.001	M–W	<0.001	<0.001	M–W	<0.001	<0.001	M–W	<0.001	<0.001	M–W	<0.001
Chromium	<0.001	M–W	<0.001	<0.001	M–W	<0.001	<0.001	M–W	<0.001	0.002	M–W	<0.001
Copper	<0.001	M–W	<0.001	<0.001	M–W	0.003	<0.001	M–W	0.001	<0.001	M–W	0.002
Iron	0.136	T–test	<0.001	0.374	T–test	0.013	0.324	T–test	<0.001	0.062	T–test	<0.001
Magnesium	<0.001	M–W	<0.001	<0.001	M–W	<0.001	<0.001	M–W	<0.001	<0.001	M–W	<0.001
Manganese	0.062	T–test	<0.001	0.011	M–W	<0.001	0.190	T–test	<0.001	0.003	M–W	<0.001
Nickel	0.393	T–test	0.441	0.006	M–W	0.045	0.045	M–W	0.103	0.023	M–W	0.018
Zinc	0.003	M–W	<0.001	<0.001	M–W	0.002	<0.001	M–W	<0.001	<0.001	M–W	<0.001
	LC–SGW^h^											
	Concentrations in ochre pellets before treatment compared to:											
		2–day HRT^b^							7–day HRT^b^			
	T6^i^			T8^j^			T14^k^			T16^l^		
Element	Shapiro–Wilk (*p* value)	Statistical test^g^	Significance (*p* value)	Shapiro–Wilk (p value)	Statistical test^g^	Significance (p value)	Shapiro–Wilk (p value)	Statistical test^g^	Significance (p value)	Shapiro–Wilk (p value)	Statistical test^g^	Significance (p value)
Aluminium	0.042	M–W	<0.001	0.089	T–test	<0.001	0.103	T–test	<0.001	0.022	M–W	<0.001
Boron	0.057	T–test	0.281	0.014	M–W	0.539	0.223	T–test	0.014	0.116	T–test	0.013
Calcium	0.119	T–test	<0.001	0.771	T–test	<0.001	0.132	T–test	<0.001	0.604	T–test	<0.001
Cadmium	<0.001	M–W	0.164	<0.001	M–W	0.556	<0.001	M–W	0.006	<0.001	M–W	0.047
Chromium	<0.001	M–W	0.710	<0.001	M–W	0.082	<0.001	M–W	<0.001	<0.001	M–W	<0.001
Copper	<0.001	M–W	0.056	<0.001	M–W	0.019	<0.001	M–W	0.006	<0.001	M–W	0.014
Iron	0.111	T–test	<0.001	0.041	M–W	<0.001	0.142	T–test	<0.001	0.087	T–test	<0.001
Magnesium	<0.001	M–W	<0.001	<0.001	M–W	<0.001	<0.001	M–W	<0.001	<0.001	M–W	<0.001
Manganese	0.134	T–test	<0.001	0.190	T–test	<0.001	0.193	T–test	<0.001	0.140	T–test	<0.001
Nickel	0.275	T–test	0.742	0.605	T–test	0.166	0.449	T–test	0.124	0.027	M–W	0.073
Zinc	<0.001	M–W	0.001	<0.001	M–W	<0.001	<0.001	M–W	<0.001	<0.001	M–W	<0.001

^a^HRT, hydraulic retention time

^b^HC-SGW, high concentration synthetic greywater

^c^T2, HC–SGW treatment systems 2-day with floating *P. australis* and ochre pellets

^d^T4, HC–SGW treatment systems with ochre pellets only

^e^T10, HC-SGW treatment systems 7-day with floating *P. australis* and ochre pellets

^f^T12, HC–SGW treatment systems with ochre pellets only

^g^Shapiro–Wilk (check for normality), normally distributed data, if *p* > 0.05 using T–test, and non–normally distributed data, if *p* < 0.05 using Mann–Whitney U–test; *p* value, significantly different, if p < 0.05, and not significantly different, if p > 0.05; and M–W, Mann–Whitney U–test

^h^LC-SGW, low concentration synthetic greywater

^i^T6, LC-SGW treatment systems 2-day with floating *P. australis* and ochre pellets

^j^T8, LC–SGW treatment systems with ochre pellets only

^k^T14, LC-SGW treatment systems 7-day with floating *P. australis* and ochre pellets

^l^T16, LC–SGW treatment systems with ochre pellets only

Biofilms that developed on the root and rhizome systems of *P. australis* and on the vessel walls led to improved biological decomposition enhancing the removal of TSS, turbidity and colour [14, 56]. The correlations between turbidity and TSS, turbidity and colour, and TSS and colour were positive and significant; r = 0.783 (p = 0.013), r = 0.767 (*p* = 0.016), and r = 0.767 (p = 0.016), respectively. However, on some occasions, the sloughing–off the heavy biofilms from rhizome surfaces in systems of floating macrophytes may have caused an increase in TSS concentrations [42]; e.g., comparison of the outflows of T2 to T4. Furthermore, the only significant effect of elevating HRT of the purification of SGW was a decrease in TSS and colour in systems with a combination of ochre pellets and *P. australis* (T10 and T14) compared with systems a HRT of 2 days (T2 and T6, correspondingly) (Online Resource 4).

### Dissolved oxygen, biochemical and chemical oxygen demand comparisons

**Fig. 2:**
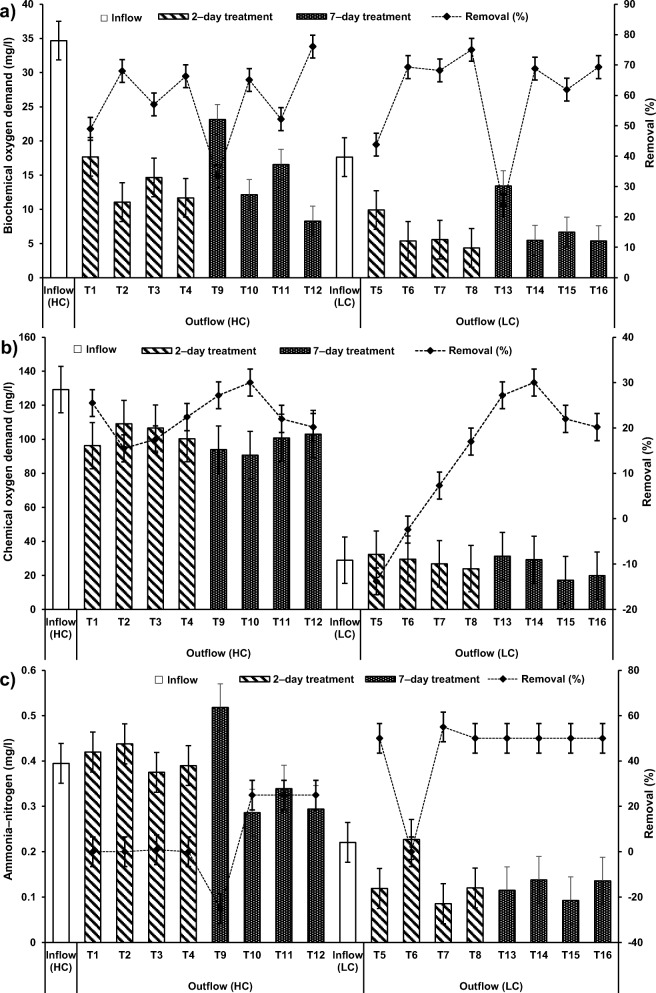

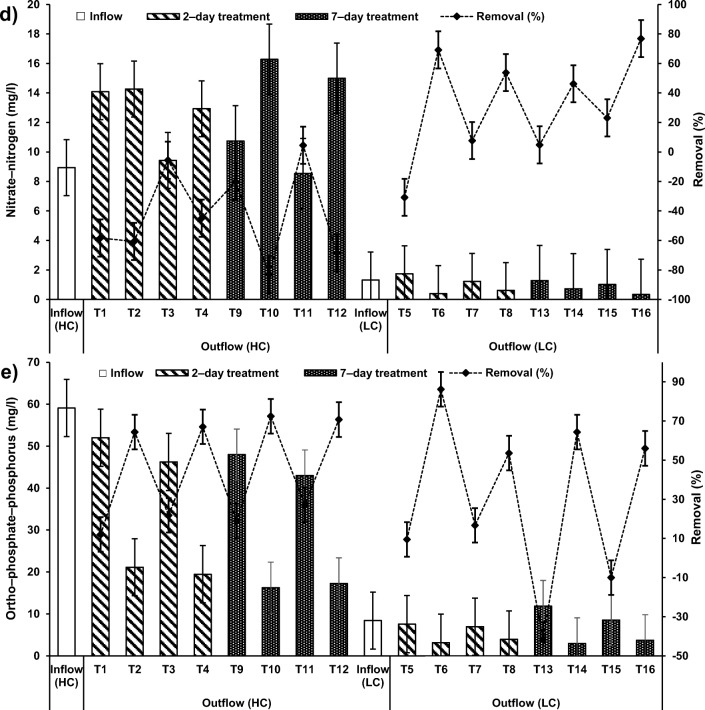
Overall performance of the floating treatment wetlands with different operational parameters for the treatment of both concentrations of synthetic greywater for 2– and 7–day hydraulic retention time to remove (**a**) biochemical oxygen demand, **b** chemical oxygen demand, **c** ammonia–nitrogen, **d** nitrate–nitrogen, and (**e**) ortho–phosphate–phosphorus

In this investigation, dissolved oxygen (DO), 5–day biochemical oxygen demand (BOD) and chemical oxygen demand (COD) decreased significantly at the presence of *P. australis* in treatment systems of both types of SGW (T1, T5, T9 and T13) compared with the outflows of systems T3, T7, T11 and T15, respectively (Online Resource 3). In terms of DO, these findings confirmed the other published findings by previous researchers [53]. However, it has been reported that the presence of macrophytes has only a minor impact on effluent DO values but a significant impact on BOD removal in experimental wetlands [57]. Furthermore, the BOD removals (Fig. 2a) in vegetated wetlands were reported to be higher than those removals in non − vegetated wetlands [37], while COD removals (Fig. 2b) have been successfully reduced in vegetated horizontal flow wetlands compared to those removals in non − vegetated wetlands [10].

**Fig. 3:**
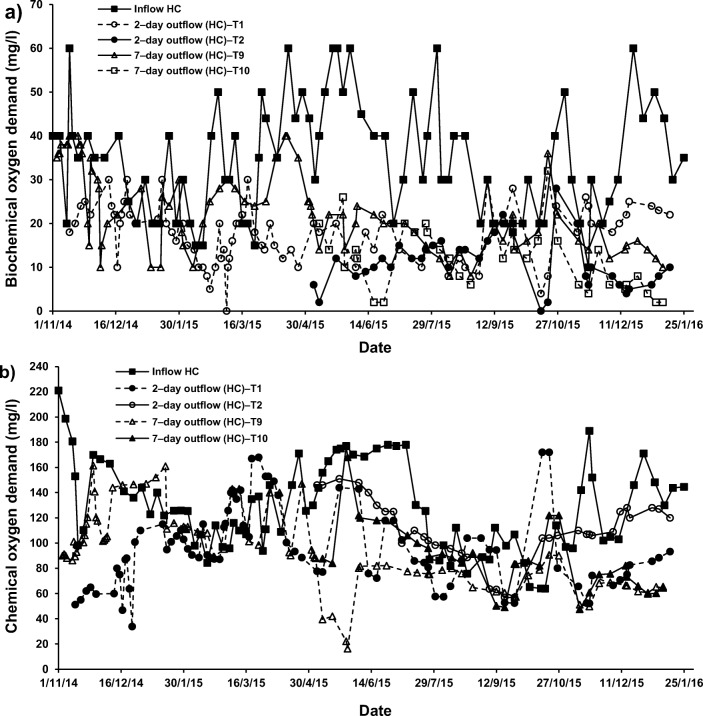

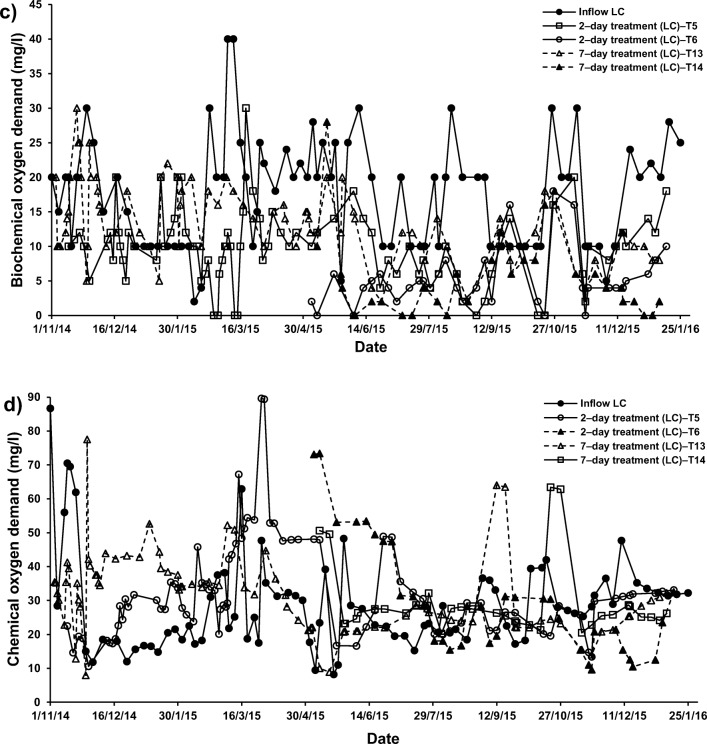
Effect of cement–ochre pellets and treatment hydraulic retention time (HRT) on the variations of **a**) 5 − day biochemical oxygen demand (BOD) concentrations in high contamination (HC) synthetic greywater, **b** chemical oxygen demand (COD) concentrations in HC synthetic greywater, **c** BOD concentrations in low contamination (LC) synthetic greywater, and **d**) COD concentrations in LC synthetic greywater

Compared to the inflow, the DO, BOD and COD concentrations were found to be significantly low in all systems treating HC–SGW for all HRT and systems applying a combination of ochre and plants (T2 with a HRT of two days) and T10 (7–day HRT)), as evident from Online Resource 4, Fig. 3a, b. The treatment of LC–SGW in systems applying a combination of pellets with *Phragmites* showed significant decreases in DO and BOD concentrations (Fig. 3). Similar effects on DO, BOD and COD were noted when ochre pellets were present in combination with *P. australis* in systems T6 and T14, if they were compared to those outflow values of systems T5 (2–day; only *P. australis*) and T10 (7–day; *P. australis*), respectively (Fig. 3).

**Fig. 4:**
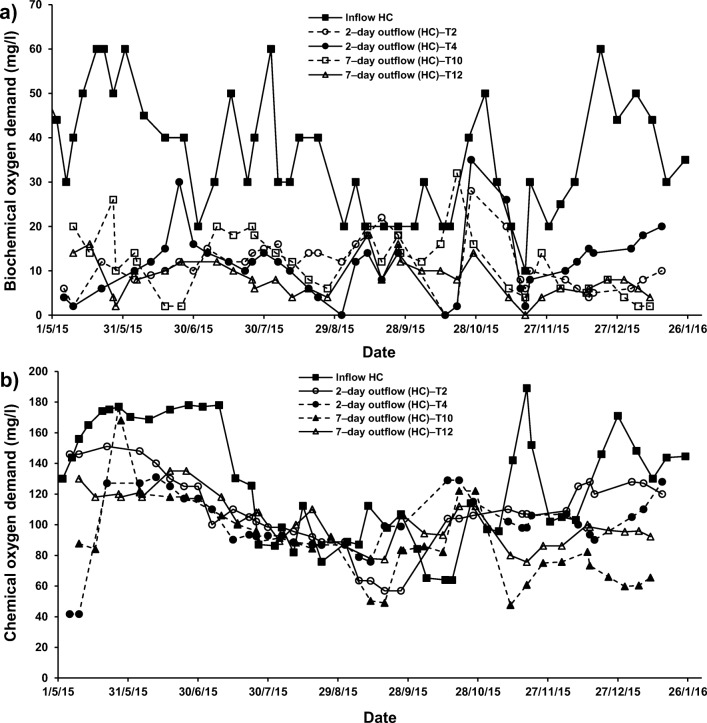

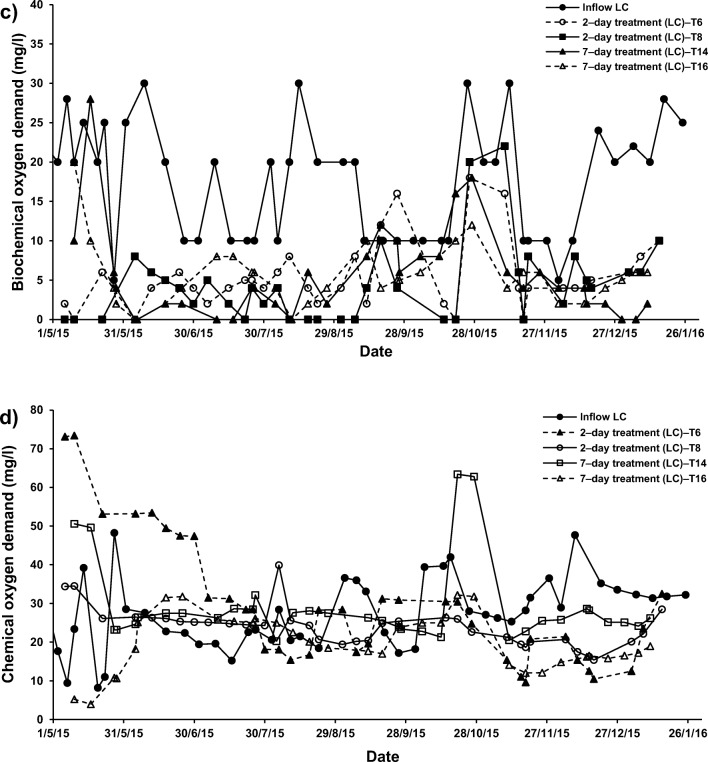
Effect of *Phragmites australis* and treatment hydraulic retention time (HRT) on the variation of; **a** 5 − day biochemical oxygen demand (BOD) concentrations in high contamination (HC) synthetic greywater; **b** chemical oxygen demand (COD) concentrations in HC synthetic greywater; **c** BOD concentrations in low contamination (LC) synthetic greywater; and **d**) COD concentrations in LC synthetic greywater

In addition, the occurrence of pellets alone in treatment of SGW (T4, T8, T12 and T16) also resulted in significant decreases in DO, BOD and COD when comparing the inflow equivalents (fills) with the outflow equivalents (draws) of the batch flow systems T3, T7, T11 and T15, respectively (Fig. 4). The significant reduction in DO and COD associated with the existence of ochre pellets was essentially due to the oxidation mechanisms from Fe, Al and Ca sources in greywater [46, 55].

The DO, BOD and COD in this study were not affected by the increase in HRT in system T14 (7–day; combination of ochre pellets and *P. australis*) compared with those values in the effluent of system T6 (2–day; combination of ochre pellets with *P. australis*), as shown in Figs. 3 and 4. A surge in HRT results in an elevation in DO [47]. Furthermore, more HRT for floating reed systems reduces their BOD removal [57].

Significant (*p* < 0.05) negative correlations were noted between pH and BOD (r = −0.983, *p* < 0.001). The strong negative correlation between pH and BOD may occur through elimination of organic substances in settlement and biological degradation processes [58], which consumes carbon and nutrient compounds leading to a surge in pH due to the release of carbon dioxide during cell growth [59].

However, there were considerable correlations between DO and COD, which were negative and significant (r = −0.700, *p* = 0.036) in the treatment of LC–SGW and positive and significant (r = 0.667, *p* = 0.050) for HC–SGW. Significant (*p* < 0.05) and negative correlations between DO and COD and/or BOD were recorded for systems with no limited oxygen supply, where aerobic biodegradation was a function of the amount of organic substances during the chemical oxidation process. For greywater, industrial wastewater and storm water, DO usually correlates negatively with COD. This is due to the lack of provision of dissolved organic matter. Aerobic biodegradation is not restricted by DO provision [41, 57]. Concerning significantly (*p* < 0.05) positive correlations between DO and COD, the adequate availability of oxygen to aerobic heterotrophic bacteria will considerably elevate aerobic biochemical oxidation [41].

### Nutrient assessments

The considerable significant (*p* < 0.05) decreases of PO_4_–*P* values in treatment systems containing only SGW (T3, T7, T11 and T15) compared to values of inflow were mainly due to either sedimentation and/or microorganism mechanisms (Fig. 2c–e). However, there was a nutrient imbalance for LC–SGW, which was not sufficient for the organisms’ survival and consequently limited PO_4_–P removal [13, 14]. Therefore, the outflow from treated SGW systems was agitated in this study before sampling to encourage solids to suspend or dissolve [16, 38].

The comparative statistical analysis (Online Resource 4) of inflow with outflow of system T2 treating HC–SGW by both ochre pellets and *P. australis* for 2–day HRT (Fig. 2d, e) showed that NO_3_–N was significantly (*p* < 0.05) elevated and PO_4_–P was significantly (p < 0.05) reduced than those corresponding values of the influent. These observations were similar when comparing inflow with outflow HC–SGW from systems T1 (2–day; only *P. australis*) and T4 (2–day; only cement−ochre pellets). Therefore, the significant (*p* < 0.05) decrease of PO_4_–P concentration was linked to the effect of the existence of cement−ochre pellets together with *P. australis* in system T2 compared to system T1.

**Fig. 5:**
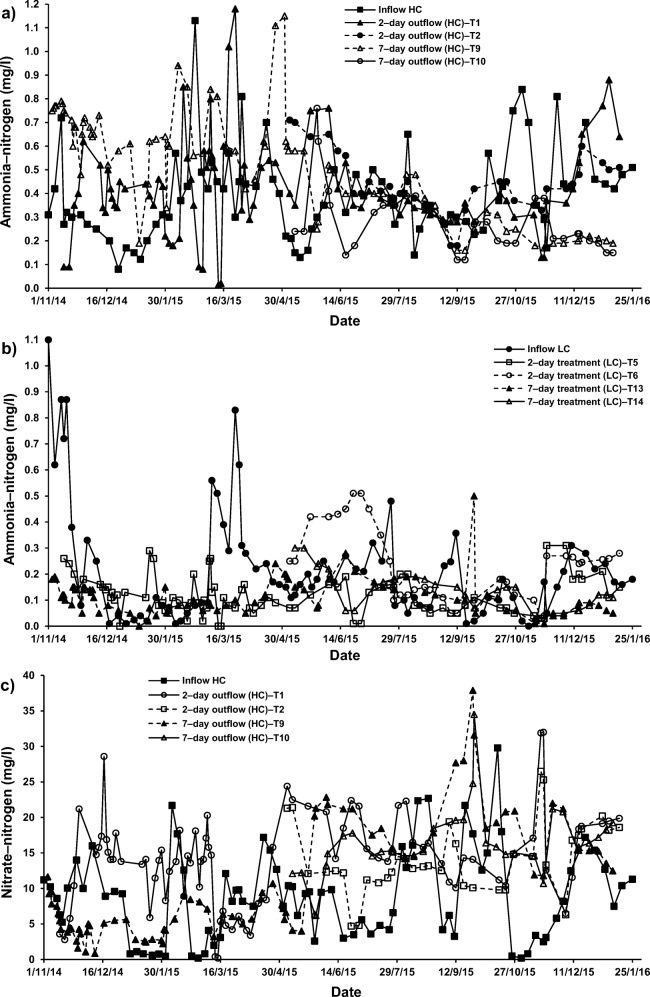

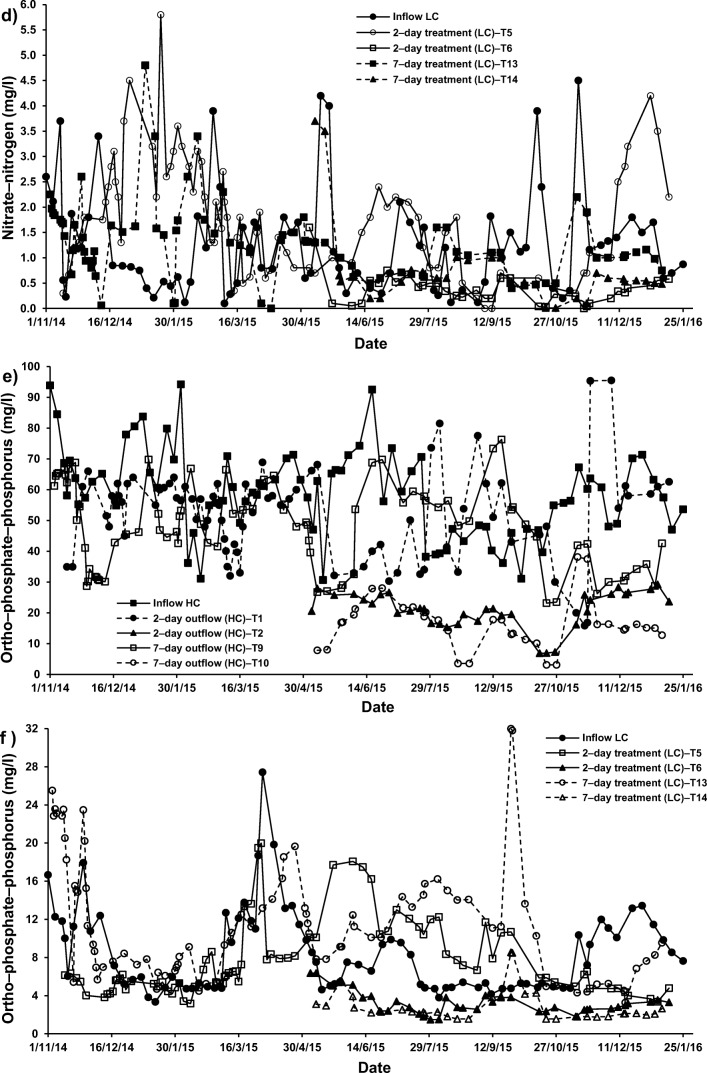
Effect of cement–ochre pellets and hydraulic retention time (HRT) on treatment of; **a** high concentration synthetic greywater (HC–SGW) in terms of the variation in ammonia-nitrogen (NH_4_–N), **b** low concentration synthetic greywater (LC–SGW) in terms of the variation in NH_4_–N, **c** HC–SGW in terms of the variation in nitrate-nitrogen (NO_3_–N), **d** HC–SGW in terms of the variation in NO_3_–N, **e** HC–SGW in terms of the variation in ortho-phosphate-phosphorus (PO_4_–P), and **f**) HC–SGW in terms of the variation in PO_4_–P

**Fig. 6:**
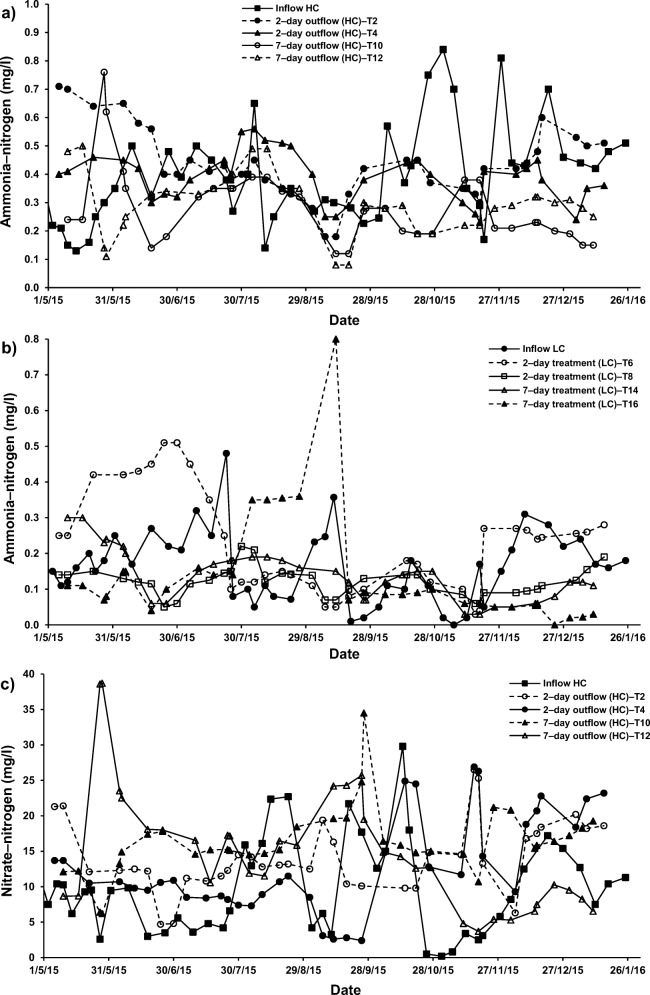

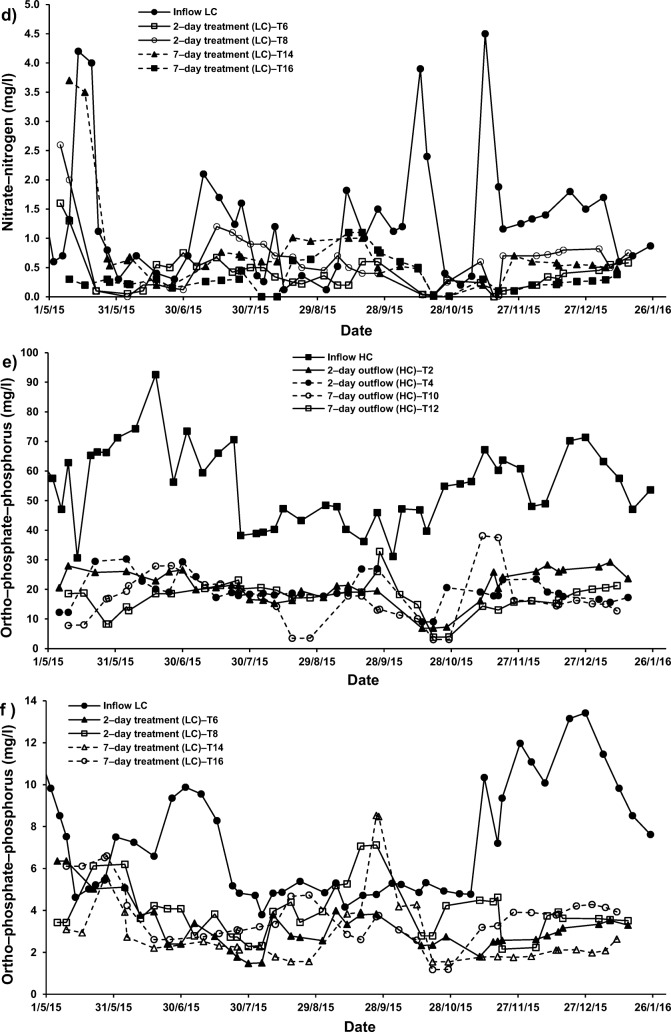
Effect of *Phragmites australis* and hydraulic retention time (HRT) on treatment of; **a** high concentration synthetic greywater (HC–SGW) in terms of the variation in ammonia-nitrogen (NH_4_–N), **b** low concentration synthetic greywater (LC–SGW) in terms of the variation in NH_4_–N, **c** HC–SGW in terms of the variation in nitrate-nitrogen (NO_3_–N), **d** LC–SGW in terms of the variation in NO_3_–N, **e** HC–SGW in terms of the variation in ortho-phosphate-phosphorus (PO_4_–P), and **f**) LC–SGW in terms of the variation in PO_4_–P

For systems applying both cement–ochre pellets and *P. australis* treating LC–SGW (T6 (2–day) and T14 (7–day HRT)), values of NO_3_–N and PO_4_–P were significantly (*p* < 0.05) reduced compared to those of the influent. Nevertheless, no significant (*p* > 0.05) alterations in concentrations of NH_4_–N were noted for those systems in comparison to the NH_4_–N values linked to the inflow (Fig. 2c, 5b and 6b). Considering that for systems treating SGW with only floating *P. australis*, volatilisation and plant uptake are primary elimination processes for NH_4_–N in floating treatment wetlands, the reduction of NO_3_–N and organic–N could cause ammonia generation, which may be less than the substantial increases of NH_4_–N [53].

The precipitation of PO_4_–P is a significant (p < 0.05) phosphorus removal mechanism in wetland systems [60]. The uptake by plants is another likely process of PO_4_–P reduction. Nevertheless, this is a seasonal phenomenon, which reverses when the plants are perishing in autumn [59]. Plants take–up phosphorus and nitrogen as essential nutrients [10]. However, the amount of phosphorus stored within plants is significantly (*p* < 0.05) less compared to that for nitrogen [61].

A significant contribution to PO_4_–P removal into FTWs is the existence of cement−ochre pellets in the treatment process, which is linked to either adsorption processes and/or precipitation mechanisms. This is because of to the relatively high amount of Ca that converts dissolved PO_4_–P to insoluble forms, if the pH is rather high [23, 55], as shown in Figs. 5 and 6. Although calcium phosphate precipitation is commonly the predominant removal mechanism, it has been reported that it could be less dominant for ochre−based sludge [46]. However, PO_4_–P concentrations correlated significantly negatively with pH values (r = −0.767, *p* = 0.016) and Ca concentrations (r = −0.783, *p* = 0.013) in the outflow of treatment systems. Furthermore, the outflow of the treatment systems combining ochre pellets and *P. australis* showed that TSS correlated significantly negatively (r = −0.717. *p* = 0.030) with PO_4_–P and significantly positively (r = 0.717, p = 0.030) with NO_3_–N.

After 7 days of treating HC–SGW in systems T10 (ochre pellets with *P. australis*) and T12 (only ochre pellets), NH_4_–N and PO_4_–*P* values decreased significantly (*p* < 0.05). However, NO_3_–N increased significantly (p < 0.05) in comparison to those parameters of the influent (Online Resource 4). Furthermore, the outflow of system T1 (only *P. australis*) showed the same patterns concerning NH_4_–N and PO_4_–P. Also, for a HRT of 7 days concerning the purification of HC–SGW, the existence of both cement−ochre pellets and floating *P. australis* in system T10 significantly (*p* < 0.05) increased NH_4_–N and PO_4_–P concentrations compared with the outflow of system T9 (only *P. australis*).

Regarding the increases in NO_3_–N concentrations, it was claimed that greywater is usually deficient in biodegradable organic matter including nutrients [57]. This has commonly a negative effect on the treatment performance [1, 13]. It follows that nitrification is a less relevant process in FTWs, because of few nitrifying organisms being present in the facultative neighbourhood and easy nitrogen removal by wetland vegetation [59]. Therefore, the existence of both cement−ochre pellets and floating *P. australis* for the purification of LC–SGW significantly (p < 0.05) decreased NO_3_–N and PO_4_–P within the outflow from systems T6 (2–day) and T14 (7–day) with no significant (*p* > 0.05) impact on NH_4_–N values, if compared to those values of outflow from systems with only *P. australis*; T5 (2–day) and T13 (7–day), respectively (Fig. 5). In addition, an elevated HRT for the cleaning of HC–SGW (Figs. 5 and 6) in systems applying both pellets and plants significantly (*p* < 0.05) reduced NH_4_–N and PO_4_–*P* values and significantly (*p* < 0.05) surged the NO_3_–N concentration in comparison to the system of 2–day HRT. Moreover, a higher HRT for the purification of LC–SGW (Figs. 5 and 6) in system T14 significantly (*p* < 0.05) decreased NH_4_–N and significantly (p < 0.05) increased NO_3_–N. Removal of NO_3_–N in systems treating LC–SGW with a combination of ochre pellets and *P. australis* were significantly greater than those reductions in treatment of HC–SGW.

In this evaluation, cement–ochre pellets can continue removing phosphorus in wetlands up to 7 days. In comparison, non − pelletized ochre sludge reaches an equilibrium in a phosphate−based solution after a HRT of roughly one hour [46]. Oxides and hydroxides of Fe and Al that are released from cement−ochre pellets and Ca compounds adsorb phosphorus according to Heal et al. [54]. Phosphorus can decrease quickly, if the water column is not stirred. It follows that an elevation of the HRT may not lead to a reduction in PO_4_–P at the company of cement−ochre pellets (Online Resource 4).

### Assessment of trace elements in greywater

In the system treating HC–SGW by an arrangement of both cement−ochre pellets and *P. australis* for a HRT of two days (T2), the outflow exhibited significant (*p* < 0.05) reductions in B, Cd, Cr, Cu, Fe, Mg, Mn, Ni and Zn in comparison to those corresponding concentrations of the inflow (Online Resource 3, Fig. 7a, b). However, a significant (*p* < 0.05) elevation in concentrations of Ca was observed in comparison to the inflow HC–SGW. Similar significant (*p* < 0.05) changes were observed for the effluent of system T1 (HC–SGW; 2–day; only *P. australis*) with the exception that Al and K showed significant (p < 0.05) decreases. System T4 (HC–SGW; 2–day; only ochre pellets) showed significant (*p* < 0.5) rises in Ca and Cu, and decreases in B, Mg, Mn and Ni.

**Fig. 7:**
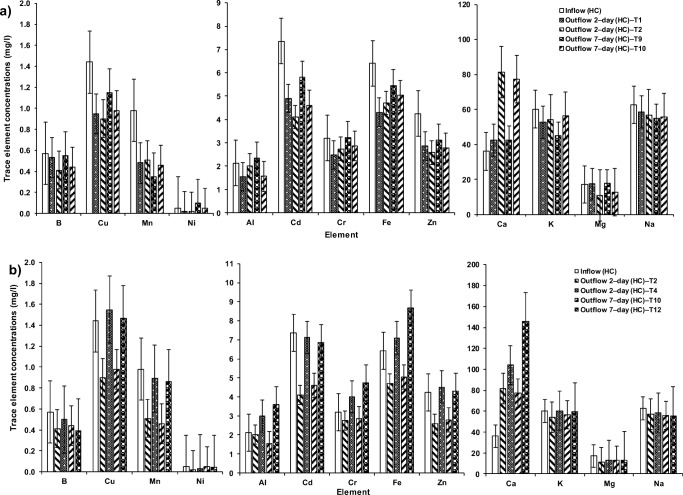

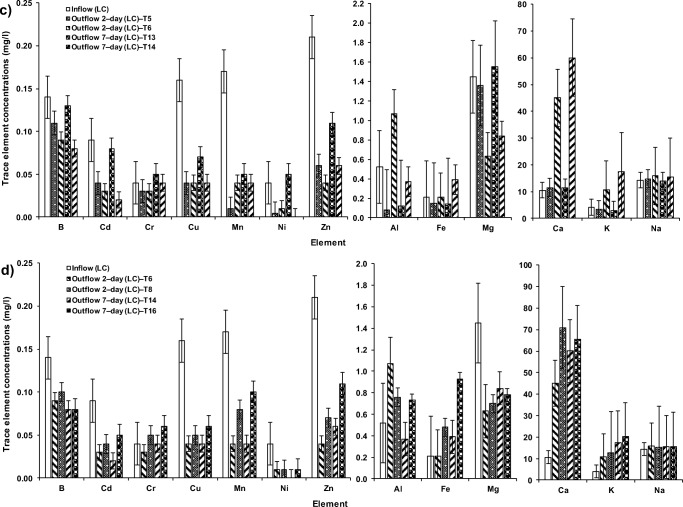
Variations in trace element concentrations within floating wetlands under the effect of **a**) cement–ochre pellets and hydraulic retention time (HRT) on high concentration synthetic greywater (HC–SGW) treatment, **b**) *Phragmites australis* and contact time on HC–SGW treatment, **c**) cement–ochre pellets and HRT on low concentration synthetic greywater (LC–SGW) treatment, and **d**) *P. australis* and HRT on LC–SGW treatment

For FTWs, trace elements might be removed by processes such as settlement, sedimentation, biological sorption, precipitation, cation exchange, photo−degradation, biological degradation, microbial processes and uptake by plants. However, the actual reactions cannot easily be determined [32, 62].

Concerning this investigation, a comparative analysis between outflows of systems T1 and T2 showed that the existence of both pellets and plants in system T2 significantly (*p* < 0.05) reduced B and Mg, and increased Al, Ca and Fe in the outflows. Those significant increases were linked to high Al, Ca and Fe concentrations within the pellets, which were discharged back into the treated greywater [54]. On the other hand, a comparison between outflows of systems T2 and T4 revealed that *P. australis*, when used in combination with pellets in system T2, significantly (*p* < 0.05) decreased the concentrations of all considered elements (Online Resource 4).

Concerning the treatment of HC–SGW for a HRT of 7 days when combining ochre pellets and floating *P. australis*, the outflow of system T10 concerning Al, B, Cd, Cu, Fe, Mg, Mn and Zn reduced significantly (*p* < 0.05) with a significant rise in Ca concentration compared to those corresponding concentrations of the influent (Fig. 7a). Furthermore, for a HRT of 7 days, the pellets and floating plants in system T10 (HC–SGW) significantly (*p* < 0.05) affected the removal of Al, B, Cd, Cr, Cu, Mg, Ni and Zn compared with those concentrations in the outflow from system T9. While floating *P. australis* in combination with ochre pellets in system T10 contributed significantly (p < 0.05) to the decrease of Al, Ca, Cd, Cr, Cu, Fe, K, Mg, Mn and Zn values compared with the outflows of system T12. However, system T9 (7–day; HC–SGW; only *P. australis*) showed significant (p < 0.05) reductions of Cu, K, Mn, Ni and Zn compared to the inflow (Fig. 7a, b). System T12 (7–day; HC–SGW; only ochre pellets) removed B and Mg significantly (*p* < 0.05) in comparison to their inflow concentrations. In Online Resource 4, the role of plants in obtaining nutrients straight from the greywater was evidenced [16]. In parallel, dissolved elements in greywater such as Al, Ca and Fe might be adsorbed chemically to replace vacant places, which were occupied by the over−saturated elements in ochre pellets and then remobilised to the water [24, 63].

For the treatment of LC–SGW, if pellets and plants are used in system T6 subjected to a HRT of 2 days, the B, Cd, Cu, Mg, Mn, Ni and Zn concentrations were significantly (*p* < 0.05) decreased in comparison to those in the influent. However, there were significant (*p* < 0.05) increases in Al, Ca, Fe and K in the outflow of system T6 in comparison with those in the influent. Similar significant changes as in system T6 were observed in outflows of systems T5 (2–day; LC–SGW; only floating *P. australis*) and T8 (2–day; LC–SGW; only ochre pellets), with the exception for significant decreases in Al, Fe and K for system T6, compared to those values of the inflow (Fig. 7c, d). So, combined ochre pellets and floating plants in system T6 had a significant (*p* < 0.05) impact on the decrease in the B, Cd and Zn values, and the increase in Al, Ca, K, Mn and Ni concentrations compared to the outflow ones of system T5. Furthermore, the presence of floating *P. australis* contributed significantly to the reduction of B, Ca, Cd, Cr, Cu, Fe, Mn, Ni and Zn compared with the outflow from system T8 (Fig. 7c).

The purification of LC–SGW after 7 days of HRT, combining ochre pellets and floating *P. australis* in system T14 significantly (*p* < 0.05) reduced concentrations of B, Cd, Cu, Mg, Mn, Ni and Zn and significantly (p < 0.05) increased Ca, Fe and K in comparison to those corresponding values of the influent. The same significant performance as for the outflow of T14 was noted for the outflow of system T13 (7–day; LC–SGW; only floating *P. australis*), but Al, Fe and K were lower, and Mg and Ni were higher than those of the influent. Furthermore, significant (p < 0.05) changes in the outflow of system T16 (7–day; LC–SGW; only ochre pellets) were noted. These were like those changes in the outflow of system T14, except that a significant elevation in Al was recorded for the effluent of the former system (T16) compared with the inflow (Fig. 7d). Therefore, the existence of both ochre and plants in system T14 had a significant (*p* < 0.05) impact on the decrease in B, Mg and Zn, and on the increase in Al, Ca, Fe and K compared with those concentrations of the outflow of system T13. The existence of plants in T14 significantly (*p* < 0.05) affected the decrease in Al and Zn compared to the outflow of system T16 (Online Resource 4).

Concerning the purification of LC–SGW when combining pellets and floating *P. australis* in system T14, an increasing HRT significantly decreased Al, B and Ni, while it increased Ca, Fe and Mg significantly (p < 0.05) in comparison with the effluent of system T6 (2–day; LC–SGW; pellets with floating *P. australis*). All wetlands were impacted on by natural forces allowing dust and leaves to enter the systems by coincidence. Therefore, certain elements increased in the effluent of the wetlands without the existence of ochre as well [16, 32].

### Comparison of elements accumulated in pellets

After the end of the purification experiment, the accumulations of all elements in ochre pellets were significantly (*p* < 0.05) elevated except for Ca and Ni. Ca values were significantly (p < 0.05) reduced in pellets. The accumulated Ni concentrations did not increase significantly (*p* > 0.05) in pellets before (Online Resource 5) and after treatment in almost all treatment systems. Additionally, in systems T6 (ochre pellets and *P. australis*) and T8 (only ochre pellets) purifying LC–SGW for HRT of 2 days, the accumulated B, Cd, Cr and Cu also did not increase significantly (Table 1).

Concerning the treatment of HC–SGW for a HRT of 2 days, the existence of *P. australis* in combination with ochre pellets in system T2 significantly (*p* < 0.05) increased the Al, B, Ca, Cr, Cu, Fe and Zn concentrations, which accumulated in pellets in comparison to those in system T4 (2–day; HC–SGW; only ochre pellets). However, that effect was different for system T10 (HC–SGW; ochre pellets with floating *P. australis*) with a HRT of 7 days, where concentrations of Al, Ca and Cd were significantly (p < 0.05) reduced compared to elements, which accumulated in the pellets of system T12 (7–day; HC–SGW; only ochre pellets) as shown in Table 2. A noticeable relationship between the presence of *P. australis* and an increase in the accumulation rate of elements in ochre pellets within the same treatment system was noted (Online Resource 6a and b). This could be linked to the respiration of rhizomes and the production of carbon dioxide and acidic exudates, coupled with the production of organic acids during the biodegradation of organic substances by microorganisms [53, 58]. Those acidic by–products cause a significant (*p* < 0.05) lowering of the water pH, converting insoluble metals to their dissolved forms, which could be adsorbed by ochre pellets faster than they could be taken up by *P. australis* due to chemical reactions (Online Resource 6a and b).

**Table 2:** Significance values obtained from statistical analysis for digested ochre pellet samples to assess; (a) the effects of *Phragmites australis* on trace element accumulations (mg/kg) within ochre pellets, and 7(b) the effects of increased hydraulic retention time (HRT) and pollutant loads on trace element accumulations (mg/kg) within ochre pellets combined with *P. australis* in treatment systems (T)

(a) Effects of *P. australis* on trace element accumulations within ochre pellets in T												
	2–day HRT						7–day HRT					
	HC–SGW (T2^a^ & T4^b^)			LC–SGW (T6^c^ & T8^d^)			HC–SGW (T10^e^ & T12^f^)			LC–SGW (T14^g^ & T16^h^)		
Element	Shapiro–Wilk (p value)	Statistical test^i^	Significance (p value)	Shapiro–Wilk (p value)	Statistical test^i^	Significance (p value)	Shapiro–Wilk (p value)	Statistical test^i^	Significance (p value)	Shapiro–Wilk (p value)	Statistical test^i^	Significance (p value)
Aluminium	0.980	T–test	0.037	0.895	T–test	0.598	0.914	T–test	0.001	0.145	T–test	0.216
Boron	0.880	T–test	0.043	0.002	M–W	0.419	0.047	M–W	0.820	0.003	M–W	0.983
Calcium	0.956	T–test	0.010	0.208	T–test	<0.001	0.198	T–test	<0.001	0.384	T–test	<0.001
Cadmium	0.062	T–test	0.090	<0.001	M–W	0.511	0.796	T–test	<0.001	<0.001	M–W	0.168
Chromium	<0.001	M–W	0.014	<0.001	M–W	0.229	0.001	M–W	0.257	<0.001	M–W	0.009
Copper	<0.001	M–W	0.014	<0.001	M–W	0.473	<0.001	M–W	0.421	<0.001	M–W	0.431
Iron	0.511	T–test	0.008	0.185	T–test	0.06	0.156	T–test	0.459	0..549	T–test	0.554
Magnesium	<0.001	M–W	0.856	<0.001	M–W	0.122	<0.001	M–W	0.084	<0.001	M–W	0.164
Manganese	0.759	T–test	0.391	0.338	T–test	0.014	0.908	T–test	0.815	0.079	T–test	0.121
Nickel	0.009	M–W	0.166	0.902	T–test	0.303	0.054	T–test	0.507	0.250	T–test	0.279
Zinc	<0.001	M–W	0.022	<0.001	M–W	0.496	<0.001	M–W	0.083	<0.001	M–W	0.635
(b) Effects of HRT and pollutant load on trace element accumulations within ochre pellets in T												
	Effect of HRT						Effect of pollutant loads					
	HC–SGW			LC–SGW			2–day HRT			7–day HRT		
	(T2^a^ & T10^e^)			(T6^c^ & T14^g^)			(T2^a^ & T6^c^)			(T10^e^ & T14^g^)		
Element	Shapiro–Wilk (p value)	Statistical test^i^	Significance (p value)	Shapiro–Wilk (p value)	Statistical test^i^	Significance (p value)	Shapiro–Wilk (p value)	Statistical test^i^	Significance (p value)	Shapiro–Wilk (p value)	Statistical test^i^	Significance (p value)
Aluminium	0.966	T–test	0.103	0.946	T–test	0.673	0.842	T–test	0.008	0.604	T–test	<0.001
Boron	0.095	T–test	0.739	0.028	M–W	0.101	0.087	T–test	<0.001	0.174	T–test	<0.001
Calcium	0.688	T–test	0.002	0.899	T–test	0.631	0.656	T–test	0.152	0.525	T–test	<0.001
Cadmium	0.181	T–test	0.153	<0.001	M–W	0.038	0.001	M–W	<0.001	0.002	M–W	0.018
Chromium	<0.001	M–W	<0.001	<0.001	M–W	<0.001	<0.001	M–W	<0.001	<0.001	M–W	0.144
Copper	<0.001	M–W	0.223	<0.001	M–W	0.243	<0.001	M–W	0.005	<0.001	M–W	0.110
Iron	0.434	T–test	0.445	0.499	T–test	0.063	0.502	T–test	0.030	0.688	T–test	0.253
Magnesium	0.003	M–W	0.355	<0.001	M–W	0.154	0.476	T–test	<0.001	0.118	T–test	<0.001
Manganese	0.502	T–test	0.778	0.054	T–test	0.088	0.517	T–test	0.326	0.034	M–W	0.250
Nickel	0.017	M–W	0.253	0.451	T–test	0.222	0.189	T–test	0.649	0.064	T–test	0.522
Zinc	<0.001	M–W	0.194	<0.001	M–W	0.967	<0.001	M–W	0.167	<0.001	M–W	0.741

^a^T2, HC–SGW treatment systems 2-day with floating *P. australis* and ochre pellets

^b^T4, HC–SGW treatment systems 2-day with ochre pellets only

^c^T6, LC-SGW treatment systems 2-day with floating *P. australis* and ochre pellets

^d^T8, LC–SGW treatment systems 2-day with ochre pellets only

^e^T10, HC-SGW treatment systems 7-day with floating *P. australis* and ochre pellets

^f^T12, HC–SGW treatment systems 7-day with ochre pellets only

^g^T14, LC-SGW treatment systems 7-day with floating *P. australis* and ochre pellets

^h^T16, LC–SGW treatment systems 7-day with ochre pellets only

^i^Shapiro–Wilk (check for normality), normally distributed data, if p > 0.05 using T–test, and non–normally distributed data, if p < 0.05 using Mann–Whitney U–test; p value, significantly different, if p < 0.05, and not significantly different, if p > 0.05; and M–W, Mann–Whitney U–test

For the treatment of LC–SGW with a HRT of 2 days for system T6 (both pellets and plants), the existence of floating *P. australis* significantly (p < 0.05) affected the reduction in Ca and increase in Mn that enriched in pellets in comparison to those values in the pellets of system T8 (2–day; LC–SGW; only ochre pellets), as shown in Online Resource 6c and d. Regarding the purification of LC–SGW with systems of a HRT of 7 days, the presence of *P. australis* significantly (*p* < 0.05) affected the decrease in Ca and the increase in Cd and Cr in the ochre pellets of system T14 (combined pellets and plants) in comparison to the concentrations in the ochre pellets−based system T16 (2–day; LC–SGW; only ochre pellets), as shown in Tables 3 and 4. It is expected that significant (p < 0.05) decreases in the Ca content of ochre pellets is the main reason for significant increases in both pH value and Ca concentrations in the outflow SGW of systems containing ochre pellets [23, 46].

**Table 3:** The average concentrations (mg/kg) of detected trace elements, which accumulated in *Phragmites australis* located in the treatment system (T) at the end of the experiment, for (a) high pollutant concentration synthetic greywater (HC–SGW), (b) low pollutant concentration synthetic greywater (LC–SGW), and (c) control wetlands receiving tap water (TW)

a) HC–SGW																				
	2–day HRT^a^										7–day HRT^a^									
	Dry mass: 507.7 g						734.7 g				765.5 g					604.8 g				
Element	*P. australis* in T1^b^						*P. australis* in T2^c^				*P. australis* in T9^d^					*P. australis* in T10^e^				
	n^f^	Mean	SD^g^	Min^h^	Max^i^	n^f^	Mean	SD^g^	Min^h^	Max^i^	n^f^	Mean	SD^g^	Min^h^	Max^i^	n^f^	Mean	SD^g^	Min^h^	Max^i^
Aluminium (Al)	24	1421.9	249.13	1194.6	1873.8	24	245.6	73.37	128.0	389.4	24	530.1	69.76	422.0	700.9	24	284.2	68.23	228.0	520.3
Boron (B)	15	15.1	4.00	10.3	24.5	15	18.2	3.42	12.5	24.6	15	14.8	3.40	10.7	21.1	15	8.1	3.33	4.6	13.9
Calcium (Ca)	27	4001.2	432.00	3356.4	4856.4	27	8915.8	1363.03	6362.2	10,957.0	27	3996.0	214.81	3592.5	4450.6	27	6888.2	551.98	5978.8	8015.4
Cadmium (Cd)	18	1782.0	321.21	1301.7	2392.5	18	494.6	136.49	282.4	656.4	18	1293.2	146.24	1000.4	1541.1	18	467.1	113.89	316.5	663.5
Chromium (Cr)	27	1718.0	266.73	1389.0	2135.0	27	248.1	71.50	140.8	312.4	27	870.1	66.43	747.5	968.4	27	193.7	24.56	157.3	226.4
Copper (Cu)	27	477.2	74.23	394.3	597.5	27	106.2	29.60	62.1	137.3	27	347.0	28.27	302.3	398.1	27	101.3	14.07	83.6	126.4
Iron (Fe)	21	2799.4	418.29	2408.0	3414.4	21	508.7	144.41	296.4	639.5	21	2238.2	163.12	1997.1	2451.8	21	662.9	45.37	592.9	722.8
Magnesium (Mg)	27	1171.3	94.90	1020.9	1320.5	27	871.9	71.90	744.7	947.3	27	1284.6	52.18	1190.4	1382.3	27	1151.8	36.08	1077.7	1220.6
Manganese (Mn)	27	533.1	74.49	450.3	649.8	27	341.5	66.43	236.2	403.5	27	501.1	30.08	453.1	539.1	27	167.2	20.23	141.0	213.2
Sodium (Na)	12	1735.2	96.91	1602.1	1886.3	12	1787.9	62.90	1677.6	1875.6	12	1930.3	111.33	1800.7	2119.4	12	1569.4	23.23	1515.1	1591.8
Nickel (Ni)	27	37.5	14.75	12.2	91.1	27	20.8	13.31	2.2	60.5	27	90.0	12.77	61.2	130.0	27	24.8	11.34	0.0	54.8
Zinc (Zn)	24	1072.2	186.08	806.6	1467.2	24	333.4	115.66	194.6	730.4	24	699.9	72.98	551.8	872.1	24	241.9	71.35	155.8	494.5
b) LC–SGW																				
	2–day HRT										7–day HRT									
	Dry mass: 649.2 g					448.8 g					908.2 g					395.3 g				
Element	*P. australis* in T5^j^					*P. australis* in T6^k^					*P. australis* in T13^l^					*P. australis* in T14^m^				
	n^f^	Mean	SD^g^	Min^h^	Max^i^	n^f^	Mean	SD^g^	Min^h^	Max^i^	n^f^	Mean	SD^g^	Min^h^	Max^i^	n^f^	Mean	SD^g^	Min^h^	Max^i^
Aluminium (Al)	24	744.4	56.75	682.1	861.0	24	107.8	46.81	69.4	283.4	24	207.3	54.43	144.2	355.0	24	158.6	67.16	101.26	373.3
Boron (B)	15	7.6	4.00	3.6	15.1	15	4.4	2.34	1.6	8.6	15	7.8	4.32	3.6	16.7	15	5.0	2.54	2.16	9.8
Calcium (Ca)	27	3533.9	315.71	2976.5	3988.5	27	6516.4	355.10	5961.2	7196.9	27	3390.9	226.85	3064.6	3892.6	27	10,048.7	1048.10	7973.12	11,941.3
Cadmium (Cd)	18	443.9	38.05	368.8	501.3	18	45.2	19.39	5.3	76.4	18	215.3	34.66	149.8	274.7	18	44.7	17.20	6.74	75.9
Chromium (Cr)	27	109.8	5.43	98.7	119.8	27	35.8	3.21	30.0	44.4	27	51.6	11.22	38.4	72.5	27	22.5	4.23	16.80	32.7
Copper (Cu)	27	259.0	9.49	238.3	277.9	27	25.4	3.23	19.9	33.7	27	93.6	8.74	80.7	110.3	27	23.9	7.06	13.47	53.6
Iron (Fe)	21	1003.8	198.84	787.4	1285.0	21	307.0	9.68	284.8	325.7	21	466.8	67.52	398.5	569.0	21	268.6	10.14	247.01	284.8
Magnesium (Mg)	27	1089.7	59.19	1008.1	1204.5	27	468.4	15.23	432.4	488.5	27	1028.7	47.19	953.7	1125.0	27	452.8	29.91	391.18	488.2
Manganese (Mn)	27	335.8	50.54	260.1	392.7	27	64.3	11.36	47.8	107.9	27	257.2	21.88	235.9	294.2	27	63.3	9.58	53.87	89.8
Sodium (Na)	12	1069.9	39.44	983.1	1141.7	12	905.5	33.77	847.4	962.4	12	1192.6	26.15	1156.1	1232.1	12	1453.2	66.54	1340.67	1557.8
Nickel (Ni)	27	57.6	14.59	26.4	95.9	27	12.6	8.69	5.4	48.9	27	22.1	10.37	1.1	59.9	27	12.7	7.85	3.61	33.2
Zinc (Zn)	24	715.1	84.90	582.5	945.7	24	94.7	37.46	62.4	214.6	24	265.9	60.60	180.4	436.6	24	109.6	59.34	57.29	275.2
c) Roots and rhizomes analysis																				
	Dry mass: 856.3 g										567.8 g									
Element	2–day *P. australis* in C1^n^										7–day *P. australis* in C3^o^									
	n^f^		Mean		SD^g^		Min^h^		Max^i^		n^f^		Mean		SD^g^		Min^h^		Max^i^	
Aluminium (Al)	24		14.7		15.74		0.0		55.7		24		51.3		17.46		23.9		91.7	
Boron (B)	15		7.2		3.47		4.4		14.6		15		6.3		3.63		3.0		13.6	
Calcium (Ca)	27		3468.5		222.86		3041.0		3920.0		27		4284.6		267.06		3785.0		4831.2	
Cadmium (Cd)	18		16.7		16.66		0.0		48.3		18		23.1		17.68		0.0		65.8	
Chromium (Cr)	27		9.1		6.18		1.3		24.5		27		18.0		2.88		14.3		26.7	
Copper (Cu)	27		29.3		16.01		13.6		54.7		27		30.5		3.65		25.8		39.4	
Iron (Fe)	21		122.5		12.08		102.0		146.9		21		230.9		16.98		208.9		266.9	
Magnesium (Mg)	27		1092.7		61.33		971.6		1191.9		27		1185.1		82.97		1061.3		1337.6	
Manganese (Mn)	27		44.1		9.72		35.8		77.8		27		82.5		9.43		72.5		119.6	
Sodium (Na)	12		2969.3		177.33		2717.0		3280.8		12		2139.8		202.84		1939.8		2567.3	
Nickel (Ni)	27		9.2		13.58		0.2		66.7		27		12.2		15.02		0.0		63.0	
Zinc (Zn)	24		221.2		46.60		165.1		342.5		24		251.5		53.76		168.3		353.0	

^a^HRT, hydraulic retention time

^b^T1, HC–SGW treatment systems 2-day with only floating *P. australis*

^c^T2, HC–SGW treatment systems 2-day with floating *P. australis* and ochre pellets

^d^T9, HC-SGW treatment systems 7-day with only floating *P. australis*

^e^T10, HC-SGW treatment systems 7-day with floating *P. australis* and ochre pellets

^f^n, number of tested samples

^g^SD, standard deviation

^h^Min, minimum value

^i^Max, maximum value

^j^T5, LC-SGW treatment systems 2-day with only floating *P. australis*

^k^T6, LC-SGW treatment systems 2-day with floating *P. australis* and ochre pellets

^l^T13, LC-SGW treatment systems 7-day with only floating *P. australis*

^m^T14, LC-SGW treatment systems 7-day with floating *P. australis* and ochre pellets

^n^C1, control wetland containing floating *P. australis* in tap water at 2–day HRT

^o^C3, control wetland containing floating *P. australis* in tap water at 7–day HRT

**Table 4:** Significance values of statistical analysis for accumulated trace elements (mg/kg) in *Phragmites australis* tissue in floating treatment wetlands (T) concerning high (HC) and low (LC) pollutant concentrations within synthetic greywaters (SGW) compared with those elements accumulated in *P. australis* tissues of the corresponding control wetlands (C) for (a) 2–day hydraulic retention time (HRT), and (b) 7–day HRT

a) 2–day HRT												
	HC–SGW						LC–SGW					
	C1^a^ & T1^b^			C1^a^ & T2^c^			C1^a^ & T5^d^			C1^a^ & T6^e^		
Parameter	Shapiro–Wilk (*p* value)	Statistical test^f^	Significance (*p* value)	Shapiro–Wilk (p value)	Statistical test^f^	Significance (p value)	Shapiro–Wilk (p value)	Statistical test^f^	Significance (p value)	Shapiro–Wilk (p value)	Statistical test^f^	Significance (p value)
Aluminium	<0.001	M–W	<0.001	<0.001	M–W	<0.001	<0.001	M–W	<0.001	0.001	M–W	<0.001
Boron	0.029	M–W	<0.001	0.006	M–W	<0.001	<0.001	M–W	0.917	0.001	M–W	0.003
Calcium	0.002	M–W	<0.001	<0.001	M–W	<0.001	0.219	T–test	0.384	<0.001	M–W	<0.001
Cadmium	<0.001	M–W	<0.001	<0.001	M–W	<0.001	<0.001	M–W	<0.001	0.005	M–W	<0.001
Chromium	<0.001	M–W	<0.001	<0.001	M–W	<0.001	<0.001	M–W	<0.001	<0.001	M–W	<0.001
Copper	<0.001	M–W	<0.001	<0.001	M–W	<0.001	<0.001	M–W	<0.001	<0.001	M–W	0.082
Iron	<0.001	M–W	<0.001	<0.001	M–W	<0.001	<0.001	M–W	<0.001	<0.001	M–W	<0.001
Magnesium	0.064	T–test	0.001	0.015	M–W	<0.001	0.103	T–test	0.853	<0.001	M–W	<0.001
Manganese	<0.001	M–W	<0.001	<0.001	M–W	<0.001	<0.001	M–W	<0.001	<0.001	M–W	<0.001
Sodium	<0.001	M–W	<0.001	<0.001	M–W	<0.001	<0.001	M–W	<0.001	<0.001	M–W	<0.001
Nickel	<0.001	M–W	<0.001	<0.001	M–W	<0.001	<0.001	M–W	<0.001	<0.001	M–W	<0.001
Zinc	<0.001	M–W	<0.001	<0.001	M–W	<0.001	<0.001	M–W	<0.001	<0.001	M–W	<0.001
(b) 7–day HRT												
	HC–SGW						LC–SGW					
	C3^g^ & T9^h^			C3^g^ & T10^i^			C3^g^ & T13^j^			C3^g^ & T14^k^		
Parameter	Shapiro–Wilk (p value)	Statistical test^f^	Significance (p value)	Shapiro–Wilk (p value)	Statistical test^f^	Significance (p value)	Shapiro–Wilk (p value)	Statistical test^f^	Significance (p value)	Shapiro–Wilk (p value)	Statistical test^f^	Significance (p value)
Aluminium	<0.001	M–W	<0.001	0.001	M–W	<0.001	0.009	M–W	<0.001	<0.001	M–W	<0.001
Boron	0.029	M–W	<0.001	0.001	M–W	0.033	<0.001	M–W	0.152	<0.001	M–W	0.468
Calcium	0.715	T–test	<0.001	<0.001	M–W	<0.001	0.012	M–W	<0.001	<0.001	M–W	<0.001
Cadmium	<0.001	M–W	<0.001	<0.001	M–W	<0.001	<0.001	M–W	<0.001	0.278	T–test	0.001
Chromium	<0.001	M–W	<0.001	<0.001	M–W	<0.001	<0.001	M–W	<0.001	0.004	M–W	<0.001
Copper	<0.001	M–W	<0.001	<0.001	M–W	<0.001	<0.001	M–W	<0.001	0.001	M–W	<0.001
Iron	<0.001	M–W	<0.001	<0.001	M–W	<0.001	<0.001	M–W	<0.001	0.007	M–W	<0.001
Magnesium	0.073	T–test	<0.001	<0.001	M–W	0.303	0.007	M–W	<0.001	<0.001	M–W	<0.001
Manganese	<0.001	M–W	<0.001	<0.001	M–W	<0.001	<0.001	M–W	<0.001	0.001	M–W	<0.001
Sodium	0.021	M–W	0.013	0.002	M–W	<0.001	<0.001	M–W	<0.001	0.011	M–W	<0.001
Nickel	<0.001	M–W	<0.001	<0.001	M–W	<0.001	<0.001	M–W	<0.001	<0.001	M–W	<0.001
Zinc	<0.001	M–W	<0.001	<0.001	M–W	0.232	0.001	M–W	0.470	0.001	M–W	<0.001

^a^C1, control wetland containing floating *P. australis* in tap water at 2–day HRT

^b^T1, HC–SGW treatment systems 2-day with only floating *P. australis*

^c^T2, HC–SGW treatment systems 2-day with floating *P. australis* and ochre pellets

^d^T5, LC-SGW treatment systems 2-day with only floating *P. australis*

^e^T6, LC-SGW treatment systems 2-day with floating *P. australis* and ochre pellets

^f^Shapiro–Wilk (check for normality), normally distributed data, if *p* > 0.05 using T–test, and non–normally distributed data, if *p* < 0.05 using Mann–Whitney U–test; p value, significantly different, if p < 0.05, and not significantly different, if p > 0.05; and M–W, Mann–Whitney U–test

^g^C3, control wetland containing floating *P. australis* in tap water at 7–day HRT

^h^T9, HC-SGW treatment systems 7-day with only floating *P. australis*

^i^T10, HC-SGW treatment systems 7-day with floating *P. australis* and ochre pellets

^j^T13, LC-SGW treatment systems 7-day with only floating *P. australis*

^k^T14, LC-SGW treatment systems 7-day with floating *P. australis* and ochre pellets

In addition, elevated HRT of the purification of HC–SGW in system T10 led to a decrease in the accumulated content (Online Resource 7) of Ca and Cr in the ochre pellets of system T10 in comparison to those accumulated amounts in the pellets of system T2. Concerning the purification of LC–SGW, an elevated HRT significantly (*p* < 0.05) affected the increase in accumulated Cd and Cr in the ochre pellets of system T14 in comparison with those accumulated in the pellets of system T6 (Tables 3, 4, and Online Resource 6a–d). It is possible that ochre pellets could be fully saturated with some elements such as Cr (in system T10), which subsequently may re − dissolve [24].

When combining pellets and plants, the concentrations of B, Cd and Mg (Cr and Cu in system T2 of 2–day HRT), which accumulated within the ochre pellets of systems treating HC–SGW (T2: 2–day and T10: 7–day), were higher than those values in ochre pellets of systems T6 (2–day) and T14 (7–day), respectively, which were treating LC–SGW. However, the content of Al and Fe (T6: 2–day HRT), and Al and Ca (T14: 7–day HRT), which accumulated in the ochre pellets were higher for the cleaning of LC–SGW compared with the corresponding values in the systems T2 (2–day) and T10 (7–day), respectively, which were treating HC–SGW (Online Resource 6e and f).

When observing the concentrations of adsorbed elements, there was a significant (*p* < 0.05) correlation with the respective primary element amounts of greywater. High removals of low greywater element amounts suggest a high presence of vacant locations within ochre pellets to endure the adsorbed and absorbed elements [64].

### Assessment of trace elements accumulated in tissues of *Phragmites australis*

The averages of trace element concentrations accumulated in the tissues of *P. australis* associated with floating wetlands treating SGW (Table 3) were statistically compared to those accumulated concentrations in *P. australis* that floated in the control wetlands receiving clean tap water (TW) to investigate the potentially significant performance of each wetland (Table 4).

All considered elements had significantly (p < 0.05) elevated accumulations in *P. australis* within all purification units, except for Na that was significantly (p < 0.05) lower compared to those in the controls. However, regarding the treatment of SGW in wetlands with a combination of ochre pellets and *P. australis* at 2–day HRT, Mg accumulated in *P. australis* tissues of wetlands T2 (HC–SGW) and B, Mg and Zn accrued in wetland T6 (LC–SGW) were significantly (p < 0.05) reduced compared to those values in *P. australis* of wetland C1 (2–day; TW). For 7–day HRT, Cu, Mg, Mn and Zn concentrations accumulated in *P. australis* tissues of wetland T14 (LC–SGW) were significantly lower compared to those accumulated element concentrations in *P. australis* of the control wetland C3 (7–day; TW), as shown in Table 4, Online Resource 8a and b.

Elements such as Fe, Mn, Cu, Zn and Ni are recognised as micro−nutrients, which are necessary for physiological and biochemical processes of plant growth [65]. However, accumulations of such metals above the corresponding plant tolerance levels may cause toxicity. Toxic elements accumulate in foliage part of plants and are expelled through dead leaves [66].

The existence of both pellets and plants in wetlands treating SGW significantly affected the reduction of the concentrations of all considered trace elements, which accumulated in *P. australis* tissues with the exception of Ca that increased significantly (p < 0.05), compared to the corresponding amounts accumulated in *P. australis* tissues in systems purifying both types of SGW with only *P. australis* at both HRT (Online Resource 8a and b). However, B and Cr accumulated in *P. australis* of wetland T2 (2–day; HC–SGW; combination of ochre pellets with *P. australis*), and Na accrued in *P. australis* of wetland T14 (7–day; LC–SGW; combination of ochre pellets with *P. australis*) were significantly higher than those accumulated amounts in *P. australis* of wetlands T1 (2–day; HC–SGW: only *P. australis*) and T13 (7–day; LC–SGW; only *P. australis*), respectively (Online Resource 9). This refers to the contribution of ochre pellets in adsorption of trace elements. However, an increase in Ca concentration was linked to the presence of ochre pellets in the treatment systems [23].

Considering the effects of HRT (Online Resource 8), the concentrations of almost all elements, which accumulated in *P. australis* tissues, were significantly lower in systems purifying both categories of SGW for 7–day HRT in comparison to those amounts accumulated in *P. australis* of systems at HRT of 2 days. This was inverse to the behaviour exhibited in the control wetlands, when comparing the trace element accumulations in *P. australis* of 7–day (C3) with those of 2–day HRT (C1). However, remarkable increases were observed with increasing HRT in Fe, Mg and Ni accumulation in *P. australis* of wetland T10 (HC–SGW; combination of ochre pellets with *P. australis*), and Al, Ca and Na in wetland T14 (LC–SGW; combination of ochre pellets with *P. australis*) in comparison to those concentrations at 2 days of treatment within wetlands T2 (HC–SGW) and T6 (LC–SGW).

The take–up of metals in the aquatic environment is influenced by interactions of many factors such as diurnal and long−term changes in pH and the bioavailability of metals [67]. Phytoremediation of metals is a function of activities within the rhizosphere under acidification or alkalinisation conditions, which are affected by many physicochemical and biological properties of water and plant species [68].

Accumulation rates in tissues of *P. australis* were higher for all considered trace elements in systems purifying HC–SGW in comparison to those values in systems treating LC–SGW, except for the Ni accumulation rate, which was higher in wetland T5 treating LC–SGW (Online Resource 8c and d). In this context, it has been reported that FTW are capable of coping with contaminated wastewater with high pollutant variations [35], especially at the existence of pellets, which have been proven as a good adsorbent of trace elements [24, 63, 64].

This study shows that floating *P. australis* operated in hydroponic manner have significant (*p* < 0.05) tolerance for remediation of the elevated concentrations of pollutants such as heavy elements. However, they have a negative impact on the development rates of floating treatment wetlands.

The distribution of trace elements within *P. australis* roots, rhizomes, stems and leaves was investigated (Online Resource 10). The comparative study showed that trace elements accumulated in roots and rhizomes were significantly (*p* < 0.05) elevated than those concentrations in stems and leaves (Online Resource 8) [69].

The accumulations of trace elements varied concerning different plant parts. Concentrations within leaves were commonly higher compared to those within stems. The capacity to uptake elements and the intracellular transportation processes vary between different macrophytes [70]. An exception to this is Zn, which was present in elevated concentrations in stems (Online Resource 10), because of the presence of growth hormones [67].

In comparison with control wetlands, the accumulated elements in plant rhizomes grown within all treatment systems were significantly (p < 0.05) elevated in terms of Al, B, Ca, Cd, Cr, Cu, Fe, Mg, Mn and Ni. However, the concentrations of Na and Zn were lower than those concentrations of control wetlands (Online Resource 8a and b). The concentrations of almost all trace elements in stems and leaves were significantly (p < 0.05) elevated in treatment systems in comparison to control wetlands. However, accumulations of Cr, Mg, Na and Ni were lower in stems of *P. australis* in almost all treatment wetlands.

Cr is usually weakly soluble and absorbed by plants. Zn, Cu and Ni are elements that are smoothly absorbed and transported to plant shoots. Finally, Cd has serious negative effects on the plant food chain [71].

Element accumulation in floating *P. australis* of systems treating HC–SGW were significantly (p < 0.05) greater than those values linked to the treatment of LC–SGW, through different locations of the plants, as shown by comparing Online Resource 8a and b with Online Resource 8c and d, respectively.

The existence of pellets within the systems had a significant (p < 0.05) effect on the decrease in the build−up of elements in rhizomes except for Ca, which had significantly (p < 0.05) greater accumulations in *P. australis* rhizomes when ochre−based pellets were available.

Elevated purification HRT using a combination of ochre pellets and *P. australis* significantly (p < 0.05) rose the amount of accumulated trace elements in rhizomes of *P. australis* in comparison with corresponding values in systems that had only a HRT of 2 days. In contrast, the accumulation in rhizomes of systems comprising only floating macrophytes were significantly (p < 0.05) elevated in systems of 2–day purification time compared with systems of 7–day HRT, as shown by comparing Online Resource 8a and c with Online Resource 8b and d, respectively.

Although it has been reported that concentrations of metals in below−ground plant tissues are higher than in plant parts located aboveground, it is necessary to consider the biomass of each plant part to evaluate the total accumulation amount [71]. Translocation of accumulated metals in plant tissues could be affected by the interference of several factors such as water pH, microorganism activities as well as plant species and their enzymes [66]. High concentrations of toxic elements can be transferred to the stems and leaves of wetland macrophytes causing a reduction of above−ground biomass growth. Effective phytoremediation correlates positively with plant biomass [67].

## Conclusions and recommendations

The performance of floating treatment wetlands can be improved by utilising ochre–cement pellets to increase the pH of greywater and subsequently increase the EC through releasing Ca coupled with Al and Fe, supporting coagulation and flocculation. However, the presence of *P. australis* acts as a buffer to neutralise the pH of SGWs by producing carbon dioxide through rhizome action and organic acids, which are by–products of biodegradation processes of organic substances. Furthermore, rhizomes and biofilms attached to *P. australis* mitigate increases in turbidity, TSS and colour values, which occur by using ochre pellets.

Ochre pellets in combination with *P. australis* have shown significant removals of NH_4_–N and PO_4_–P due to nitrification and adsorption mechanisms, respectively, especially with increasing HRT. Due to the presence of ochre pellets, concentrations of Al, Ca and Fe significantly increased in the effluent of treated SGW. A significant improvement in reductions of B, Ca, Cd, Cr, Cu, Fe, Mn, Ni and Zn concentrations in the treatment of SGW was recorded at the presence of *P. australis* in combination with ochre pellets. However, concentrations of Al, B, and Ni significantly dropped in treated SGW with an increase of the HRT. However, a significant rise in concentrations of Ca, Fe and Mg was noted.

Floating treatment wetlands have shown significant performance in removal of Al, Ca, Fe and Na from high pollutant strength greywater. Significant removal of Cd, Cu, Mg, Mn, Ni and Zn was recorded from greywater of low pollutant strength.

The competition between ochre pellets and *P. australis* in remediation of heavy metals and other elements from the SGW has led to significant reductions in the concentrations of accumulated elements in the tissues of *P. australis*, except for Ca. Higher accumulations of elements (except for Ni) in tissues of plants, which treated high pollutant strength SGW, was noted. Furthermore, considerable concentrations of accumulated elements have been found in *P. australis* tissues of FTW purifying SGW for 2 days in contrast of control wetlands receiving tap water. Trace element accumulations in rhizomes were significantly higher compared to those in stems and leaves. Al, B, Ca, Cd, Cr, Cu, Fe and Zn have significantly increased in ochre pellets at the presence of *P. australis*.

Further research on large–scale floating wetland systems is recommended. Moreover, biochemical and ecological investigations would help to better understand the biological processes.

## Electronic supplementary material

Online resource 1 (DOCX 16.8 kb)

Online resource 2 (DOCX 18 kb)

Online resource 3 (DOCX 66.1 kb)

Online resource 4 (DOCX 34 kb)

Online resource 5 (DOCX 15.5 kb)

Online resource 6 (DOCX 107 kb)

Online resource 7 (DOCX 22.6 kb)

Online resource 8 (DOCX 87.2 kb)

Online resource 9 (DOCX 21.6 kb)

Online resource 10 (DOCX 23.8 kb)

## Data Availability

All data generated or analysed during this study are included in this published article.
