# Supplementary material for: Phytoremediation performance of floating treatment wetlands with pelletized mine water sludge for synthetic greywater treatment
Source: J Environ Health Sci Eng. 2019 Apr 18;17(2):581–608. doi: 10.1007/s40201-019-00372-z (PMC6985343; doi:10.1007/s40201-019-00372-z)
Supplement: Supplementary file 1 — (DOCX 16.8 kb) [file 40201_2019_372_MOESM1_ESM.docx]

**Phytoremediation performance of floating treatment wetlands with pelletized mine water sludge for synthetic greywater treatment**

*Journal of Environmental Health Science and Engineering*

**Suhail N. Abed, Suhad A. Almuktar, Miklas Scholz**

Corresponding author: Miklas Scholz

Civil Engineering Research Group, School of Computing, Science and Engineering, The University of Salford, Newton Building, Salford M5 4WT, England, United Kingdom.

Division of Water Resources Engineering, Department of Building and Environmental Technology, Faculty of Engineering, Lund University, P.O. Box 118, 221 00 Lund, Sweden.

Department of Civil Engineering Science, School of Civil Engineering and the Built Environment, University of Johannesburg, Kingsway Campus, PO Box 524, Aukland Park 2006, Johannesburg, South Africa

E‒mail address: miklas.scholz@tvrl.lth.se

**Online Resource 1** Ingredients for low (LC) and high (HC) pollutant concentrations of synthetic greywaters (SGW) adopted from Abed and Scholz (2016)

| Item | Chemical name | Chemical formula* | Molar mass (g/mol) | SGW recipe (mg/L) | | Composition percentages* |
| --- | --- | --- | --- | --- | --- | --- |
|  |  |  |  | LC | HC |  |
| 1 | Kaolin | Al_2_Si_2_O_5_(OH)_4_ | 258.16 | 15 | 100 | Al (20.90%), H (1.56%), O (55.78%) and Si (21.76%) |
| 2 | Cellulose | (C_6_H_10_O_5_) _n_ | 162.14 | 15 | 100 | C (44.45%), H (6.22%) and O (49.34%) |
| 3 | Humic acid | C_187_H_186_O_89_N_9_S_1_ | 4015.55 | 5 | 20 | C (55.90%), H (4.67%), O (35.46%), N (4.67%) and S (0.80%) |
| 4 | Sodium chloride | NaCl | 58.44 | 10 | 120 | Cl (60.66%) and Na (39.34%) |
| 5 | Sodium hydrogen carbonate | NaHCO_3_ | 84.01 | 10 | 85 | C (14.30%), H (1.20%), Na (27.37%) and O (57.14%) |
| 6 | Calcium chloride | CaCl_2_ | 147.02 | 10 | 55 | Ca (36.11%) and Cl (63.89%) |
| 7 | Potassium nitrate | KNO_3_ | 101.10 | 0 | 90 | K (38.67%), N (13.85%) and O (47.48%) |
| 8 | Calcium nitrate | Ca(NO_3_)_2_ | 164.09 | 0 | 150 | Ca (24.43%), N (17.07%) and O (58.50%) |
| 9 | Magnesium sulphate | MgSO_4_ | 120.37 | 2 | 240 | Mg (20.19%), S (26.64%) and O (53.17%) |
| 10 | Monopotassium phosphate | KH_2_PO_4_ | 136.09 | 13 | 85 | H (1.48%), K (28.73%), O (47.03%) and P (22.76%) |
| 11 | Iron(III)chloride | FeCl_3_ | 162.20 | 0.3 | 50.0 | Fe (34.43%) and Cl (65.57%) |
| 12 | Boric acid | H_3_BO_3_ | 61.83 | 0.6 | 3.0 | H (4.89%), B (17.48%) and O (77.63%) |
| 13 | Manganese(II)chloride | MnCl_2_ | 125.84 | 0.03 | 3.20 | Cl (56.34%) and Mn (43.66%) |
| 14 | Zinc sulphate | ZnSO_4_ | 161.44 | 0.25 | 15.00 | O (39.64%), S (19.86%) and Zn (40.50%) |
| 15 | Copper sulphate | CuSO_4_ | 159.61 | 0.025 | 7.00 | Cu (39.81%), O (40.10%) and S (20.09%) |
| 16 | Ammonium molybdate tetrahydrate | (NH_4_)_6_Mo_7_O_24_ | 1163.94 | 0.35 | 0.35 | H (2.08%), Mo (57.71%), N (7.22%) and O (32.99%) |
| 17 | Cadmium oxide | CdO | 128.41 | 0.02 | 12.50 | Cd (87.54%) and O (12.46%) |
| 18 | Nickel oxide | NiO | 74.69 | 0.02 | 0.06 | Ni (78.58%) and O (21.42%) |
| 19 | Chromium(III)nitrate | CrN_3_O_9_ | 99.99 | 0.045 | 70.00 | Cr (21.85%), N (17.65%) and O (60.50%) |
| 20 | Sodium sulphate | Na_2_SO_4_ | 142.04 | 2.60 | 25.00 | Na (32.37%), O (45.06%) and S (22.57%) |
| 21 | Sodium phosphate monobasic | H_2_NaPO_4_ | 119.98 | 0.00 | 250.00 | H (1.68%), Na (19.16%), O (53.34%) and P (25.82%) |
| 22 | Lead(II)oxide | Pb_3_O_4_ | 685.60 | 0.16 | 1.40 | Pb (90.67%) and O (9.33%) |

* Al, aluminium; H, hydrogen; O, oxygen; Si, silicon; C, carbon; N, nitrogen; S, sulphur; Cl, chlorine; Na, sodium; Ca, calcium; K, potassium; Mg, magnesium; P, phosphorus; Fe, iron; B, boron; Mn, manganese; Zn, zinc; Cu, copper; Mo, molybdenum; Cd, Cadmium; Ni, nickel; Cr, chromium; and Pb, lead
