# Supplementary material for: Phytoremediation performance of floating treatment wetlands with pelletized mine water sludge for synthetic greywater treatment
Source: J Environ Health Sci Eng. 2019 Apr 18;17(2):581–608. doi: 10.1007/s40201-019-00372-z (PMC6985343; doi:10.1007/s40201-019-00372-z)
Supplement: Supplementary file 3 — (DOCX 66.1 kb) [file 40201_2019_372_MOESM3_ESM.docx]

**Phytoremediation performance of floating treatment wetlands with pelletized mine water sludge for synthetic greywater treatment**

*Journal of Environmental Health Science and Engineering*

**Suhail N. Abed, Suhad A. Almuktar, Miklas Scholz**

Corresponding author: Miklas Scholz

Civil Engineering Research Group, School of Computing, Science and Engineering, The University of Salford, Newton Building, Salford M5 4WT, England, United Kingdom.

Division of Water Resources Engineering, Department of Building and Environmental Technology, Faculty of Engineering, Lund University, P.O. Box 118, 221 00 Lund, Sweden.

Department of Civil Engineering Science, School of Civil Engineering and the Built Environment, University of Johannesburg, Kingsway Campus, PO Box 524, Aukland Park 2006, Johannesburg, South Africa

E‒mail address: miklas.scholz@tvrl.lth.se

**Online Resource 3** Outflow quality of the synthetic greywater (SGW) treated at 2‒ and 7‒ day HRT for *(a)* high concentration (HC‒SGW) at 2‒day HRT, *(b)* low concentration (LC‒SGW) at 2‒day HRT, *(c)* HC‒SGW at 7‒day HRT, *d)* LC‒SGW at 7‒day HRT, and *e)* outflow of control wetlands which received tap water (TW)

| a) HC‒SGW (2‒day HRT^a^) | | | | | | | | | | | | | | |
| --- | --- | --- | --- | --- | --- | --- | --- | --- | --- | --- | --- | --- | --- | --- |
|  |  | 2‒day outflow HC T1^b^ | | | | | | 2‒day outflow HC T2^c^ | | | | | | |
| Parameter | Unit | n^d^ | Mean | SD^e^ | Min^f^ | Max^g^ | Rem^h^ (%) | n^d^ | Mean | SD^e^ | Min^f^ | Max^g^ | | Rem^h^ (%) |
| pH | ‒ | 85 | 7.4 | 1.09 | 5.7 | 9.5 | na^i^ | 36 | 8.8 | 1.69 | 6.6 | 12.5 | | na^i^ |
| Redox potential | mV | 85 | 8.1 | 52.68 | -89.5 | 87.2 | na^i^ | 36 | -54.8 | 83.66 | -227.7 | 56.7 | | na^i^ |
| Turbidity | NTU^j^ | 85 | 175.9 | 59.61 | 39.8 | 319.0 | 6.9 | 36 | 223.8 | 97.40 | 88.9 | 434.0 | | -18.5 |
| Total suspended solids | mg/L | 85 | 302.9 | 75.19 | 79.0 | 455.0 | 4.4 | 36 | 422.5 | 152.77 | 178.0 | 662.0 | | -33.3 |
| Electronic conductivity | µS/cm | 85 | 987.4 | 107.25 | 713.0 | 1225.0 | na^i^ | 36 | 1174.5 | 282.81 | 766.0 | 1924.0 | | na^i^ |
| Dissolved oxygen | mg/L | 85 | 9.0 | 1.03 | 5.5 | 11.9 | 14.3 | 36 | 9.0 | 1.24 | 6.5 | 12.2 | | 14.3 |
| Colour | Pa/Co | 85 | 1525.6 | 411.54 | 455.0 | 2361.0 | 3.9 | 36 | 2150.8 | 864.04 | 784.0 | 4175.0 | | -35.5 |
| Temperature | °C | 85 | 17.1 | 4.92 | 5.8 | 24.6 | na^i^ | 36 | 17.4 | 4.87 | 6.0 | 23.8 | | na^i^ |
| Biochemical oxygen demand | mg/L | 85 | 17.7 | 6.40 | 0.0 | 30.0 | 49.0 | 36 | 11.1 | 5.89 | 0.0 | 28.0 | | 68.0 |
| Chemical oxygen demand | mg/L | 85 | 96.3 | 32.01 | 33.8 | 172.0 | 25.5 | 36 | 109.2 | 24.38 | 56.9 | 151.0 | | 15.5 |
| Ammonia‒nitrogen | mg/L | 85 | 0.4 | 0.21 | 0.0 | 1.2 | 0.0 | 36 | 0.4 | 0.13 | 0.2 | 0.7 | | 0.0 |
| Nitrate‒nitrogen | mg/L | 85 | 14.1 | 6.40 | 0.2 | 32.0 | -58.4 | 36 | 14.3 | 5.02 | 4.7 | 26.5 | | -60.7 |
| Ortho‒phosphate‒phosphorus | mg/L | 85 | 52.0 | 14.87 | 15.8 | 95.5 | 12.0 | 36 | 21.1 | 5.81 | 6.9 | 29.2 | | 64.3 |
| Element | | | | | | | | | | | | | | |
| Aluminium | mg/L | 39 | 1.54 | 1.479 | 0.30 | 5.04 | 27.70 | 39 | 2.02 | 1.624 | 0.48 | 4.94 | 5.16 | |
| Boron | mg/L | 35 | 0.53 | 0.086 | 0.43 | 0.73 | 7.02 | 35 | 0.41 | 0.079 | 0.34 | 0.57 | 28.07 | |
| Calcium | mg/L | 37 | 42.50 | 4.561 | 34.22 | 49.76 | -17.79 | 37 | 81.39 | 23.641 | 46.90 | 115.66 | -125.58 | |
| Cadmium | mg/L | 42 | 4.90 | 2.730 | 1.55 | 8.93 | 33.42 | 42 | 4.10 | 1.839 | 1.79 | 7.25 | 44.36 | |
| Chromium | mg/L | 58 | 2.48 | 2.060 | 0.59 | 6.69 | 22.50 | 58 | 2.74 | 2.021 | 0.83 | 5.85 | 14.38 | |
| Copper | mg/L | 63 | 0.95 | 0.561 | 0.29 | 1.57 | 34.03 | 63 | 0.90 | 0.375 | 0.44 | 1.36 | 37.50 | |
| Iron | mg/L | 51 | 4.31 | 2.928 | 1.08 | 9.02 | 32.76 | 51 | 4.71 | 2.744 | 1.37 | 8.10 | 26.52 | |
| Potassium | mg/L | 14 | 52.79 | 1.322 | 50.41 | 54.90 | 12.25 | 14 | 54.03 | 11.214 | 44.74 | 71.52 | 10.19 | |
| Magnesium | mg/L | 48 | 17.32 | 1.296 | 14.49 | 20.30 | -0.93 | 48 | 11.01 | 2.533 | 7.57 | 14.16 | 35.84 | |
| Manganese | mg/L | 63 | 0.48 | 0.320 | 0.10 | 0.91 | 51.02 | 63 | 0.51 | 0.255 | 0.29 | 0.97 | 47.96 | |
| Sodium | mg/L | 14 | 58.54 | 11.080 | 42.44 | 73.63 | 6.60 | 14 | 56.95 | 9.494 | 42.01 | 67.61 | 6.18 | |
| Nickel | mg/L | 53 | 0.02 | 0.019 | 0.00 | 0.06 | 60.00 | 53 | 0.02 | 0.019 | 0.00 | 0.08 | 60.00 | |
| Zinc | mg/L | 44 | 2.86 | 1.680 | 0.70 | 4.70 | 32.71 | 44 | 2.58 | 1.114 | 1.01 | 4.18 | 39.29 | |
|  |  | 2‒day outflow HC T3^k^ | | | | | | 2‒day outflow HC T4^l^ | | | | | | |
| pH | ‒ | 85 | 7.8 | 1.37 | 5.6 | 9.8 | na^i^ | 36 | 8.7 | 1.73 | 6.6 | 12.7 | na^i^ | |
| Redox potential | mV | 85 | -3.0 | 62.95 | -107.6 | 88.6 | na^i^ | 36 | -49.9 | 83.61 | -232.8 | 42.1 | na^i^ | |
| Turbidity | NTU^j^ | 85 | 192.1 | 50.87 | 102.0 | 341.0 | -1.7 | 36 | 191.3 | 84.41 | 106.0 | 456.0 | -1.3 | |
| Total suspended solids | mg/L | 85 | 321.8 | 56.68 | 165.0 | 447.0 | -1.5 | 36 | 337.4 | 109.45 | 161.0 | 661.0 | -6.4 | |
| Electronic conductivity | µS/cm | 85 | 965.2 | 106.68 | 627.0 | 1208.0 | na^i^ | 36 | 1178.4 | 264.41 | 806.0 | 1944.0 | na^i^ | |
| Dissolved oxygen | mg/L | 85 | 10.2 | 0.73 | 8.7 | 12.1 | 2.9 | 36 | 10.0 | 0.52 | 8.9 | 10.9 | 4.8 | |
| Colour | Pa/Co | 85 | 1527.6 | 326.28 | 677.0 | 2311.0 | 3.8 | 36 | 1935.6 | 702.18 | 702.0 | 3438.0 | -21.9 | |
| Temperature | °C | 85 | 17.1 | 4.75 | 6.1 | 23.6 | na^i^ | 36 | 17.2 | 4.73 | 6.0 | 23.4 | na^i^ | |
| Biochemical oxygen demand | mg/L | 85 | 14.7 | 7.78 | 0.0 | 40.0 | 57.6 | 36 | 11.7 | 7.71 | 0.0 | 35.0 | 66.3 | |
| Chemical oxygen demand | mg/L | 85 | 106.6 | 22.68 | 43.3 | 164.0 | 17.5 | 36 | 100.3 | 21.08 | 41.7 | 131.0 | 22.4 | |
| Ammonia‒nitrogen | mg/L | 85 | 0.4 | 0.16 | 0.0 | 0.9 | 0.0 | 36 | 0.4 | 0.09 | 0.2 | 0.6 | 0.0 | |
| Nitrate‒nitrogen | mg/L | 85 | 9.4 | 4.67 | 0.5 | 24.0 | -5.6 | 36 | 12.9 | 7.03 | 2.4 | 26.9 | -44.9 | |
| Ortho‒phosphate‒phosphorus | mg/L | 85 | 46.2 | 10.74 | 23.7 | 70.1 | 21.8 | 36 | 19.5 | 4.98 | 9.0 | 30.3 | 67.0 | |
| Element | | | | | | | | | | | | | | |
| Aluminium | mg/L | 39 | 2.41 | 1.016 | 0.74 | 4.25 | -13.15 | 39 | 2.98 | 2.087 | 0.84 | 7.14 | -39.91 | |
| Boron | mg/L | 35 | 0.54 | 0.060 | 0.42 | 0.66 | 5.26 | 35 | 0.50 | 0.078 | 0.40 | 0.68 | 12.28 | |
| Calcium | mg/L | 37 | 43.02 | 2.411 | 35.94 | 46.28 | -19.24 | 37 | 104.13 | 32.868 | 46.28 | 141.65 | -188.61 | |
| Cadmium | mg/L | 42 | 7.69 | 1.064 | 4.95 | 8.98 | -4.48 | 42 | 7.14 | 2.429 | 4.23 | 11.75 | 2.99 | |
| Chromium | mg/L | 58 | 3.76 | 1.203 | 1.34 | 4.98 | -17.5 | 58 | 3.99 | 1.806 | 1.87 | 7.03 | -24.69 | |
| Copper | mg/L | 63 | 1.45 | 0.113 | 1.28 | 1.70 | -0.69 | 63 | 1.55 | 0.308 | 1.23 | 2.09 | -7.64 | |
| Iron | mg/L | 51 | 6.35 | 2.423 | 1.56 | 9.29 | 0.94 | 51 | 7.11 | 2.934 | 2.60 | 11.55 | -10.92 | |
| Potassium | mg/L | 14 | 55.68 | 4.486 | 49.48 | 60.69 | 7.45 | 14 | 60.47 | 15.561 | 49.85 | 85.46 | -0.52 | |
| Magnesium | mg/L | 48 | 17.76 | 1.392 | 13.92 | 19.55 | -0.20 | 48 | 13.33 | 4.526 | 7.53 | 19.80 | 22.32 | |
| Manganese | mg/L | 63 | 1.19 | 0.063 | 1.06 | 1.29 | -21.4 | 63 | 0.89 | 0.396 | 0.61 | 1.59 | 9.18 | |
| Sodium | mg/L | 14 | 58.19 | 10.620 | 42.35 | 68.22 | 7.16 | 14 | 58.54 | 11.630 | 41.37 | 76.33 | 6.60 | |
| Nickel | mg/L | 53 | 0.03 | 0.018 | 0.00 | 0.06 | 40.00 | 53 | 0.03 | 0.033 | 0.00 | 0.10 | 40.00 | |
| Zinc | mg/L | 44 | 4.30 | 0.524 | 3.12 | 5.25 | -1.42 | 44 | 4.52 | 0.961 | 2.90 | 6.40 | -6.35 | |

^a^ HRT, hydraulic retention time

^b^ T1, treatment system with only *Phragmites australis*

^c^ T2, treatment system with *P. australis* and ochre pellets;

^d^ n, number of tested samples

^e^ SD, standard deviation

^f^ Min, minimum

^g^ Max, maximum

^h^ Rem, removal

^i^ na, not applicable

^j^ NTU, nephelometric turbidity unit

^k^ T3, treatment system without *P. australis* or ochre pellets

^l^ T4, treatment system with only ochre pellets

**Online Resource 3** (Continued)

| b) LC‒SGW (2‒day HRT^a^) | | | | | | | | | | | | | | |
| --- | --- | --- | --- | --- | --- | --- | --- | --- | --- | --- | --- | --- | --- | --- |
|  |  | 2‒day outflow LC T5^b^ | | | | | | 2‒day outflow LC T6^c^ | | | | | | |
| Parameter | Unit | n^d^ | Mean | SD^e^ | Min^f^ | Max^g^ | Rem^h^ (%) | n^d^ | Mean | SD^e^ | Min^f^ | Max^g^ | | Rem^h^ (%) |
| pH | ‒ | 85 | 7.0 | 0.71 | 6.1 | 10.2 | na^i^ | 36 | 10.5 | 1.12 | 8.0 | 12.5 | | na^i^ |
| Redox potential | mV | 85 | 27.5 | 32.18 | -120.8 | 72.4 | na^i^ | 36 | -137.4 | 54.91 | -224.8 | -15.0 | | na^i^ |
| Turbidity | NTU^j^ | 85 | 28.2 | 37.09 | 3.4 | 262.0 | -23.1 | 36 | 39.2 | 45.10 | 9.8 | 260.0 | | -71.2 |
| Total suspended solids | mg/L | 85 | 41.7 | 43.57 | 8.0 | 316.0 | -4.5 | 36 | 62.0 | 49.93 | 13.0 | 262.0 | | -55.4 |
| Electronic conductivity | µS/cm | 85 | 145.9 | 30.41 | 15.5 | 325.0 | na^i^ | 36 | 371.5 | 260.12 | 4.1 | 1307.0 | | na^i^ |
| Dissolved oxygen | mg/L | 85 | 9.3 | 1.08 | 6.9 | 12.5 | 10.6 | 36 | 8.8 | 0.87 | 6.5 | 10.6 | | 15.4 |
| Colour | Pa/Co | 85 | 183.7 | 74.89 | 73.0 | 476.0 | 14.4 | 36 | 308.2 | 134.65 | 103.0 | 683.0 | | -43.7 |
| Temperature | °C | 85 | 17.0 | 4.84 | 6.0 | 23.0 | na^i^ | 36 | 16.6 | 4.55 | 6.2 | 22.9 | | na^i^ |
| Biochemical oxygen demand | mg/L | 85 | 9.9 | 5.49 | 0.0 | 30.0 | 43.8 | 36 | 5.4 | 4.36 | 0.0 | 18.0 | | 69.3 |
| Chemical oxygen demand | mg/L | 85 | 32.4 | 14.55 | 10.6 | 89.6 | -13.8 | 36 | 29.6 | 16.67 | 9.6 | 73.4 | | -2.4 |
| Ammonia‒nitrogen | mg/L | 85 | 0.1 | 0.07 | 0.0 | 0.3 | 50.0 | 36 | 0.2 | 0.14 | 0.0 | 0.5 | | 0.0 |
| Nitrate‒nitrogen | mg/L | 85 | 1.7 | 1.13 | 0.0 | 5.8 | -30.8 | 36 | 0.4 | 0.33 | 0.0 | 1.6 | | 69.2 |
| Ortho‒phosphate‒phosphorus | mg/L | 85 | 7.6 | 3.90 | 3.2 | 120 | 9.5 | 36 | 3.2 | 1.16 | 1.5 | 6.4 | | 86.2 |
| Element | | | | | | | | | | | | | | |
| Aluminium | mg/L | 39 | 0.08 | 0.054 | 0.01 | 0.19 | 84.62 | 39 | 1.07 | 0.874 | 0.08 | 2.54 | -105.77 | |
| Boron | mg/L | 35 | 0.11 | 0.010 | 0.10 | 0.14 | 21.43 | 35 | 0.09 | 0.011 | 0.08 | 0.11 | 35.71 | |
| Calcium | mg/L | 46 | 11.51 | 0.926 | 10.01 | 13.18 | -9.20 | 37 | 45.13 | 11.676 | 27.19 | 63.22 | -332.07 | |
| Cadmium | mg/L | 42 | 0.04 | 0.020 | 0.00 | 0.08 | 55.56 | 42 | 0.03 | 0.019 | 0.00 | 0.07 | 66.67 | |
| Chromium | mg/L | 58 | 0.03 | 0.036 | 0.00 | 0.10 | 25.00 | 58 | 0.03 | 0.033 | 0.00 | 0.09 | 25.00 | |
| Copper | mg/L | 63 | 0.04 | 0.029 | 0.01 | 0.09 | 75.00 | 63 | 0.04 | 0.035 | 0.00 | 0.10 | 75.00 | |
| Iron | mg/L | 51 | 0.15 | 0.118 | 0.04 | 0.33 | 28.57 | 51 | 0.21 | 0.202 | 0.05 | 0.54 | 0.00 | |
| Potassium | mg/L | 14 | 3.40 | 0.675 | 2.50 | 4.36 | 15.84 | 14 | 10.78 | 10.185 | 2.97 | 27.17 | -166.83 | |
| Magnesium | mg/L | 48 | 1.36 | 0.157 | 1.05 | 1.62 | 6.21 | 48 | 0.63 | 0.310 | 0.23 | 0.95 | 56.55 | |
| Manganese | mg/L | 63 | 0.01 | 0.012 | 0.00 | 0.06 | 94.12 | 63 | 0.04 | 0.031 | 0.00 | 0.11 | 76.47 | |
| Sodium | mg/L | 14 | 14.74 | 1.282 | 13.12 | 18.08 | -2.93 | 14 | 15.90 | 1.869 | 13.87 | 18.80 | -11.03 | |
| Nickel | mg/L | 53 | 0.004 | 0.006 | 0.00 | 0.04 | 90.00 | 53 | 0.01 | 0.010 | 0.00 | 0.06 | 75.00 | |
| Zinc | mg/L | 42 | 0.06 | 0.066 | 0.00 | 0.17 | 71.43 | 42 | 0.04 | 0.054 | 0.00 | 0.17 | 80.92 | |
|  |  | 2‒day outflow LC T7^k^ | | | | | | 2‒day outflow LC T8^l^ | | | | | | |
| pH | ‒ | 85 | 7.5 | 0.70 | 6.3 | 10.1 | na^i^ | 36 | 10.6 | 0.99 | 8.5 | 12.5 | na^i^ | |
| Redox potential | mV | 85 | 4.2 | 30.40 | -116.1 | 51.0 | na^i^ | 36 | -143.5 | 51.01 | -238.1 | -30.3 | na^i^ | |
| Turbidity | NTU^j^ | 85 | 20.2 | 14.20 | 2.9 | 129.0 | 11.8 | 36 | 35.6 | 18.11 | 8.7 | 79.3 | -55.5 | |
| Total suspended solids | mg/L | 85 | 30.0 | 12.12 | 11.0 | 76.0 | 24.8 | 36 | 66.2 | 36.63 | 13.0 | 181.0 | -65.9 | |
| Electronic conductivity | µS/cm | 85 | 138.5 | 23.26 | 79.0 | 215.0 | na^i^ | 36 | 344.5 | 287.03 | 168.4 | 1534.0 | na^i^ | |
| Dissolved oxygen | mg/L | 85 | 10.5 | 0.82 | 8.2 | 12.6 | -1.0 | 36 | 10.1 | 0.73 | 6.5 | 10.8 | 2.9 | |
| Colour | Pa/Co | 85 | 164.5 | 40.93 | 34.0 | 265.0 | 23.3 | 36 | 331.7 | 119.34 | 104.0 | 552.0 | -54.6 | |
| Temperature | °C | 85 | 16.0 | 4.59 | 5.3 | 21.8 | na^i^ | 36 | 16.3 | 4.24 | 6.3 | 21.3 | na^i^ | |
| Biochemical oxygen demand | mg/L | 85 | 5.6 | 3.60 | 0.0 | 20.0 | 68.2 | 36 | 4.4 | 5.13 | 0.0 | 22.0 | 75.0 | |
| Chemical oxygen demand | mg/L | 85 | 26.8 | 6.18 | 15.4 | 41.9 | 7.3 | 36 | 24.0 | 4.99 | 15.4 | 39.9 | 17.0 | |
| Ammonia‒nitrogen | mg/L | 85 | 0.09 | 0.05 | 0.0 | 0.3 | 55.0 | 36 | 0.1 | 0.04 | 0.1 | 0.2 | 50.0 | |
| Nitrate‒nitrogen | mg/L | 85 | 1.2 | 0.71 | 0.1 | 3.2 | 7.7 | 36 | 0.6 | 0.54 | 0.0 | 2.6 | 53.8 | |
| Ortho‒phosphate‒phosphorus | mg/L | 85 | 7.0 | 3.89 | 3.0 | 18.8 | 16.7 | 36 | 3.9 | 1.25 | 2.2 | 7.1 | 53.6 | |
| Element | | | | | | | | | | | | | | |
| Aluminium | mg/L | 39 | 0.34 | 0.180 | 0.11 | 0.72 | 34.62 | 39 | 0.76 | 0.347 | 0.16 | 1.24 | -46.15 | |
| Boron | mg/L | 35 | 0.11 | 0.009 | 0.08 | 0.13 | 21.43 | 35 | 0.10 | 0.024 | 0.07 | 0.13 | 28.57 | |
| Calcium | mg/L | 46 | 11.25 | 0.773 | 9.86 | 12.70 | -6.74 | 37 | 70.99 | 33.166 | 21.66 | 109.98 | -573.53 | |
| Cadmium | mg/L | 42 | 0.05 | 0.031 | 0.00 | 0.11 | 44.44 | 42 | 0.04 | 0.030 | 0.00 | 0.10 | 55.56 | |
| Chromium | mg/L | 58 | 0.04 | 0.049 | 0.00 | 0.12 | 0.00 | 58 | 0.05 | 0.039 | 0.00 | 0.12 | -25.00 | |
| Copper | mg/L | 63 | 0.06 | 0.049 | 0.02 | 0.15 | 62.50 | 63 | 0.05 | 0.043 | 0.01 | 0.13 | 68.75 | |
| Iron | mg/L | 51 | 0.21 | 0.157 | 0.09 | 0.45 | 0.00 | 51 | 0.48 | 0.447 | 0.15 | 1.26 | -128.57 | |
| Potassium | mg/L | 14 | 3.87 | 0.364 | 3.35 | 4.50 | 4.21 | 14 | 12.77 | 15.139 | 2.73 | 36.71 | -216.09 | |
| Magnesium | mg/L | 48 | 1.35 | 0.133 | 0.99 | 1.58 | 6.90 | 48 | 0.70 | 0.336 | 0.28 | 1.15 | 51.72 | |
| Manganese | mg/L | 63 | 0.08 | 0.056 | 0.00 | 0.18 | 52.94 | 63 | 0.08 | 0.069 | 0.00 | 0.20 | 52.94 | |
| Sodium | mg/L | 14 | 13.82 | 1.175 | 12.14 | 15.57 | 3.49 | 14 | 15.35 | 3.197 | 12.32 | 20.34 | -7.19 | |
| Nickel | mg/L | 53 | 0.01 | 0.007 | 0.00 | 0.04 | 75.00 | 53 | 0.01 | 0.012 | 0.00 | 0.06 | 75.00 | |
| Zinc | mg/L | 42 | 0.09 | 0.083 | 0.00 | 0.23 | 57.14 | 42 | 0.07 | 0.084 | 0.00 | 0.29 | 66.67 | |

^a^ HRT, hydraulic retention time

^b^ T5, treatment system with only *Phragmites australis*

^c^ T6, treatment system with *P. australis* and ochre pellets

^d^ n, number of tested samples

^e^ SD, standard deviation

^f^ Min, minimum

^g^ Max, maximum

^h^ Rem, removal

^i^ na, not applicable

^j^ NTU, nephelometric turbidity unit

^k^ T7, treatment system without *P. australis* or ochre pellets

^l^ T8, treatment system with only ochre pellets

**Online Resource 3** (Continued)

| c) HC‒SGW (7‒day HRT^a^) | | | | | | | | | | | | | | |
| --- | --- | --- | --- | --- | --- | --- | --- | --- | --- | --- | --- | --- | --- | --- |
|  |  | 7‒day outflow HC T9^b^ | | | | | | 7‒day outflow HC T10^c^ | | | | | | |
| Parameter | Unit | n^d^ | Mean | SD^e^ | Min^f^ | Max^g^ | Rem^h^ (%) | n^d^ | Mean | SD^e^ | Min^f^ | Max^g^ | | Rem^h^ (%) |
| pH | ‒ | 83 | 7.3 | 0.82 | 5.9 | 8.8 | na^i^ | 34 | 9.8 | 1.34 | 7.3 | 12.3 | | na^i^ |
| Redox potential | mV | 83 | 12.2 | 40.30 | -57.6 | 77.5 | na^i^ | 34 | -100.1 | 66.45 | -217.4 | 22.3 | | na^i^ |
| Turbidity | NTU^j^ | 83 | 154.8 | 86.08 | 9.8 | 430.0 | 18.1 | 34 | 178.8 | 98.79 | 23.8 | 356.0 | | 5.3 |
| Total suspended solids | mg/L | 83 | 267.8 | 110.05 | 26.0 | 458.0 | 15.5 | 34 | 342.9 | 125.33 | 48.0 | 581.0 | | -8.2 |
| Electronic conductivity | µS/cm | 83 | 1137.4 | 471.09 | 475.0 | 3010.0 | na^i^ | 34 | 1191.1 | 343.72 | 733.0 | 1871.0 | | na^i^ |
| Dissolved oxygen | mg/L | 83 | 8.8 | 0.89 | 6.5 | 11.0 | 16.2 | 34 | 8.3 | 1.03 | 6.4 | 10.7 | | 21.0 |
| Colour | Pa/Co | 83 | 1448.1 | 647.98 | 106.0 | 2941.0 | 8.8 | 34 | 1593.5 | 761.50 | 226.0 | 2924.0 | | -0.4 |
| Temperature | °C | 83 | 16.8 | 4.03 | 7.2 | 22.5 | na^i^ | 34 | 18.0 | 4.14 | 7.3 | 22.6 | | na^i^ |
| Biochemical oxygen demand | mg/L | 83 | 23.1 | 9.35 | 8.0 | 40.0 | 33.4 | 34 | 12.1 | 7.32 | 2.0 | 32.0 | | 65.1 |
| Chemical oxygen demand | mg/L | 83 | 94.0 | 31.13 | 16.2 | 161.5 | 27.2 | 34 | 90.7 | 29.89 | 47.6 | 177.0 | | 30.0 |
| Ammonia‒nitrogen | mg/L | 83 | 0.5 | 0.23 | 0.2 | 1.2 | -25.0 | 34 | 0.3 | 0.14 | 0.1 | 0.8 | | 25.0 |
| Nitrate‒nitrogen | mg/L | 83 | 10.7 | 7.92 | 0.9 | 37.9 | -20.2 | 34 | 16.3 | 4.89 | 6.2 | 34.5 | | -83.1 |
| Ortho‒phosphate‒phosphorus | mg/L | 83 | 48.0 | 13.76 | 23.1 | 76.3 | 18.9 | 34 | 16.3 | 3.00 | 3.0 | 38.1 | | 72.4 |
| Element | | | | | | | | | | | | | | |
| Aluminium | mg/L | 54 | 2.33 | 1.321 | 0.21 | 5.12 | -9.39 | 39 | 1.56 | 0.880 | 0.35 | 3.71 | 26.76 | |
| Boron | mg/L | 26 | 0.55 | 0.211 | 0.33 | 0.92 | 3.50 | 23 | 0.44 | 0.202 | 0.26 | 0.91 | 22.81 | |
| Calcium | mg/L | 52 | 42.49 | 4.386 | 31.32 | 48.65 | -17.77 | 37 | 77.22 | 42.765 | 39.17 | 150.14 | -114.02 | |
| Cadmium | mg/L | 36 | 5.82 | 2.238 | 2.28 | 9.84 | 20.92 | 30 | 4.61 | 2.126 | 1.73 | 8.90 | 37.36 | |
| Chromium | mg/L | 46 | 3.22 | 1.736 | 0.93 | 6.78 | -0.63 | 40 | 2.86 | 1.328 | 1.24 | 5.16 | 10.63 | |
| Copper | mg/L | 54 | 1.15 | 0.385 | 0.63 | 1.94 | 20.14 | 45 | 0.98 | 0.308 | 0.62 | 1.74 | 31.94 | |
| Iron | mg/L | 42 | 5.45 | 1.657 | 3.34 | 9.40 | 14.98 | 36 | 5.03 | 1.475 | 2.81 | 6.86 | 21.53 | |
| Potassium | mg/L | 8 | 44.90 | 2.827 | 41.21 | 48.07 | 25.37 | 8 | 56.58 | 19.919 | 36.93 | 77.55 | 5.95 | |
| Magnesium | mg/L | 54 | 17.77 | 3.477 | 12.22 | 22.55 | -3.55 | 42 | 12.84 | 6.124 | 5.36 | 22.80 | 25.17 | |
| Manganese | mg/L | 54 | 0.35 | 0.249 | 0.09 | 0.82 | 64.29 | 45 | 0.46 | 0.212 | 0.19 | 0.77 | 53.06 | |
| Sodium | mg/L | 8 | 55.09 | 11.391 | 42.72 | 66.71 | 12.11 | 8 | 55.85 | 12.850 | 42.23 | 74.21 | 10.90 | |
| Nickel | mg/L | 50 | 0.10 | 0.091 | 0.00 | 0.23 | -100.0 | 41 | 0.05 | 0.077 | 0.00 | 0.20 | 0.00 | |
| Zinc | mg/L | 32 | 3.12 | 0.872 | 1.76 | 4.60 | 26.59 | 29 | 2.78 | 0.859 | 1.62 | 4.27 | 34.59 | |
|  |  | 7‒day outflow HC T11^k^ | | | | | | 7‒day outflow HC T12^l^ | | | | | | |
| pH | ‒ | 83 | 7.7 | 1.21 | 5.9 | 9.9 | na^i^ | 34 | 9.8 | 1.54 | 7.1 | 12.3 | na^i^ | |
| Redox potential | mV | 83 | -4.4 | 59.67 | -108.3 | 78.1 | na^i^ | 34 | -95.5 | 88.21 | -216.7 | 157.8 | na^i^ | |
| Turbidity | NTU^j^ | 83 | 185.7 | 49.24 | 65.1 | 281.0 | 1.7 | 34 | 245.8 | 96.29 | 60.0 | 497.0 | -30.1 | |
| Total suspended solids | mg/L | 83 | 302.6 | 61.44 | 147.0 | 434.0 | 4.5 | 34 | 423.4 | 114.04 | 120.0 | 692.0 | -33.6 | |
| Electronic conductivity | µS/cm | 83 | 1003.0 | 306.88 | 492.0 | 2460.0 | na^i^ | 34 | 1107.1 | 299.47 | 734.0 | 1814.0 | na^i^ | |
| Dissolved oxygen | mg/L | 83 | 10.5 | 0.91 | 7.9 | 12.0 | 0.0 | 34 | 9.8 | 1.19 | 5.4 | 11.7 | 6.7 | |
| Colour | Pa/Co | 83 | 1644.8 | 489.96 | 718.0 | 2889.0 | -3.6 | 34 | 2040.5 | 757.57 | 688.0 | 3282.0 | -28.5 | |
| Temperature | °C | 83 | 16.6 | 3.87 | 7.6 | 22.1 | na^i^ | 34 | 17.7 | 4.20 | 7.4 | 22.3 | na^i^ | |
| Biochemical oxygen demand | mg/L | 83 | 16.6 | 7.07 | 4.0 | 38.0 | 52.2 | 34 | 8.3 | 4.23 | 0.0 | 18.0 | 76.1 | |
| Chemical oxygen demand | mg/L | 83 | 100.8 | 27.65 | 11.6 | 159.5 | 22.0 | 34 | 103.1 | 16.10 | 75.6 | 135.0 | 20.2 | |
| Ammonia‒nitrogen | mg/L | 83 | 0.3 | 0.13 | 0.0 | 0.8 | 25.0 | 34 | 0.3 | 0.11 | 0.1 | 0.5 | 25.0 | |
| Nitrate‒nitrogen | mg/L | 83 | 8.5 | 8.42 | 0.4 | 34.5 | 4.5 | 34 | 15.0 | 8.59 | 3.7 | 38.7 | -68.5 | |
| Ortho‒phosphate‒phosphorus | mg/L | 83 | 43.0 | 13.78 | 20.25 | 79.4 | 27.2 | 34 | 17.3 | 5.63 | 3.8 | 32.8 | 70.7 | |
| Element | | | | | | | | | | | | | | |
| Aluminium | mg/L | 54 | 2.98 | 1.218 | 1.61 | 6.14 | -39.91 | 24 | 3.61 | 2.306 | 0.87 | 6.67 | -69.48 | |
| Boron | mg/L | 26 | 0.54 | 0.160 | 0.34 | 0.77 | 5.26 | 20 | 0.39 | 0.078 | 0.30 | 0.51 | 31.58 | |
| Calcium | mg/L | 52 | 37.39 | 4.030 | 30.58 | 45.66 | -3.63 | 22 | 145.67 | 92.506 | 40.36 | 243.66 | -303.74 | |
| Cadmium | mg/L | 36 | 6.40 | 1.984 | 3.86 | 9.72 | 13.59 | 24 | 6.87 | 2.628 | 3.33 | 10.27 | 6.66 | |
| Chromium | mg/L | 46 | 4.76 | 1.215 | 2.83 | 6.68 | -48.75 | 34 | 4.75 | 2.021 | 2.57 | 6.89 | -48.44 | |
| Copper | mg/L | 54 | 1.30 | 0.301 | 0.80 | 1.76 | 9.72 | 36 | 1.47 | 0.247 | 1.14 | 1.80 | -2.08 | |
| Iron | mg/L | 42 | 7.02 | 1.801 | 3.58 | 9.36 | -9.52 | 30 | 8.69 | 2.012 | 6.48 | 10.99 | -35.57 | |
| Potassium | mg/L | 8 | 45.77 | 5.160 | 39.87 | 51.00 | 23.92 | 8 | 59.62 | 20.132 | 39.79 | 79.86 | 0.90 | |
| Magnesium | mg/L | 54 | 16.24 | 1.971 | 11.76 | 18.35 | 5.36 | 30 | 12.97 | 3.785 | 7.71 | 17.80 | 24.42 | |
| Manganese | mg/L | 54 | 1.01 | 0.223 | 0.75 | 1.38 | -3.06 | 36 | 0.86 | 0.457 | 0.33 | 1.42 | 12.24 | |
| Sodium | mg/L | 8 | 55.22 | 11.852 | 41.86 | 67.68 | 11.90 | 8 | 55.59 | 12.232 | 42.05 | 68.09 | 11.31 | |
| Nickel | mg/L | 50 | 0.09 | 0.081 | 0.00 | 0.20 | -80.00 | 32 | 0.04 | 0.033 | 0.00 | 0.10 | 20.00 | |
| Zinc | mg/L | 32 | 3.90 | 0.972 | 1.90 | 5.10 | 8.02 | 26 | 4.32 | 0.787 | 3.01 | 5.69 | -1.65 | |

^a^ HRT, hydraulic retention time

^b^ T9, treatment system with only *Phragmites australis*

^c^ T10, treatment system with *P. australis* and ochre pellets

^d^ n, number of tested samples

^e^ SD, standard deviation

^f^ Min, minimum

^g^ Max, maximum

^h^ Rem, removal

^i^ na, not applicable

^j^ NTU, nephelometric turbidity unit

^k^ T11, treatment system without *P. australis* or ochre pellets

^l^ T12, treatment system with ochre pellets only

**Online Resource 3** (Continued)

| d) LC‒SGW (7‒day HRT^a^) | | | | | | | | | | | | | | |
| --- | --- | --- | --- | --- | --- | --- | --- | --- | --- | --- | --- | --- | --- | --- |
|  |  | 7‒day outflow LC T13^b^ | | | | | | 7‒day outflow LC T14^c^ | | | | | | |
| Parameter | Unit | n^d^ | Mean | SD^e^ | Min^f^ | Max^g^ | Rem^h^ (%) | n^d^ | Mean | SD^e^ | Min^f^ | Max^g^ | | Rem^h^ (%) |
| pH | ‒ | 83 | 6.9 | 0.61 | 6.2 | 9.0 | na^i^ | 34 | 10.3 | 1.33 | 7.7 | 12.3 | | na^i^ |
| Redox potential | mV | 83 | 31.0 | 28.12 | -75.5 | 68.9 | na^i^ | 34 | -130.8 | 63.74 | -217.6 | -1.8 | | na^i^ |
| Turbidity | NTU^j^ | 83 | 18.9 | 11.05 | 2.8 | 52.1 | 17.5 | 34 | 25.1 | 16.21 | 2.9 | 77.8 | | -9.6 |
| Total suspended solids | mg/L | 83 | 27.7 | 16.48 | 2.8 | 72.0 | 30.6 | 34 | 37.5 | 15.62 | 4.0 | 74.0 | | 6.0 |
| Electronic conductivity | µS/cm | 83 | 161.4 | 42.91 | 103.7 | 324.0 | na^i^ | 34 | 306.8 | 118.32 | 166.2 | 774.0 | | na^i^ |
| Dissolved oxygen | mg/L | 83 | 9.3 | 1.24 | 4.9 | 12.0 | 10.6 | 34 | 8.7 | 0.94 | 7.0 | 10.7 | | 16.3 |
| Colour | Pa/Co | 83 | 159.1 | 56.83 | 48.0 | 391.0 | 25.8 | 34 | 250.6 | 120.15 | 47.0 | 647.0 | | -16.8 |
| Temperature | °C | 83 | 15.9 | 4.18 | 7.3 | 22.4 | na^i^ | 34 | 17.3 | 4.31 | 7.3 | 22.3 | | na^i^ |
| Biochemical oxygen demand | mg/L | 83 | 13.4 | 5.63 | 2.0 | 30.0 | 23.9 | 34 | 5.5 | 6.00 | 0.0 | 28.0 | | 68.8 |
| Chemical oxygen demand | mg/L | 83 | 31.3 | 11.95 | 7.9 | 77.5 | -8.3 | 34 | 29.2 | 10.71 | 20.3 | 63.4 | | -1.0 |
| Ammonia‒nitrogen | mg/L | 83 | 0.1 | 0.07 | 0.0 | 0.5 | 50.0 | 34 | 0.1 | 0.07 | 0.0 | 0.3 | | 50.0 |
| Nitrate‒nitrogen | mg/L | 83 | 1.3 | 0.77 | 0.0 | 4.8 | 0.0 | 34 | 0.7 | 0.77 | 0.0 | 3.7 | | 46.2 |
| Ortho‒phosphate‒phosphorus | mg/L | 83 | 11.9 | 6.36 | 3.3 | 32.0 | -41.7 | 34 | 3.0 | 1.77 | 1.5 | 8.5 | | 64.3 |
| Element | | | | | | | | | | | | | | |
| Aluminium | mg/L | 44 | 0.12 | 0.094 | 0.00 | 0.24 | 76.92 | 24 | 0.37 | 0.232 | 0.08 | 0.69 | 28.85 | |
| Boron | mg/L | 24 | 0.13 | 0.069 | 0.08 | 0.29 | 7.14 | 20 | 0.08 | 0.005 | 0.07 | 0.09 | 42.86 | |
| Calcium | mg/L | 52 | 11.44 | 0.944 | 10.01 | 13.12 | -8.54 | 22 | 60.11 | 13.881 | 38.50 | 75.27 | -470.30 | |
| Cadmium | mg/L | 32 | 0.08 | 0.097 | 0.00 | 0.27 | 11.11 | 24 | 0.02 | 0.021 | 0.00 | 0.06 | 77.78 | |
| Chromium | mg/L | 42 | 0.05 | 0.069 | 0.00 | 0.21 | -25.00 | 34 | 0.04 | 0.031 | 0.00 | 0.07 | 0.00 | |
| Copper | mg/L | 48 | 0.07 | 0.081 | 0.00 | 0.22 | 56.25 | 36 | 0.04 | 0.032 | 0.00 | 0.08 | 75.00 | |
| Iron | mg/L | 38 | 0.14 | 0.080 | 0.04 | 0.28 | 33.33 | 30 | 0.39 | 0.218 | 0.16 | 0.65 | -85.71 | |
| Potassium | mg/L | 8 | 2.99 | 0.216 | 2.72 | 3.29 | 25.99 | 8 | 17.59 | 16.141 | 2.35 | 33.47 | -335.40 | |
| Magnesium | mg/L | 46 | 1.55 | 0.195 | 1.18 | 1.91 | -6.90 | 30 | 0.84 | 0.224 | 0.53 | 1.08 | 42.07 | |
| Manganese | mg/L | 48 | 0.05 | 0.077 | 0.00 | 0.20 | 70.59 | 36 | 0.04 | 0.033 | 0.00 | 0.09 | 76.47 | |
| Sodium | mg/L | 8 | 13.91 | 1.648 | 12.10 | 15.47 | 2.86 | 8 | 15.42 | 3.280 | 12.09 | 18.59 | -7.68 | |
| Nickel | mg/L | 44 | 0.05 | 0.081 | 0.00 | 0.18 | -25.00 | 32 | 0.00 | 0.012 | 0.00 | 0.06 | 100.00 | |
| Zinc | mg/L | 28 | 0.11 | 0.094 | 0.00 | 0.32 | 47.62 | 24 | 0.06 | 0.050 | 0.00 | 0.14 | 71.43 | |
|  |  | 7‒day outflow LC T15^k^ | | | | | | 7‒day outflow LC T16^l^ | | | | | | |
| pH | ‒ | 83 | 7.5 | 0.72 | 6.4 | 9.3 | na^i^ | 34 | 10.5 | 1.05 | 8.0 | 12.2 | na^i^ | |
| Redox potential | mV | 83 | 1.8 | 33.00 | -87.9 | 53.2 | na^i^ | 34 | -131.3 | 72.36 | -217.6 | 156.0 | na^i^ | |
| Turbidity | NTU^j^ | 83 | 16.5 | 7.27 | 5.7 | 34.1 | 27.9 | 34 | 40.9 | 25.03 | 4.0 | 113.0 | -78.6 | |
| Total suspended solids | mg/L | 83 | 25.0 | 10.96 | 7.0 | 56.0 | 37.3 | 34 | 55.2 | 24.85 | 4.0 | 104.0 | -38.3 | |
| Electronic conductivity | µS/cm | 83 | 144.0 | 32.28 | 97.7 | 263.0 | na^i^ | 34 | 290.2 | 135.74 | 148.0 | 768.0 | na^i^ | |
| Dissolved oxygen | mg/L | 83 | 11.0 | 1.11 | 8.1 | 14.3 | -5.8 | 34 | 10.1 | 0.84 | 8.3 | 11.2 | 2.9 | |
| Colour | Pa/Co | 83 | 152.6 | 41.05 | 51.0 | 258.0 | 28.9 | 34 | 283.8 | 115.21 | 48.0 | 544.0 | -32.3 | |
| Temperature | °C | 83 | 15.3 | 4.23 | 6.7 | 22.2 | na^i^ | 34 | 17.0 | 4.15 | 7.9 | 22.1 | na^i^ | |
| Biochemical oxygen demand | mg/L | 83 | 6.7 | 4.85 | 0.0 | 22.0 | 61.9 | 34 | 5.4 | 3.95 | 0.0 | 20.0 | 69.3 | |
| Chemical oxygen demand | mg/L | 83 | 17.2 | 6.95 | 6.0 | 36.7 | 40.5 | 34 | 19.9 | 7.28 | 3.9 | 32.2 | 31.1 | |
| Ammonia‒nitrogen | mg/L | 83 | 0.1 | 0.04 | 0.0 | 0.3 | 50.0 | 34 | 0.1 | 0.15 | 0.0 | 0.8 | 50.0 | |
| Nitrate‒nitrogen | mg/L | 83 | 1.0 | 0.64 | 0.0 | 4.0 | 23.1 | 34 | 0.3 | 0.28 | 0.0 | 1.1 | 76.9 | |
| Ortho‒phosphate‒phosphorus | mg/L | 83 | 8.5 | 4.03 | 2.6 | 19.6 | -10.0 | 34 | 3.7 | 1.29 | 1.2 | 6.6 | 56.0 | |
| Element | | | | | | | | | | | | | | |
| Aluminium | mg/L | 44 | 0.36 | 0.189 | 0.09 | 0.75 | 30.77 | 24 | 0.73 | 0.420 | 0.20 | 1.40 | -40.38 | |
| Boron | mg/L | 24 | 0.12 | 0.064 | 0.08 | 0.26 | 14.29 | 20 | 0.08 | 0.006 | 0.07 | 0.09 | 42.86 | |
| Calcium | mg/L | 52 | 10.74 | 0.739 | 9.44 | 12.12 | -1.90 | 22 | 65.46 | 37.361 | 23.48 | 104.98 | -521.06 | |
| Cadmium | mg/L | 32 | 0.09 | 0.083 | 0.00 | 0.21 | 0.00 | 24 | 0.05 | 0.046 | 0.00 | 0.11 | 44.44 | |
| Chromium | mg/L | 42 | 0.07 | 0.074 | 0.00 | 0.21 | -75.00 | 34 | 0.06 | 0.054 | 0.00 | 0.12 | -50.00 | |
| Copper | mg/L | 48 | 0.10 | 0.091 | 0.00 | 0.26 | 37.50 | 36 | 0.06 | 0.057 | 0.00 | 0.13 | 62.50 | |
| Iron | mg/L | 38 | 0.20 | 0.100 | 0.07 | 0.30 | 4.76 | 30 | 0.93 | 0.759 | 0.15 | 1.91 | -342.86 | |
| Potassium | mg/L | 8 | 3.62 | 0.438 | 3.07 | 4.22 | 10.40 | 8 | 20.16 | 19.003 | 2.26 | 38.75 | -399.01 | |
| Magnesium | mg/L | 46 | 1.38 | 0.161 | 1.03 | 1.64 | 4.83 | 30 | 0.78 | 0.330 | 0.36 | 1.16 | 46.21 | |
| Manganese | mg/L | 48 | 0.06 | 0.074 | 0.00 | 0.21 | 64.71 | 36 | 0.10 | 0.094 | 0.00 | 0.21 | 41.18 | |
| Sodium | mg/L | 8 | 13.15 | 1.199 | 11.83 | 14.36 | 8.17 | 8 | 15.69 | 5.272 | 10.55 | 21.03 | -9.57 | |
| Nickel | mg/L | 44 | 0.05 | 0.080 | 0.00 | 0.18 | -25.00 | 32 | 0.01 | 0.010 | 0.00 | 0.05 | 75.00 | |
| Zinc | mg/L | 28 | 0.13 | 0.068 | 0.01 | 0.25 | 38.10 | 24 | 0.11 | 0.089 | 0.00 | 0.26 | 47.62 | |

^a^ HRT, hydraulic retention time

^b^ T13, treatment system with only *Phragmites australis*

^c^ T14, treatment system with *P. australis* and ochre pellets

^f^ Min, minimum

^g^ Max, maximum

^h^ Rem, removal

^i^ na, not applicable

^j^ NTU, nephelometric turbidity unit

^k^ T15, treatment system without *P. australis* or ochre pellets

^l^ T16, treatment system with only ochre pellets

**Online Resource 3** (Continued)

| e) Outflow of control wetlands | | | | | | | | | | | |
| --- | --- | --- | --- | --- | --- | --- | --- | --- | --- | --- | --- |
|  |  | 2‒day outflow TW^a^ C1^b^ | | | | | 2‒day outflow TW^a^ C2^c^ | | | | |
| Parameter | Unit | n^d^ | Mean | SD^e^ | Min^f^ | Max^g^ | n^d^ | Mean | SD^e^ | Min^f^ | Max^g^ |
| pH | ‒ | 85 | 6.7 | 0.39 | 6.0 | 7.7 | 85 | 7.4 | 0.60 | 6.2 | 9.3 |
| Redox potential | mV | 85 | 42.2 | 16.50 | -7.7 | 70.8 | 85 | 9.6 | 28.10 | -72.2 | 78.4 |
| Turbidity | NTU^h^ | 85 | 9.3 | 6.61 | 2.0 | 39.3 | 85 | 4.2 | 4.37 | 1.2 | 32.5 |
| Total suspended solids | mg/L | 85 | 14.3 | 8.16 | 0.0 | 35.0 | 85 | 3.9 | 2.93 | 0.0 | 15.0 |
| Electronic conductivity | µS/cm | 85 | 84.4 | 12.15 | 57.2 | 117.7 | 85 | 81.5 | 9.94 | 57.2 | 116.2 |
| Dissolved oxygen | mg/L | 85 | 9.0 | 0.87 | 7.2 | 11.8 | 85 | 10.4 | 0.70 | 8.9 | 12.0 |
| Colour | Pa/Co | 85 | 44.3 | 30.56 | 6.0 | 168.0 | 85 | 8.6 | 7.66 | 0.0 | 34.0 |
| Temperature | °C | 85 | 16.5 | 3.76 | 5.9 | 22.4 | 85 | 16.8 | 4.04 | 6.0 | 22.8 |
| Biochemical oxygen demand | mg/L | 85 | 7.3 | 3.45 | 0.0 | 18.0 | 85 | 5.4 | 4.03 | 0.0 | 18.0 |
| Chemical oxygen demand | mg/L | 85 | 15.9 | 7.74 | 4.9 | 42.8 | 85 | 6.3 | 2.84 | 1.0 | 14.3 |
| Ammonia‒nitrogen | mg/L | 85 | 0.1 | 0.12 | 0.0 | 0.6 | 85 | 0.1 | 0.14 | 0.0 | 0.6 |
| Nitrate‒nitrogen | mg/L | 85 | 1.1 | 0.75 | 0.1 | 3.8 | 85 | 0.8 | 0.53 | 0.0 | 3.6 |
| Ortho‒phosphate‒phosphorus | mg/L | 85 | 2.8 | 1.82 | 0.9 | 10.6 | 85 | 2.4 | 0.63 | 0.9 | 4.3 |
| Element | | | | | | | | | | | |
| Aluminium | mg/L | 24 | 0.01 | 0.006 | 0.00 | 0.02 | 24 | 0.01 | 0.007 | 0.00 | 0.02 |
| Boron | mg/L | 20 | 0.02 | 0.018 | 0.00 | 0.05 | 20 | 0.03 | 0.009 | 0.01 | 0.05 |
| Calcium | mg/L | 28 | 9.96 | 0.549 | 9.06 | 10.90 | 28 | 9.78 | 0.552 | 8.90 | 10.67 |
| Cadmium | mg/L | 24 | 0.01 | 0.006 | 0.00 | 0.02 | 24 | 0.00 | 0.006 | 0.00 | 0.02 |
| Chromium | mg/L | 34 | 0.00 | 0.005 | 0.00 | 0.01 | 34 | 0.00 | 0.005 | 0.00 | 0.01 |
| Copper | mg/L | 36 | 0.01 | 0.006 | 0.00 | 0.02 | 36 | 0.01 | 0.008 | 0.00 | 0.03 |
| Iron | mg/L | 30 | 0.02 | 0.007 | 0.00 | 0.04 | 30 | 0.02 | 0.009 | 0.00 | 0.04 |
| Potassium | mg/L | 8 | 0.35 | 0.049 | 0.27 | 0.42 | 8 | 0.69 | 0.261 | 0.40 | 0.98 |
| Magnesium | mg/L | 30 | 1.10 | 0.123 | 0.77 | 1.27 | 30 | 1.10 | 0.138 | 0.78 | 1.30 |
| Manganese | mg/L | 36 | 0.01 | 0.010 | 0.00 | 0.04 | 36 | 0.00 | 0.009 | 0.00 | 0.03 |
| Sodium | mg/L | 8 | 6.62 | 0.721 | 5.82 | 7.31 | 8 | 6.69 | 0.869 | 5.78 | 7.58 |
| Nickel | mg/L | 32 | 0.01 | 0.023 | 0.00 | 0.09 | 32 | 0.01 | 0.023 | 0.00 | 0.10 |
| Zinc | mg/L | 26 | 0.03 | 0.009 | 0.00 | 0.03 | 26 | 0.02 | 0.010 | 0.00 | 0.04 |
|  |  | 7‒day outflow TW^a^ C3^i^ | | | | | 7‒day outflow TW^a^ C4^j^ | | | | |
| pH | ‒ | 83 | 6.6 | 0.39 | 6.0 | 7.3 | 83 | 7.1 | 0.52 | 5.9 | 8.9 |
| Redox potential | mV | 83 | 44.1 | 17.06 | 6.1 | 76.4 | 83 | 25.1 | 24.68 | -60.9 | 89.8 |
| Turbidity | NTU^h^ | 83 | 12.7 | 12.56 | 1.5 | 69.9 | 83 | 3.7 | 3.47 | 1.3 | 30.1 |
| Total suspended solids | mg/L | 83 | 17.8 | 13.69 | 0.0 | 67.0 | 83 | 4.3 | 5.79 | 0.0 | 38.0 |
| Electronic conductivity | µS/cm | 83 | 92.9 | 27.28 | 60.7 | 167.2 | 83 | 87.1 | 20.83 | 58.4 | 163.2 |
| Dissolved oxygen | mg/L | 83 | 8.9 | 1.09 | 5.7 | 11.2 | 83 | 10.8 | 1.07 | 8.4 | 13.2 |
| Colour | Pa/Co | 83 | 56.1 | 31.45 | 11.0 | 143.0 | 83 | 12.7 | 9.73 | 0.0 | 42.0 |
| Temperature | °C | 83 | 15.1 | 4.20 | 6.2 | 22.2 | 83 | 15.5 | 4.17 | 7.2 | 22.0 |
| Biochemical oxygen demand | mg/L | 83 | 9.1 | 5.05 | 0.0 | 22.0 | 83 | 6.7 | 4.65 | 0.0 | 24.0 |
| Chemical oxygen demand | mg/L | 83 | 17.6 | 6.74 | 6.3 | 35.2 | 83 | 7.0 | 2.48 | 1.6 | 17.3 |
| Ammonia‒nitrogen | mg/L | 83 | 0.1 | 0.04 | 0.0 | 0.2 | 83 | 0.1 | 0.05 | 0.0 | 0.2 |
| Nitrate‒nitrogen | mg/L | 83 | 0.9 | 0.42 | 0.0 | 2.8 | 83 | 0.8 | 0.54 | 0.0 | 3.0 |
| Ortho‒phosphate‒phosphorus | mg/L | 83 | 3.4 | 1.47 | 0.4 | 7.4 | 83 | 2.4 | 0.86 | 0.5 | 4.6 |
| Element | | | | | | | | | | | |
| Aluminium | mg/L | 39 | 0.08 | 0.092 | 0.00 | 0.20 | 39 | 0.09 | 0.101 | 0.00 | 0.22 |
| Boron | mg/L | 23 | 0.05 | 0.061 | 0.00 | 0.19 | 23 | 0.05 | 0.059 | 0.00 | 0.19 |
| Calcium | mg/L | 49 | 9.67 | 0.591 | 8.41 | 10.70 | 49 | 9.51 | 0.476 | 8.40 | 10.40 |
| Cadmium | mg/L | 30 | 0.04 | 0.071 | 0.00 | 0.19 | 30 | 0.05 | 0.071 | 0.00 | 0.20 |
| Chromium | mg/L | 40 | 0.03 | 0.063 | 0.00 | 0.19 | 40 | 0.03 | 0.063 | 0.00 | 0.19 |
| Copper | mg/L | 45 | 0.04 | 0.073 | 0.00 | 0.19 | 45 | 0.05 | 0.078 | 0.00 | 0.20 |
| Iron | mg/L | 36 | 0.05 | 0.069 | 0.00 | 0.22 | 36 | 0.05 | 0.066 | 0.00 | 0.20 |
| Potassium | mg/L | 8 | 0.50 | 0.492 | 0.03 | 1.01 | 8 | 0.52 | 0.127 | 0.39 | 0.70 |
| Magnesium | mg/L | 42 | 1.20 | 0.119 | 0.88 | 1.40 | 42 | 1.16 | 0.120 | 0.83 | 1.37 |
| Manganese | mg/L | 45 | 0.04 | 0.070 | 0.00 | 0.19 | 45 | 0.04 | 0.069 | 0.00 | 0.18 |
| Sodium | mg/L | 8 | 6.80 | 0.085 | 6.69 | 6.90 | 8 | 6.35 | 0.105 | 6.24 | 6.56 |
| Nickel | mg/L | 41 | 0.04 | 0.075 | 0.00 | 0.18 | 41 | 0.04 | 0.075 | 0.00 | 0.18 |
| Zinc | mg/L | 29 | 0.04 | 0.070 | 0.00 | 0.24 | 29 | 0.04 | 0.061 | 0.00 | 0.22 |

^a^ TW, tap water

^b^ C1, treatment system 2–day with only *Phragmites australis*

^c^ C3, treatment system 7-day with only *Phragmites australis*

^d^ n, number of tested samples

^e^ SD, standard deviation

^f^ Min, minimum

^g^ Max, maximum

^h^ NTU, nephelometric turbidity unit

^i^ C2 treatment system 2–day with only TW

^j^ C4, treatment system 7–day with only TW
