# Supplementary material for: Phytoremediation performance of floating treatment wetlands with pelletized mine water sludge for synthetic greywater treatment
Source: J Environ Health Sci Eng. 2019 Apr 18;17(2):581–608. doi: 10.1007/s40201-019-00372-z (PMC6985343; doi:10.1007/s40201-019-00372-z)
Supplement: Supplementary file 4 — (DOCX 34 kb) [file 40201_2019_372_MOESM4_ESM.docx]

**Phytoremediation performance of floating treatment wetlands with pelletized mine water sludge for synthetic greywater treatment**

*Journal of Environmental Health Science and Engineering*

**Suhail N. Abed, Suhad A. Almuktar, Miklas Scholz**

Corresponding author: Miklas Scholz

Civil Engineering Research Group, School of Computing, Science and Engineering, The University of Salford, Newton Building, Salford M5 4WT, England, United Kingdom.

Division of Water Resources Engineering, Department of Building and Environmental Technology, Faculty of Engineering, Lund University, P.O. Box 118, 221 00 Lund, Sweden.

Department of Civil Engineering Science, School of Civil Engineering and the Built Environment, University of Johannesburg, Kingsway Campus, PO Box 524, Aukland Park 2006, Johannesburg, South Africa

E‒mail address: miklas.scholz@tvrl.lth.se

**Online Resource 4** Significance values of statistical analysis concerning the comparison between treated and untreated greywater samples of *(a)* high concentration greywater (HC‒SGW) at 2‒day HRT, *(b)* low concentration greywater (LC‒SGW) at 2‒day HRT, *(c)* HC‒SGW at 7‒day HRT, and *(d)* LC‒SGW at 7‒day HRT

| a) HC‒SGW: Comparisons at 2‒day HRT^a^ (inflow & outflow) | | | | | | | | | | | | | |
| --- | --- | --- | --- | --- | --- | --- | --- | --- | --- | --- | --- | --- | --- |
|  | | Inflow & T1^b^ | | | Inflow & T2^c^ | | | Inflow & T3^d^ | | | Inflow & T4^e^ | | |
| Parameter | Unit | Shapiro‒Wilk (p value) | Statistical test^f^ | Significance (p value) | Shapiro‒Wilk (p value) | Statistical test^f^ | Significance (p value) | Shapiro‒Wilk (p value) | Statistical test^f^ | Significance (p value) | Shapiro‒Wilk (p value) | Statistical test^f^ | Significance (p value) |
| pH | ‒ | <0.001 | M‒W | <0.001 | 0.147 | T‒test | 0.183 | <0.001 | M‒W | 0.027 | 0.149 | T‒test | 0.313 |
| Redox potential | mV | <0.001 | M‒W | <0.001 | 0.086 | T‒test | 0.239 | <0.001 | M‒W | 0.004 | 0.081 | T‒test | 0.390 |
| Turbidity | NTU^g^ | 0.273 | T‒test | 0.121 | 0.001 | M‒W | 0.317 | 0.118 | T‒test | 0.676 | <0.001 | M‒W | 0.071 |
| Total suspended solids | mg/L | 0.113 | T‒test | 0.179 | <0.001 | M‒W | 0.001 | 0.217 | T‒test | 0.593 | <0.001 | M‒W | 0.603 |
| Electronic conductivity | µS/cm | 0.018 | M‒W | 0.904 | <0.001 | M‒W | 0.001 | 0.012 | M‒W | 0.376 | <0.001 | M‒W | <0.001 |
| Dissolved oxygen | mg/L | 0.010 | M‒W | <0.001 | 0.003 | M‒W | <0.001 | 0.005 | M‒W | 0.002 | 0.007 | M‒W | 0.001 |
| Colour | Pa/Co | 0.734 | T‒test | 0.313 | <0.001 | M‒W | 0.001 | 0.218 | T‒test | 0.274 | <0.001 | M‒W | 0.032 |
| Temperature | °C | <0.001 | M‒W | 0.678 | 0.003 | M‒W | 0.770 | 0.005 | M‒W | 0.923 | 0.005 | M‒W | 0.939 |
| Biochemical oxygen demand | mg/L | <0.001 | M‒W | <0.001 | 0.001 | M‒W | <0.001 | 0.002 | M‒W | <0.001 | 0.002 | M‒W | <0.001 |
| Chemical oxygen demand | mg/L | 0.008 | M‒W | <0.001 | 0.060 | T‒test | 0.001 | 0.005 | M‒W | <0.001 | 0.014 | M‒W | <0.001 |
| Ammonia‒nitrogen | mg/L | <0.001 | M‒W | 0.269 | <0.001 | M‒W | 0.050 | <0.001 | M‒W | 0.860 | <0.001 | M‒W | 0.545 |
| Nitrate‒nitrogen | mg/L | 0.006 | M‒W | <0.001 | 0.016 | M‒W | <0.001 | 0.005 | M‒W | 0.325 | <0.001 | M‒W | 0.006 |
| Ortho‒phosphate‒phosphorus | mg/L | 0.330 | T‒test | 0.002 | 0.002 | M‒W | <0.001 | 0.107 | T‒test | <0.001 | <0.001 | M‒W | <0.001 |
| Aluminium (Al) | mg/L | 0.001 | M‒W | 0.002 | <0.001 | M‒W | 0.083 | 0.009 | M‒W | 0.171 | <0.001 | M‒W | 0.696 |
| Boron (B) | mg/L | <0.001 | M‒W | 0.006 | <0.001 | M‒W | <0.001 | 0.002 | M‒W | 0.171 | 0.004 | M‒W | <0.001 |
| Calcium (Ca) | mg/L | 0.048 | M‒W | 0.001 | <0.001 | M‒W | <0.001 | 0.001 | M‒W | 0.001 | <0.001 | M‒W | <0.001 |
| Cadmium (Cd) | mg/L | <0.001 | M‒W | 0.007 | <0.001 | M‒W | <0.001 | <0.001 | M‒W | 0.159 | <0.001 | M‒W | 0.128 |
| Chromium (Cr) | mg/L | <0.001 | M‒W | <0.001 | <0.001 | M‒W | 0.005 | 0.004 | M‒W | 0.003 | <0.001 | M‒W | 0.104 |
| Copper (Cu) | mg/L | <0.001 | M‒W | <0.001 | <0.001 | M‒W | <0.001 | <0.001 | M‒W | 0.378 | <0.001 | M‒W | 0.005 |
| Iron (Fe) | mg/L | <0.001 | M‒W | 0.001 | <0.001 | M‒W | 0.019 | 0.002 | M‒W | 0.867 | <0.001 | M‒W | 0.140 |
| Potassium (K) | mg/L | 0.013 | M‒W | <0.001 | 0.002 | M‒W | 0.064 | 0.004 | M‒W | 0.014 | 0.003 | M‒W | 0.064 |
| Magnesium (Mg) | mg/L | <0.001 | M‒W | 0.348 | <0.001 | M‒W | <0.001 | 0.007 | M‒W | 0.120 | <0.001 | M‒W | <0.001 |
| Manganese (Mn) | mg/L | <0.001 | M‒W | <0.001 | <0.001 | M‒W | <0.001 | <0.001 | M‒W | 0.006 | <0.001 | M‒W | <0.001 |
| Sodium (Na) | mg/L | 0.005 | M‒W | 0.328 | 0.009 | M‒W | 0.355 | 0.007 | M‒W | 0.304 | 0.009 | M‒W | 0.411 |
| Nickel (Ni) | mg/L | <0.001 | M‒W | 0.005 | <0.001 | M‒W | <0.001 | <0.001 | M‒W | 0.169 | <0.001 | M‒W | 0.007 |
| Zinc (Zn) | mg/L | <0.001 | M‒W | 0.004 | <0.001 | M‒W | <0.001 | 0.004 | M‒W | 0.500 | <0.001 | M‒W | 0.208 |

^a^ HRT, hydraulic retention time

^b^ T1, treatment system with *P. australis* only

^c^ T2, treatment system with *P. australis* and ochre pellets

^d^ T3, treatment system without *P. australis* or ochre pellets

^e^ T4, treatment system with ochre pellets only

^f^ Shapiro‒Wilk (check for normality), normally distributed data, if p>0.05 using T‒test, and non‒normally distributed data, if p<0.05 using Mann‒Whitney U‒test; p value (M‒W), significant difference, if p<0.05, and not significant, if p>0.05

^g^ NTU, nephelometric turbidity unit

**Online Resource 4** (Continued)

| b) LC‒SGW: Comparisons at 2‒day HRT^a^ (inflow & outflow) | | | | | | | | | | | | | |
| --- | --- | --- | --- | --- | --- | --- | --- | --- | --- | --- | --- | --- | --- |
|  | | Inflow & T5^b^ | | | Inflow & T6^c^ | | | Inflow & T7^d^ | | | Inflow & T8^e^ | | |
| Parameter | Unit | Shapiro‒Wilk (p value) | Statistical test^f^ | Significance (p value) | Shapiro‒Wilk (p value) | Statistical test^f^ | Significance (p value) | Shapiro‒Wilk (p value) | Statistical test^f^ | Significance (p value) | Shapiro‒Wilk (p value) | Statistical test^f^ | Significance (p value) |
| pH | ‒ | 0.009 | M‒W | 0.821 | <0.001 | M‒W | <0.001 | 0.132 | T‒test | <0.001 | <0.001 | M‒W | <0.001 |
| Redox potential | mV | <0.001 | M‒W | 0.494 | <0.001 | M‒W | <0.001 | 0.013 | M‒W | <0.001 | <0.001 | M‒W | <0.001 |
| Turbidity | NTU^g^ | <0.001 | M‒W | 0.258 | <0.001 | M‒W | 0.016 | <0.001 | M‒W | 0.004 | <0.001 | M‒W | <0.001 |
| Total suspended solids | mg/L | <0.001 | M‒W | 0.050 | <0.001 | M‒W | 0.009 | <0.001 | M‒W | <0.001 | <0.001 | M‒W | <0.001 |
| Electronic conductivity | µS/cm | <0.001 | M‒W | 0.061 | <0.001 | M‒W | <0.001 | <0.001 | M‒W | 0.001 | <0.001 | M‒W | <0.001 |
| Dissolved oxygen | mg/L | 0.001 | M‒W | <0.001 | 0.012 | M‒W | <0.001 | <0.001 | M‒W | 0.846 | <0.001 | M‒W | 0.009 |
| Colour | Pa/Co | 0.963 | T‒test | 0.005 | <0.001 | M‒W | 0.001 | 0.483 | T‒test | <0.001 | 0.003 | M‒W | 0.032 |
| Temperature | °C | 0.002 | M‒W | 0.432 | 0.003 | M‒W | 0.215 | 0.015 | M‒W | 0.053 | 0.009 | M‒W | 0.096 |
| Biochemical oxygen demand | mg/L | <0.001 | M‒W | <0.001 | <0.001 | M‒W | <0.001 | <0.001 | M‒W | <0.001 | <0.001 | M‒W | <0.001 |
| Chemical oxygen demand | mg/L | <0.001 | M‒W | 0.060 | <0.001 | M‒W | 0.725 | <0.001 | M‒W | 0.811 | <0.001 | M‒W | 0.199 |
| Ammonia‒nitrogen | mg/L | <0.001 | M‒W | 0.003 | <0.001 | M‒W | 0.166 | <0.001 | M‒W | <0.001 | <0.001 | M‒W | 0.037 |
| Nitrate‒nitrogen | mg/L | <0.001 | M‒W | 0.003 | <0.001 | M‒W | <0.001 | <0.001 | M‒W | 0.687 | <0.001 | M‒W | <0.001 |
| Ortho‒phosphate‒phosphorus | mg/L | <0.001 | M‒W | 0.319 | <0.001 | M‒W | <0.001 | <0.001 | M‒W | 0.003 | <0.001 | M‒W | <0.001 |
| Aluminium (Al) | mg/L | <0.001 | M‒W | <0.001 | <0.001 | M‒W | 0.027 | <0.001 | M‒W | 0.192 | <0.001 | M‒W | 0.080 |
| Boron (B) | mg/L | <0.001 | M‒W | 0.009 | <0.001 | M‒W | <0.001 | <0.001 | M‒W | 0.001 | <0.001 | M‒W | 0.001 |
| Calcium (Ca) | mg/L | 0.059 | T‒test | <0.001 | <0.001 | M‒W | <0.001 | 0.387 | T‒test | <0.001 | <0.001 | M‒W | <0.001 |
| Cadmium (Cd) | mg/L | <0.001 | M‒W | <0.001 | <0.001 | M‒W | <0.001 | <0.001 | M‒W | <0.001 | <0.001 | M‒W | <0.001 |
| Chromium (Cr) | mg/L | <0.001 | M‒W | 0.071 | <0.001 | M‒W | 0.470 | <0.001 | M‒W | 0.014 | <0.001 | M‒W | 0.005 |
| Copper (Cu) | mg/L | <0.001 | M‒W | <0.001 | <0.001 | M‒W | <0.001 | <0.001 | M‒W | <0.001 | <0.001 | M‒W | <0.001 |
| Iron (Fe) | mg/L | <0.001 | M‒W | <0.001 | <0.001 | M‒W | 0.024 | <0.001 | M‒W | 0.001 | <0.001 | M‒W | 0.051 |
| Potassium (K) | mg/L | 0.122 | T‒test | 0.008 | 0.167 | T‒test | 0.028 | 0.125 | T‒test | 0.309 | 0.536 | T‒test | 0.050 |
| Magnesium (Mg) | mg/L | <0.001 | M‒W | 0.021 | <0.001 | M‒W | <0.001 | <0.001 | M‒W | 0.065 | <0.001 | M‒W | <0.001 |
| Manganese (Mn) | mg/L | <0.001 | M‒W | <0.001 | <0.001 | M‒W | <0.001 | <0.001 | M‒W | <0.001 | <0.001 | M‒W | <0.001 |
| Sodium (Na) | mg/L | 0.186 | T‒test | 0.476 | 0.007 | M‒W | 0.090 | 0.010 | M‒W | 0.382 | 0.043 | M‒W | 0.681 |
| Nickel (Ni) | mg/L | <0.001 | M‒W | <0.001 | <0.001 | M‒W | <0.001 | <0.001 | M‒W | <0.001 | <0.001 | M‒W | 0.410 |
| Zinc (Zn) | mg/L | <0.001 | M‒W | <0.001 | <0.001 | M‒W | <0.001 | <0.001 | M‒W | <0.001 | <0.001 | M‒W | <0.001 |

^a^ HRT, hydraulic retention time

^b^ T5, treatment system with *Phragmites australis* only

^c^ T6, treatment system with *P. australis* and ochre pellets

^d^ T7, treatment system without *P. australis* or ochre pellets

^e^ T8, treatment system with ochre pellets only

^f^ Shapiro‒Wilk (check for normality), normally distributed data, if p>0.05 using T‒test, and non‒normally distributed data, if p<0.05 using Mann‒Whitney U‒test; p value (M‒W), significant difference, if p<0.05, and not significant, if p>0.05

^g^ NTU, nephelometric turbidity unit

**Online Resource 4** (Continued)

| c) HC‒SGW: Comparisons at 7‒day HRT^a^ (inflow & outflow) | | | | | | | | | | | | | |
| --- | --- | --- | --- | --- | --- | --- | --- | --- | --- | --- | --- | --- | --- |
|  | | Inflow & T9^b^ | | | Inflow & T10^c^ | | | Inflow & T11^d^ | | | Inflow & T12^e^ | | |
| Parameter | Unit | Shapiro‒Wilk (p value) | Statistical test^f^ | Significance (p value) | Shapiro‒Wilk (p value) | Statistical test^f^ | Significance (p value) | Shapiro‒Wilk (p value) | Statistical test^f^ | Significance (p value) | Shapiro‒Wilk (p value) | Statistical test^f^ | Significance (p value) |
| pH | ‒ | <0.001 | M‒W | <0.001 | 0.002 | M‒W | <0.001 | <0.001 | M‒W | 0.004 | 0.021 | M‒W | <0.001 |
| Redox potential | mV | <0.001 | M‒W | <0.001 | 0.006 | M‒W | <0.001 | <0.001 | M‒W | 0.004 | 0.099 | T‒test | <0.001 |
| Turbidity | NTU^g^ | 0.003 | M‒W | 0.008 | 0.086 | T‒test | 0.573 | 0.471 | T‒test | 0.667 | <0.001 | M‒W | <0.001 |
| Total suspended solids | mg/L | 0.018 | M‒W | 0.014 | 0.009 | M‒W | 0.006 | 0.318 | T‒test | 0.127 | <0.001 | M‒W | <0.001 |
| Electronic conductivity | µS/cm | 0.216 | T‒test | 0.009 | <0.001 | M‒W | 0.002 | 0.042 | M‒W | 0.320 | <0.001 | M‒W | 0.149 |
| Dissolved oxygen | mg/L | 0.001 | M‒W | <0.001 | 0.001 | M‒W | <0.001 | 0.004 | M‒W | 0.301 | 0.001 | M‒W | 0.003 |
| Colour | Pa/Co | 0.313 | T‒test | 0.094 | 0.615 | T‒test | 0.967 | 0.285 | T‒test | 0.405 | 0.001 | M‒W | 0.001 |
| Temperature | °C | 0.002 | M‒W | 0.730 | 0.002 | M‒W | 0.371 | 0.004 | M‒W | 0.585 | 0.003 | M‒W | 0.502 |
| Biochemical oxygen demand | mg/L | <0.001 | M‒W | <0.001 | 0.001 | M‒W | <0.001 | <0.001 | M‒W | <0.001 | <0.001 | M‒W | <0.001 |
| Chemical oxygen demand | mg/L | 0.040 | M‒W | <0.001 | 0.022 | M‒W | <0.001 | 0.002 | M‒W | <0.001 | 0.001 | M‒W | <0.001 |
| Ammonia‒nitrogen | mg/L | <0.001 | M‒W | <0.001 | <0.001 | M‒W | 0.001 | <0.001 | M‒W | 0.091 | <0.001 | M‒W | 0.009 |
| Nitrate‒nitrogen | mg/L | 0.002 | M‒W | 0.266 | 0.006 | M‒W | <0.001 | <0.001 | M‒W | 0.204 | <0.001 | M‒W | <0.001 |
| Ortho‒phosphate‒phosphorus | mg/L | 0.146 | T‒test | <0.001 | <0.001 | M‒W | <0.001 | 0.195 | T‒test | <0.001 | <0.001 | M‒W | <0.001 |
| Aluminium (Al) | mg/L | 0.002 | M‒W | 0.302 | 0.045 | M‒W | 0.007 | 0.156 | T‒test | <0.001 | <0.001 | M‒W | 0.104 |
| Boron (B) | mg/L | <0.001 | M‒W | 0.099 | 0.002 | M‒W | <0.001 | 0.016 | M‒W | 0.593 | 0.018 | M‒W | <0.001 |
| Calcium (Ca) | mg/L | <0.001 | M‒W | 0.001 | 0.032 | M‒W | <0.001 | 0.003 | M‒W | 0.068 | <0.001 | M‒W | <0.001 |
| Cadmium (Cd) | mg/L | <0.001 | M‒W | 0.075 | <0.001 | M‒W | 0.001 | <0.001 | M‒W | 0.340 | <0.001 | M‒W | 0.119 |
| Chromium (Cr) | mg/L | 0.001 | M‒W | 0.688 | 0.001 | M‒W | 0.230 | <0.001 | M‒W | <0.001 | <0.001 | M‒W | 0.119 |
| Copper (Cu) | mg/L | 0.001 | M‒W | 0.011 | 0.001 | M‒W | <0.001 | 0.001 | M‒W | 0.177 | 0.001 | M‒W | 0.436 |
| Iron (Fe) | mg/L | 0.002 | M‒W | 0.064 | 0.001 | M‒W | 0.009 | 0.003 | M‒W | 0.193 | <0.001 | M‒W | <0.001 |
| Potassium (K) | mg/L | 0.002 | M‒W | <0.001 | 0.006 | M‒W | 1.000 | 0.002 | M‒W | <0.001 | 0.011 | M‒W | 1.000 |
| Magnesium (Mg) | mg/L | 0.001 | M‒W | 0.169 | <0.001 | M‒W | 0.001 | 0.024 | M‒W | 0.277 | <0.001 | M‒W | <0.001 |
| Manganese (Mn) | mg/L | <0.001 | M‒W | <0.001 | <0.001 | M‒W | <0.001 | <0.001 | M‒W | 0.023 | <0.001 | M‒W | 0.595 |
| Sodium (Na) | mg/L | 0.004 | M‒W | 0.143 | 0.002 | M‒W | 0.165 | 0.005 | M‒W | 0.165 | 0.004 | M‒W | 0.165 |
| Nickel (Ni) | mg/L | <0.001 | M‒W | 0.012 | <0.001 | M‒W | 0.182 | <0.001 | M‒W | 0.016 | <0.001 | M‒W | 0.574 |
| Zinc (Zn) | mg/L | <0.001 | M‒W | 0.006 | <0.001 | M‒W | <0.001 | 0.001 | M‒W | 0.503 | 0.001 | M‒W | 0.538 |

^a^ HRT, hydraulic retention time

^b^ T9, treatment system with *Phragmites australis* only

^c^ T10, treatment system with *P. australis* and ochre pellets

^d^ T11, treatment system without *P. australis* or ochre pellets

^e^ T12, treatment system with ochre pellets only

^f^ Shapiro‒Wilk (check for normality), normally distributed data, if p>0.05 using T‒test, and non‒normally distributed data, if p<0.05 using Mann‒Whitney U‒test; p value (M‒W), significant difference, if p<0.05, and not significant, if p>0.05

^g^ NTU, nephelometric turbidity unit

**Online Resource 4** (Continued)

| d) LC‒SGW: Comparisons at 7‒day HRT^a^ (inflow & outflow) | | | | | | | | | | | | | |
| --- | --- | --- | --- | --- | --- | --- | --- | --- | --- | --- | --- | --- | --- |
|  | | Inflow & T13^b^ | | | Inflow & T14^c^ | | | Inflow & T15^d^ | | | Inflow & T16^e^ | | |
| Parameter | Unit | Shapiro‒Wilk (p value) | Statistical test^f^ | Significance (p value) | Shapiro‒Wilk (p value) | Statistical test^f^ | Significance (p value) | Shapiro‒Wilk (p value) | Statistical test^f^ | Significance (p value) | Shapiro‒Wilk (p value) | Statistical test^f^ | Significance (p value) |
| pH | ‒ | <0.001 | M‒W | 0.547 | <0.001 | M‒W | <0.001 | 0.005 | M‒W | <0.001 | <0.001 | M‒W | <0.001 |
| Redox potential | mV | 0.005 | M‒W | 0.140 | 0.005 | M‒W | <0.001 | 0.005 | M‒W | <0.001 | 0.005 | M‒W | <0.001 |
| Turbidity | NTU^g^ | 0.105 | T‒test | 0.007 | 0.005 | M‒W | 0.811 | 0.008 | M‒W | <0.001 | 0.005 | M‒W | <0.001 |
| Total suspended solids | mg/L | 0.002 | M‒W | <0.001 | <0.001 | M‒W | 0.847 | <0.001 | M‒W | <0.001 | 0.002 | M‒W | <0.001 |
| Electronic conductivity | µS/cm | <0.001 | M‒W | 0.765 | <0.001 | M‒W | <0.001 | <0.001 | M‒W | 0.013 | <0.001 | M‒W | <0.001 |
| Dissolved oxygen | mg/L | 0.003 | M‒W | <0.001 | 0.001 | M‒W | <0.001 | <0.001 | M‒W | 0.005 | <0.001 | M‒W | 0.068 |
| Colour | Pa/Co | 0.173 | T‒test | <0.001 | <0.001 | M‒W | 0.230 | 0.245 | T‒test | <0.001 | 0.100 | T‒test | 0.002 |
| Temperature | °C | 0.002 | M‒W | 0.009 | 0.001 | M‒W | 0.783 | 0.002 | M‒W | 0.001 | 0.002 | M‒W | 0.392 |
| Biochemical oxygen demand | mg/L | <0.001 | M‒W | 0.001 | <0.001 | M‒W | <0.001 | <0.001 | M‒W | <0.001 | <0.001 | M‒W | <0.001 |
| Chemical oxygen demand | mg/L | <0.001 | M‒W | 0.029 | <0.001 | M‒W | 0.506 | <0.001 | M‒W | <0.001 | <0.001 | M‒W | 0.001 |
| Ammonia‒nitrogen | mg/L | <0.001 | M‒W | 0.001 | <0.001 | M‒W | 0.169 | <0.001 | M‒W | <0.001 | <0.001 | M‒W | 0.019 |
| Nitrate‒nitrogen | mg/L | <0.001 | M‒W | 0.408 | <0.001 | M‒W | 0.001 | <0.001 | M‒W | 0.201 | <0.001 | M‒W | <0.001 |
| Ortho‒phosphate‒phosphorus | mg/L | <0.001 | M‒W | <0.001 | <0.001 | M‒W | <0.001 | <0.001 | M‒W | 0.606 | <0.001 | M‒W | <0.001 |
| Aluminium (Al) | mg/L | <0.001 | M‒W | <0.001 | <0.001 | M‒W | 0.378 | <0.001 | M‒W | 0.321 | <0.001 | M‒W | 0.025 |
| Boron (B) | mg/L | <0.001 | M‒W | <0.001 | <0.001 | M‒W | <0.001 | <0.001 | M‒W | <0.001 | <0.001 | M‒W | <0.001 |
| Calcium (Ca) | mg/L | 0.001 | M‒W | <0.001 | <0.001 | M‒W | <0.001 | 0.079 | T‒test | 0.201 | <0.001 | M‒W | <0.001 |
| Cadmium (Cd) | mg/L | <0.001 | M‒W | 0.001 | <0.001 | M‒W | <0.001 | <0.001 | M‒W | 0.290 | <0.001 | M‒W | 0.050 |
| Chromium (Cr) | mg/L | <0.001 | M‒W | 0.717 | <0.001 | M‒W | 0.945 | <0.001 | M‒W | 0.360 | <0.001 | M‒W | 0.738 |
| Copper (Cu) | mg/L | <0.001 | M‒W | <0.001 | <0.001 | M‒W | <0.001 | <0.001 | M‒W | <0.001 | <0.001 | M‒W | <0.001 |
| Iron (Fe) | mg/L | <0.001 | M‒W | 0.007 | <0.001 | M‒W | <0.001 | <0.001 | M‒W | 0.444 | <0.001 | M‒W | <0.001 |
| Potassium (K) | mg/L | 0.151 | T‒test | <0.001 | <0.001 | M‒W | 1.000 | 0.334 | T‒test | 0.054 | <0.001 | M‒W | 1.000 |
| Magnesium (Mg) | mg/L | <0.001 | M‒W | 0.006 | <0.001 | M‒W | <0.001 | <0.001 | M‒W | 0.159 | <0.001 | M‒W | <0.001 |
| Manganese (Mn) | mg/L | <0.001 | M‒W | <0.001 | <0.001 | M‒W | <0.001 | <0.001 | M‒W | <0.001 | <0.001 | M‒W | <0.001 |
| Sodium (Na) | mg/L | 0.001 | M‒W | 0.316 | 0.011 | M‒W | 0.396 | 0.012 | M‒W | 0.165 | 0.028 | M‒W | 1.000 |
| Nickel (Ni) | mg/L | <0.001 | M‒W | <0.001 | <0.001 | M‒W | <0.001 | <0.001 | M‒W | <0.001 | <0.001 | M‒W | <0.001 |
| Zinc (Zn) | mg/L | <0.001 | M‒W | 0.045 | <0.001 | M‒W | <0.001 | <0.001 | M‒W | 0.328 | <0.001 | M‒W | 0.010 |

^a^ HRT, hydraulic retention time

^b^ T13, treatment system with *Phragmites australis* only

^c^ T14, treatment system with *P. australis* and ochre pellets

^d^ T15, treatment system without *P. australis* or ochre pellets

^e^ T16, treatment system with ochre pellets only

^f^ Shapiro‒Wilk (check for normality), normally distributed data, if p>0.05 using T‒test, and non‒normally distributed data, if p<0.05 using Mann‒Whitney U‒test; p value (M‒W), significant difference, if p<0.05, and not significant, if p>0.05

^g^ NTU, nephelometric turbidity unit
