# Supplementary material for: Phytoremediation performance of floating treatment wetlands with pelletized mine water sludge for synthetic greywater treatment
Source: J Environ Health Sci Eng. 2019 Apr 18;17(2):581–608. doi: 10.1007/s40201-019-00372-z (PMC6985343; doi:10.1007/s40201-019-00372-z)
Supplement: Supplementary file 6 — (DOCX 107 kb) [file 40201_2019_372_MOESM6_ESM.docx]

**Phytoremediation performance of floating treatment wetlands with pelletized mine water sludge for synthetic greywater treatment**

*Journal of Environmental Health Science and Engineering*

**Suhail N. Abed, Suhad A. Almuktar, Miklas Scholz**

Corresponding author: Miklas Scholz

Civil Engineering Research Group, School of Computing, Science and Engineering, The University of Salford, Newton Building, Salford M5 4WT, England, United Kingdom.

Division of Water Resources Engineering, Department of Building and Environmental Technology, Faculty of Engineering, Lund University, P.O. Box 118, 221 00 Lund, Sweden.

Department of Civil Engineering Science, School of Civil Engineering and the Built Environment, University of Johannesburg, Kingsway Campus, PO Box 524, Aukland Park 2006, Johannesburg, South Africa

E‒mail address: [miklas.scholz@tvrl.lth.se](mailto:miklas.scholz@tvrl.lth.se)

**Online Resource 6** The accumulated trace element concentrations in cement‒ochre pellets showing *a)* effects of *Phragmites australis* and hydraulic retention time (HRT) on high concentration synthetic greywaters (HC‒SGW) concentrations, *b)* effects of *P. australis* and HRT on HC‒SGW accumulation percentages, *c)* effects of *P. australis* and HRT on low concentration synthetic greywater (LC‒SGW) concentrations, *d)* effects of *P. australis* and HRT on treatment HC‒SGW accumulation percentages, *e)* effects of pollutant loads of SGW on adsorbed concentrations, and *f )* effects of pollutant loads of SGW on accumulation percentages
