# Supplementary material for: Phytoremediation performance of floating treatment wetlands with pelletized mine water sludge for synthetic greywater treatment
Source: J Environ Health Sci Eng. 2019 Apr 18;17(2):581–608. doi: 10.1007/s40201-019-00372-z (PMC6985343; doi:10.1007/s40201-019-00372-z)
Supplement: Supplementary file 7 — (DOCX 22.6 kb) [file 40201_2019_372_MOESM7_ESM.docx]

**Phytoremediation performance of floating treatment wetlands with pelletized mine water sludge for synthetic greywater treatment**

*Journal of Environmental Health Science and Engineering*

**Suhail N. Abed, Suhad A. Almuktar, Miklas Scholz**

Corresponding author: Miklas Scholz

Civil Engineering Research Group, School of Computing, Science and Engineering, The University of Salford, Newton Building, Salford M5 4WT, England, United Kingdom.

Division of Water Resources Engineering, Department of Building and Environmental Technology, Faculty of Engineering, Lund University, P.O. Box 118, 221 00 Lund, Sweden.

Department of Civil Engineering Science, School of Civil Engineering and the Built Environment, University of Johannesburg, Kingsway Campus, PO Box 524, Aukland Park 2006, Johannesburg, South Africa

E‒mail address: miklas.scholz@tvrl.lth.se

**Online Resource 7** Trace elements (mg/kg) accumulated in cement‒ochre pellets after the treatment period of high concentration synthetic greywater (HC‒SGW) and low concentration synthetic greywater (LC‒SGW) in mesocosm‒scale treatment systems (T) at *(a)* 2‒day HRT and *(b)* 7‒day HRT

| (a) 2‒day HRT^a^ | | | | | | | | | | | | |
| --- | --- | --- | --- | --- | --- | --- | --- | --- | --- | --- | --- | --- |
| HC‒SGW | | | | | | | | | | | | |
| Element | Cement‒ochre pellets in T2^b^ | | | | | | Cement‒ochre pellets in T4^c^ | | | | | |
|  | n^d^ | Mean | SD^e^ | Min^f^ | Max^g^ | Accumulation (%) | n^d^ | Mean | SD^e^ | Min^f^ | Max^g^ | Accumulation (%) |
| Aluminium | 18 | 15453.0 | 1226.75 | 13359.0 | 17573.7 | 12.8 | 18 | 14589.7 | 1153.27 | 12352.2 | 16197.7 | 6.5 |
| Boron | 15 | 95.3 | 16.42 | 73.3 | 127.9 | 158.1 | 15 | 82.0 | 17.80 | 55.5 | 115.9 | 122.1 |
| Calcium | 21 | 225571.9 | 12870.26 | 201974.0 | 248470.8 | -16.0 | 21 | 214795.4 | 12783.36 | 190833.2 | 231786.0 | -20.0 |
| Cadmium | 12 | 183.3 | 59.18 | 123.9 | 279.3 | 4005.7 | 14 | 237.0 | 89.84 | 26.4 | 334.2 | 5208.6 |
| Chromium | 27 | 286.0 | 228.90 | 52.0 | 665.8 | 501.5 | 27 | 121.5 | 74.45 | 41.3 | 277.4 | 155.6 |
| Copper | 27 | 156.1 | 80.44 | 69.9 | 331.6 | 108.7 | 27 | 118.7 | 69.27 | 52.8 | 279.8 | 58.7 |
| Iron | 24 | 250273.6 | 16905.83 | 217753.6 | 277509.2 | 11.43 | 24 | 235900.8 | 18729.34 | 198789.6 | 263774.4 | 5.0 |
| Magnesium | 27 | 8039.4 | 757.51 | 6064.0 | 9071.8 | 65.5 | 27 | 7999.2 | 755.80 | 5994.7 | 8759.9 | 64.6 |
| Manganese | 27 | 2493.9 | 195.09 | 2152.1 | 2963.2 | 14.8 | 27 | 2443.6 | 230.23 | 2024.0 | 2894.8 | 12.5 |
| Nickel | 12 | 18.7 | 11.36 | 5.3 | 43.6 | 20.5 | 21 | 28.2 | 18.74 | 2.4 | 58.1 | 81.8 |
| Zinc | 24 | 593.4 | 157.11 | 367.7 | 1019.2 | 32.5 | 24 | 546.0 | 160.59 | 334.6 | 917.9 | 22.0 |
| LC‒SGW | | | | | | | | | | | | |
| Element | Cement‒ochre pellets in T6^h^ | | | | | | Cement‒ochre pellets in T8^i^ | | | | | |
|  | n^d^ | Mean | SD^e^ | Min^f^ | Max^g^ | Accumulation (%) | n^d^ | Mean | SD^e^ | Min^f^ | Max^g^ | Accumulation (%) |
| Aluminium | 18 | 16651.9 | 1307.90 | 14055.0 | 18726.7 | 21.6 | 18 | 16867.7 | 1114.87 | 14944.1 | 18942.3 | 23.2 |
| Boron | 15 | 43.9 | 18.62 | 23.6 | 77.7 | 19.0 | 15 | 38.9 | 16.62 | 22.5 | 69.5 | 5.3 |
| Calcium | 21 | 230721.7 | 9753.74 | 211039.2 | 247784.0 | -14.1 | 21 | 247556.5 | 15332.50 | 220230.0 | 276971.6 | -7.8 |
| Cadmium | 16 | 8.2 | 8.20 | 0.0 | 22.9 | 83.9 | 15 | 10.7 | 21.03 | 0.0 | 82.7 | 139.6 |
| Chromium | 27 | 49.7 | 26.48 | 17.3 | 110.9 | 4.6 | 27 | 58.4 | 25.40 | 23.8 | 110.2 | 22.8 |
| Copper | 27 | 103.4 | 78.84 | 30.2 | 271.2 | 38.3 | 27 | 117.1 | 84.00 | 17.6 | 319.4 | 56.6 |
| Iron | 24 | 260408.6 | 14308.43 | 233275.2 | 284728.0 | 15.9 | 24 | 246941.2 | 17821.97 | 210530.0 | 273850.4 | 9.9 |
| Magnesium | 27 | 6787.9 | 634.83 | 5096.2 | 7512.0 | 39.7 | 27 | 6586.0 | 572.12 | 5121.9 | 7399.4 | 35.5 |
| Manganese | 27 | 2544.0 | 175.72 | 2276.7 | 3005.5 | 17.1 | 27 | 2424.3 | 168.58 | 2160.8 | 2839.4 | 11.6 |
| Nickel | 12 | 16.8 | 9.17 | 3.5 | 32.1 | 8.0 | 14 | 21.3 | 12.16 | 0.1 | 43.9 | 37.1 |
| Zinc | 24 | 589.5 | 208.77 | 358.1 | 1053.9 | 31.7 | 24 | 589.0 | 188.00 | 436.6 | 1059.1 | 31.5 |

^a^ HRT, hydraulic retention time

^b^ T2, HC‒SGW treatment systems with ochre pellets and floating *Phragmites australis*

^c^ T4, HC‒SGW treatment systems with ochre pellets only

^d^ n, number of tested samples

^e^ SD, standard deviation

^f^ Min, minimum

^g^ Max, maximum

^h^ T6, LC‒SGW treatment systems with ochre pellets and floating *Phragmites australis*

^i^ T8, LC‒SGW treatment systems with ochre pellets only

**Online Resource 7** (Continued)

| (b) 7‒day HRT^a^ | | | | | | | | | | | | |
| --- | --- | --- | --- | --- | --- | --- | --- | --- | --- | --- | --- | --- |
| HC‒SGW | | | | | | | | | | | | |
| Element | Cement‒ochre pellets in T10^b^ | | | | | | Cement‒ochre pellets in T12^c^ | | | | | |
|  | n^d^ | Mean | SD^e^ | Min^f^ | Max^g^ | Accumulation (%) | n^d^ | Mean | SD^e^ | Min^f^ | Max^g^ | Accumulation (%) |
| Aluminium | 18 | 14775.3 | 1203.45 | 12561.2 | 16514.0 | 7.9 | 18 | 16130.6 | 969.31 | 14621.8 | 17984.0 | 17.8 |
| Boron | 15 | 97.3 | 15.87 | 75.1 | 127.5 | 163.5 | 15 | 97.4 | 15.84 | 79.2 | 124.4 | 163.7 |
| Calcium | 21 | 210908.8 | 15683.93 | 182122.4 | 234072.0 | -21.5 | 21 | 242023.8 | 11280.09 | 217711.6 | 258784.8 | -9.9 |
| Cadmium | 12 | 152.1 | 42.83 | 65.7 | 203.7 | 3306.4 | 12 | 265.1 | 50.32 | 169.8 | 356.4 | 5838.1 |
| Chromium | 27 | 88.7 | 38.06 | 42.7 | 193.4 | 86.6 | 27 | 95.8 | 34.21 | 51.5 | 197.2 | 101.5 |
| Copper | 27 | 133.3 | 76.88 | 8.2 | 335.7 | 78.22 | 27 | 130.5 | 80.23 | 42.4 | 329.5 | 74.5 |
| Iron | 24 | 246726.8 | 14898.61 | 216570.8 | 269527.6 | 9.8 | 24 | 243388.2 | 16023.00 | 210822.0 | 265205.2 | 8.4 |
| Magnesium | 27 | 7898.1 | 751.94 | 5926.2 | 8885.0 | 62.5 | 27 | 8194.8 | 712.29 | 6240.2 | 8976.9 | 68.7 |
| Manganese | 27 | 2508.1 | 172.89 | 2249.0 | 2944.5 | 15.5 | 27 | 2522.1 | 255.90 | 2104.0 | 3050.9 | 16.1 |
| Nickel | 18 | 26.4 | 19.06 | 1.4 | 66.8 | 69.9 | 20 | 30.7 | 20.60 | 2.3 | 73.2 | 97.8 |
| Zinc | 24 | 577.2 | 165.32 | 421.4 | 1005.7 | 28.9 | 24 | 626.7 | 189.38 | 441.2 | 1032.5 | 40.0 |
| LC‒SGW | | | | | | | | | | | | |
| Element | Cement‒ochre pellets in ^T14h^ | | | | | | Cement‒ochre pellets in T16^i^ | | | | | |
|  | n^d^ | Mean | SD^e^ | Min^f^ | Max^g^ | Accumulation (%) | n^d^ | Mean | SD^e^ | Min^f^ | Max^g^ | Accumulation (%) |
| Aluminium | 18 | 16492.5 | 903.51 | 15021.5 | 17988.1 | 20.4 | 18 | 16874.5 | 914.04 | 15309.2 | 18198.2 | 23.2 |
| Boron | 15 | 52.9 | 17.21 | 33.3 | 83.2 | 43.3 | 15 | 53.1 | 17.16 | 35.4 | 82.3 | 43.9 |
| Calcium | 21 | 229105.5 | 11801.71 | 208161.6 | 251568.0 | -14.7 | 21 | 242354.2 | 9066.79 | 224942.0 | 256550.4 | -9.7 |
| Cadmium | 18 | 80.4 | 115.69 | 0.0 | 298.6 | 1701.8 | 18 | 11.8 | 10.14 | 0.0 | 26.2 | 164.3 |
| Chromium | 27 | 139.9 | 95.20 | 25.8 | 325.5 | 194.3 | 27 | 68.1 | 33.26 | 29.5 | 153.0 | 43.2 |
| Copper | 27 | 114.8 | 75.64 | 35.3 | 282.2 | 53.5 | 27 | 106.9 | 78.81 | 40.0 | 288.2 | 42.9 |
| Iron | 24 | 251949.5 | 16346.18 | 221098.8 | 278889.6 | 12.2 | 24 | 254510.0 | 13227.47 | 230163.2 | 274306.4 | 13.3 |
| Magnesium | 27 | 6613.5 | 543.72 | 5177.4 | 7299.5 | 36.1 | 27 | 6492.4 | 511.48 | 5170.8 | 6962.6 | 33.6 |
| Manganese | 27 | 2461.8 | 171.87 | 2239.3 | 2892.3 | 13.4 | 27 | 2529.9 | 144.24 | 2302.0 | 2855.0 | 16.5 |
| Nickel | 11 | 22.2 | 11.61 | 4.9 | 42.2 | 43.2 | 17 | 29.0 | 20.60 | 0.4 | 59.2 | 86.6 |
| Zinc | 24 | 576.3 | 187.77 | 402.4 | 1054.8 | 28.7 | 24 | 578.2 | 175.57 | 449.2 | 1013.4 | 29.1 |

^a^ HRT, hydraulic retention time

^b^ T10, HC‒SGW treatment systems with ochre pellets combined with floating *Phragmites australis*

^c^ T12, HC‒SGW treatment systems with ochre pellets only

^d^ n, number of tested samples

^e^ SD, standard deviation

^f^ Min, minimum

^g^ Max, maximum

^h^ T14, LC‒SGW treatment systems with ochre pellets combined with floating *Phragmites australis*

^i^ T16, LC‒SGW treatment systems with ochre pellets only
