# Supplementary material for: Phytoremediation performance of floating treatment wetlands with pelletized mine water sludge for synthetic greywater treatment
Source: J Environ Health Sci Eng. 2019 Apr 18;17(2):581–608. doi: 10.1007/s40201-019-00372-z (PMC6985343; doi:10.1007/s40201-019-00372-z)
Supplement: Supplementary file 9 — (DOCX 21.6 kb) [file 40201_2019_372_MOESM9_ESM.docx]

**Phytoremediation performance of floating treatment wetlands with pelletized mine water sludge for synthetic greywater treatment**

*Journal of Environmental Health Science and Engineering*

**Suhail N. Abed, Suhad A. Almuktar, Miklas Scholz**

Corresponding author: Miklas Scholz

Civil Engineering Research Group, School of Computing, Science and Engineering, The University of Salford, Newton Building, Salford M5 4WT, England, United Kingdom.

Division of Water Resources Engineering, Department of Building and Environmental Technology, Faculty of Engineering, Lund University, P.O. Box 118, 221 00 Lund, Sweden.

Department of Civil Engineering Science, School of Civil Engineering and the Built Environment, University of Johannesburg, Kingsway Campus, PO Box 524, Aukland Park 2006, Johannesburg, South Africa

E‒mail address: miklas.scholz@tvrl.lth.se

**Online Resource 9** Significance values of statistical analysis for accumulated elements (mg/kg) in *P. australis* tissue in the floating treatment systems (T) concerning high concentration (HC) and low concentration (LC) synthetic greywaters (SGW) to assess *(a)* the effect of ochre pellets on element accumulation in *P. australis* tissues, *(b)* the effect of HRT on element accumulation in *P. australis* tissues, and *(c)* the effect of pollutant concentrations on element accumulation in *P. australis* tissues

| a) Effect of ochre pellets on trace element accumulation in *P. australis* | | | | | | | | | | | | | | | | | | | | | | | | |
| --- | --- | --- | --- | --- | --- | --- | --- | --- | --- | --- | --- | --- | --- | --- | --- | --- | --- | --- | --- | --- | --- | --- | --- | --- |
|  | 2‒day HRT^a^ | | | | | | | | | | | | 7‒day HRT^a^ | | | | | | | | | | | |
|  | HC‒SGW (T1^b^ & T2^c^) | | | | | | LC‒SGW (T5^d^ & T6^e^) | | | | | | HC‒SGW (T9^f^ & T10^g^) | | | | | | LC‒SGW (T13^h^ & T14^i^) | | | | | |
| Parameter | Shapiro‒Wilk (p value) | | Statistical test^j^ | | Significance (p value) | | Shapiro‒Wilk (p value) | | Statistical test^j^ | | Significance (p value) | | Shapiro‒Wilk (p value) | | Statistical test^j^ | | Significance (p value) | | Shapiro‒Wilk (p value) | | Statistical test^j^ | | Significance (p value) | |
| Aluminium | <0.001 | | M‒W | | <0.001 | | <0.001 | | M‒W | | <0.001 | | <0.001 | | M‒W | | <0.001 | | <0.001 | | M‒W | | <0.001 | |
| Boron | 0.264 | | T‒test | | 0.027 | | 0.001 | | M‒W | | 0.015 | | 0.073 | | T‒test | | <0.001 | | <0.001 | | M‒W | | 0.029 | |
| Calcium | <0.001 | | M‒W | | <0.001 | | <0.001 | | M‒W | | <0.001 | | <0.001 | | M‒W | | <0.001 | | <0.001 | | M‒W | | <0.001 | |
| Cadmium | 0.001 | | M‒W | | <0.001 | | <0.001 | | M‒W | | <0.001 | | <0.001 | | M‒W | | <0.001 | | <0.001 | | M‒W | | <0.001 | |
| Chromium | <0.001 | | M‒W | | <0.001 | | <0.001 | | M‒W | | <0.001 | | <0.001 | | M‒W | | <0.001 | | <0.001 | | M‒W | | <0.001 | |
| Copper | <0.001 | | M‒W | | <0.001 | | <0.001 | | M‒W | | <0.001 | | <0.001 | | M‒W | | <0.001 | | <0.001 | | M‒W | | <0.001 | |
| Iron | <0.001 | | M‒W | | <0.001 | | <0.001 | | M‒W | | <0.001 | | <0.001 | | M‒W | | <0.001 | | <0.001 | | M‒W | | <0.001 | |
| Magnesium | 0.008 | | M‒W | | <0.001 | | <0.001 | | M‒W | | <0.001 | | 0.079 | | T‒test | | <0.001 | | <0.001 | | M‒W | | <0.001 | |
| Manganese | <0.001 | | M‒W | | <0.001 | | <0.001 | | M‒W | | <0.001 | | <0.001 | | M‒W | | <0.001 | | <0.001 | | M‒W | | <0.001 | |
| Sodium | 0.187 | | T‒test | | 0.128 | | 0.043 | | M‒W | | <0.001 | | 0.003 | | M‒W | | <0.001 | | 0.004 | | M‒W | | <0.001 | |
| Nickel | 0.001 | | M‒W | | <0.001 | | <0.001 | | M‒W | | <0.001 | | <0.001 | | M‒W | | <0.001 | | <0.001 | | M‒W | | <0.001 | |
| Zinc | <0.001 | | M‒W | | <0.001 | | <0.001 | | M‒W | | <0.001 | | <0.001 | | M‒W | | <0.001 | | 0.001 | | M‒W | | <0.001 | |
| b) Effect of HRT^a^ on trace element accumulation in *P. australis* | | | | | | | | | | | | | | | | | | | | | | | | |
|  | HC‒SGW | | | | | | | | | LC‒SGW | | | | | | | | | | Control wetlands | | | | |
|  | T1^b^ & T9^f^ | | | | | T2^c^ & T10^g^ | | | | T5^d^ & T13^h^ | | | | | | T6^e^ & T14^i^ | | | | C1^k^ & C3^l^ | | | | |
| Parameter | Shapiro‒Wilk (p value) | Statistical test^j^ | | Significance (p value) | | Shapiro‒Wilk (p value) | | Statistical test^j^ | Significance (p value) | Shapiro‒Wilk (p value) | | Statistical test^j^ | | Significance (p value) | | Shapiro‒Wilk (p value) | Statistical test^j^ | Significance (p value) | | Shapiro‒Wilk (p value) | | Statistical test^j^ | | Significance (p value) |
| Aluminium | <0.001 | M‒W | | <0.001 | | <0.001 | | M‒W | 0.409 | <0.001 | | M‒W | | <0.001 | | <0.001 | M‒W | <0.001 | | 0.009 | | M‒W | | <0.001 |
| Boron | 0.022 | M‒W | | 0.917 | | 0.040 | | M‒W | <0.001 | <0.001 | | M‒W | | 0.724 | | 0.002 | M‒W | 0.290 | | <0.001 | | M‒W | | 0.120 |
| Calcium | 0.290 | T‒test | | 0.956 | | <0.001 | | M‒W | <0.001 | 0.088 | | T‒test | | 0.062 | | <0.001 | M‒W | <0.001 | | 0.084 | | T‒test | | <0.001 |
| Cadmium | 0.015 | M‒W | | <0.001 | | 0.014 | | M‒W | 0.681 | <0.001 | | M‒W | | <0.001 | | 0.137 | T‒test | 0.933 | | 0.004 | | M‒W | | 0.194 |
| Chromium | <0.001 | M‒W | | <0.001 | | <0.001 | | M‒W | 0.036 | <0.001 | | M‒W | | <0.001 | | 0.004 | M‒W | <0.001 | | 0.001 | | M‒W | | <0.001 |
| Copper | <0.001 | M‒W | | <0.001 | | 0.001 | | M‒W | 0.088 | <0.001 | | M‒W | | <0.001 | | <0.001 | M‒W | 0.008 | | <0.001 | | M‒W | | 0.036 |
| Iron | <0.001 | M‒W | | <0.001 | | <0.001 | | M‒W | <0.001 | <0.001 | | M‒W | | <0.001 | | 0.029 | M‒W | <0.001 | | <0.001 | | M‒W | | <0.001 |
| Magnesium | 0.016 | M‒W | | <0.001 | | <0.001 | | M‒W | <0.001 | 0.075 | | T‒test | | <0.001 | | <0.001 | M‒W | 0.095 | | 0.047 | | M‒W | | <0.001 |
| Manganese | <0.001 | M‒W | | 0.574 | | <0.001 | | M‒W | <0.001 | <0.001 | | M‒W | | <0.001 | | <0.001 | M‒W | 0.392 | | <0.001 | | M‒W | | <0.001 |
| Sodium | 0.526 | T‒test | | <0.001 | | 0.007 | | M‒W | <0.001 | 0.139 | | T‒test | | <0.001 | | <0.001 | M‒W | <0.001 | | 0.020 | | M‒W | | <0.001 |
| Nickel | <0.001 | M‒W | | <0.001 | | <0.001 | | M‒W | 0.023 | 0.001 | | M‒W | | <0.001 | | <0.001 | M‒W | 0.883 | | <0.001 | | M‒W | | 0.040 |
| Zinc | 0.002 | M‒W | | <0.001 | | <0.001 | | M‒W | 0.001 | <0.001 | | M‒W | | <0.001 | | <0.001 | M‒W | 0.332 | | 0.001 | | M‒W | | 0.018 |

^a^ HRT, hydraulic retention time

^b^ T1, HC‒SGW treatment systems 2-day with only floating *P. australis*

^c^ T2, HC‒SGW treatment systems 2-day with floating *P. australis* and ochre pellets

^d^ T5, LC-SGW treatment systems 2-day with only floating *P. australis*

^e^ T6, LC-SGW treatment systems 2-day with floating *P. australis* and ochre pellets

^f^ T9, HC-SGW treatment systems 7-day with only floating *P. australis*

^g^ T10, HC-SGW treatment systems 7-day with floating *P. australis* and ochre pellets

^h^ T13, LC-SGW treatment systems 7-day with only floating *P. australis*

^i^ T14, LC-SGW treatment systems 7-day with floating *P. australis* and ochre pellets

^j^ Shapiro‒Wilk (check for normality), normally distributed data, if p > 0.05 using T‒test, and non‒normally distributed data, if p < 0.05 using Mann‒Whitney U‒test; p value, significantly different, if p < 0.05, and not significantly different, if p > 0.05; and M‒W, Mann‒Whitney U‒test

^k^ C1, control wetland containing floating *P. australis* in tap water at 2‒day HRT

^l^ C3, control wetland containing floating *P. australis* in tap water at 7‒day HRT

**Online Resource 9** (Continued)

| c) Effect of pollutant concentrations on trace element accumulation in *P. australis*. | | | | | | | | | | | | |
| --- | --- | --- | --- | --- | --- | --- | --- | --- | --- | --- | --- | --- |
|  | 2‒day HRT^a^ | | | | | | 7‒day HRT^a^ | | | | | |
|  | T1^b^ & T5^c^ | | | T2^d^ & T6^e^ | | | T9^f^ & T13^g^ | | | T10^h^ & T14^i^ | | |
| Parameter | Shapiro‒Wilk (p value) | Statistical test^j^ | Significance (p value) | Shapiro‒Wilk (p value) | Statistical test^j^ | Significance (p value) | Shapiro‒Wilk (p value) | Statistical test^j^ | Significance (p value) | Shapiro‒Wilk (p value) | Statistical test^j^ | Significance (p value) |
| Aluminium | <0.001 | M‒W | <0.001 | <0.001 | M‒W | <0.001 | <0.001 | M‒W | <0.001 | 0.001 | M‒W | <0.001 |
| Boron | 0.303 | T‒test | <0.001 | 0.006 | M‒W | <0.001 | 0.116 | T‒test | <0.001 | 0.009 | M‒W | 0.004 |
| Calcium | 0.065 | T‒test | <0.001 | <0.001 | M‒W | <0.001 | 0.101 | T‒test | <0.001 | 0.001 | M‒W | <0.001 |
| Cadmium | <0.001 | M‒W | <0.001 | <0.001 | M‒W | <0.001 | <0.001 | M‒W | <0.001 | <0.001 | M‒W | <0.001 |
| Chromium | <0.001 | M‒W | <0.001 | <0.001 | M‒W | <0.001 | <0.001 | M‒W | <0.001 | <0.001 | M‒W | <0.001 |
| Copper | <0.001 | M‒W | <0.001 | <0.001 | M‒W | <0.001 | <0.001 | M‒W | <0.001 | <0.001 | M‒W | <0.001 |
| Iron | <0.001 | M‒W | <0.001 | <0.001 | M‒W | <0.001 | <0.001 | M‒W | <0.001 | <0.001 | M‒W | <0.001 |
| Magnesium | 0.003 | M‒W | 0.001 | <0.001 | M‒W | <0.001 | <0.001 | M‒W | <0.001 | <0.001 | M‒W | <0.001 |
| Manganese | 0.003 | M‒W | 0.001 | <0.001 | M‒W | <0.001 | <0.001 | M‒W | <0.001 | <0.001 | M‒W | <0.001 |
| Sodium | <0.001 | M‒W | <0.001 | <0.001 | M‒W | <0.001 | <0.001 | M‒W | <0.001 | 0.012 | M‒W | <0.001 |
| Nickel | 0.021 | M‒W | <0.001 | <0.001 | M‒W | 0.001 | <0.001 | M‒W | <0.001 | <0.001 | M‒W | <0.001 |
| Zinc | 0.002 | M‒W | <0.001 | <0.001 | M‒W | <0.001 | <0.001 | M‒W | <0.001 | <0.001 | M‒W | <0.001 |

^a^ HRT, hydraulic retention time

^b^ T1, HC‒SGW treatment systems 2-day with only floating *P. australis*

^c^ T5, LC-SGW treatment systems 2-day with only floating *P. australis*

^d^ T2, HC‒SGW treatment systems 2-day with floating *P. australis* and ochre pellets

^e^ T6, LC-SGW treatment systems 2-day with floating *P. australis* and ochre pellets

^f^ T9, HC-SGW treatment systems 7-day with only floating *P. australis*

^g^ T13, LC-SGW treatment systems 7-day with only floating *P. australis*

^h^ T10, HC-SGW treatment systems 7-day with floating *P. australis* and ochre pellets

^i^ T14, LC-SGW treatment systems 7-day with floating *P. australis* and ochre pellets

^j^ Shapiro‒Wilk (check for normality), normally distributed data, if p > 0.05 using T‒test, and non‒normally distributed data, if p < 0.05 using Mann‒Whitney U‒test; p value, significantly different, if p < 0.05, and not significantly different, if p > 0.05; and M‒W, Mann‒Whitney U‒test
