# Supplementary material for: Phytoremediation performance of floating treatment wetlands with pelletized mine water sludge for synthetic greywater treatment
Source: J Environ Health Sci Eng. 2019 Apr 18;17(2):581–608. doi: 10.1007/s40201-019-00372-z (PMC6985343; doi:10.1007/s40201-019-00372-z)
Supplement: Supplementary file 10 — (DOCX 23.8 kb) [file 40201_2019_372_MOESM10_ESM.docx]

**Phytoremediation performance of floating treatment wetlands with pelletized mine water sludge for synthetic greywater treatment**

*Journal of Environmental Health Science and Engineering*

**Suhail N. Abed, Suhad A. Almuktar, Miklas Scholz**

Corresponding author: Miklas Scholz

Civil Engineering Research Group, School of Computing, Science and Engineering, The University of Salford, Newton Building, Salford M5 4WT, England, United Kingdom.

Division of Water Resources Engineering, Department of Building and Environmental Technology, Faculty of Engineering, Lund University, P.O. Box 118, 221 00 Lund, Sweden.

Department of Civil Engineering Science, School of Civil Engineering and the Built Environment, University of Johannesburg, Kingsway Campus, PO Box 524, Aukland Park 2006, Johannesburg, South Africa

E‒mail address: miklas.scholz@tvrl.lth.se

**Online Resource 10** Detected trace element concentrations (mg/kg) accumulated in *P. australis* leaves, stems, and rhizomes at the end of the experiment of floating treatment wetlands (C and T) after treatment of; *(a)* high pollutant concentrations (HC‒SGW), and *(b)* low pollutant concentrations synthetic greywater (LC‒SGW)

| a) HC‒SGW | | | | | | | | | | | | | | | | | | |
| --- | --- | --- | --- | --- | --- | --- | --- | --- | --- | --- | --- | --- | --- | --- | --- | --- | --- | --- |
|  | 2‒day hydraulic retention time (HRT)* | | | | | | | | | | | | | | | | | |
| Element | *P. australis* in C1^a^ | | | | | | *P. australis* in T1^b^ | | | | | | *P. australis* in T2^c^ | | | | | |
|  | Leaf | | Stem | | Rhizome | | Leaf | | Stem | | Rhizome | | Leaf | | Stem | | | Rhizome |
| Aluminium | 25.8±22.51 (48) | | 6.3±13.51 (24) | | 25.9±26.60 (24) | | 108.5±69.22 (49) | | 36.2±20.01 (24) | | 4155.0±736.04 (24) | | 116.0±107.16 (50) | | 262.4±86.51 (24) | | | 394.6±133.80 (24) |
| Boron | 18.0±5.04 (30) | | 2.2±3.24 (15) | | 1.9±2.70 (15) | | 11.5±3.18 (30) | | 4.2±3.40 (15) | | 28.9±6.39 (15) | | 15.8±5.02 (30) | | 31.7±5.82 (15) | | | 8.4±3.02 (15) |
| Calcium | 7652.9±600.16 (54) | | 1866.7±126.40 (27) | | 1126.7±273.74 (27) | | 3728.7±211.48 (54) | | 1511.5±135.69 (27) | | 6779.7±1040.79 (27) | | 9597.3±2523.86 (54) | | 11008.2±2351.84 (27) | | | 7007.6±1221.72 (27) |
| Cadmium | 24.3±28.99 (36) | | 18.7±24.08 (18) | | 17.3±18.62 (18) | | 61.5±25.40 (36) | | 52.5±29.30 (18) | | 5234.3±942.07 (18) | | 263.0±137.19 (36) | | 298.7±84.93 (18) | | | 939.0±300.40 (18) |
| Chromium | 8.8±6.90 (54) | | 12.6±10.39 (27) | | 9.8±6.10 (27) | | 35.4±6.26 (54) | | 17.2±9.57 (27) | | 5105.7±798.88 (27) | | 122.7±62.97 (54) | | 111.1±37.32 (27) | | | 525.4±173.61 (27) |
| Copper | 29.5±27.39 (54) | | 18.4±6.81 (27) | | 30.5±13.97 (27) | | 28.0±6.91 (54) | | 28.1±4.30 (27) | | 1375.6±219.61 (27) | | 48.5±22.92 (54) | | 59.5±15.97 (27) | | | 216.2±73.73 (27) |
| Iron | 152.0±42.26 (42) | | 42.6±6.89 (21) | | 211.1±44.38 (21) | | 274.9±92.15 (42) | | 114.0±20.37 (21) | | 8099.5±1234.60 (21) | | 331.2±180.1 (42) | | 333.9±96.55 (21) | | | 977.0±334.01 (21) |
| Magnesium | 1495.4±78.17 (54) | | 947.6±56.96 (27) | | 864.3±100.65 (27) | | 915.8±48.28 (54) | | 1093.1±124.65 (27) | | 1525.0±173.61 (27) | | 1097.5±159.23 (54) | | 1217.1±91.22 (27) | | | 437.0±63.11 (27) |
| Manganese | 41.8±18.10 (54) | | 18.5±11.83 (27) | | 74.8±13.51 (27) | | 35.4±10.08 (54) | | 24.9±7.53 (27) | | 1541.4±221.46 (27) | | 275.3±86.73 (54) | | 214.5±50.97 (27) | | | 609.5±145.57 (27) |
| Sodium | 1412.2±435.10 (24) | | 2899.4±237.52 (12) | | 4189.1±315.20 (12) | | 1123.2±442.61 (24) | | 1613.5±135.56 (12) | | 2039.9±260.78 (12) | | 1662.3±255.47 (24) | | 2385.1±87.18 (12) | | | 1089.9±71.99 (12) |
| Nickel | 6.1±5.15 (49) | | 10.2±23.75 | | 11.6±18.00 (27) | | 9.7±8.67 (49) | | 12.2±13.75 (27) | | 89.9±31.14 (27) | | 11.7±8.61 (49) | | 13.1±8.15 (27) | | | 40.8±32.57 (27) |
| Zinc | 131.5±54.56 (48) | | 407.8±55.44 (24) | | 135.0±68.40 (24) | | 129.8±69.81 (48) | | 374.4±129.54 (24) | | 2711.6±448.55 (24) | | 181.5±96.79 (48) | | 346.2±86.76 (24) | | | 505.3±252.88 (24) |
|  | | 7‒day hydraulic retention time (HRT) | | | | | | | | | | | | | | | | |
| Element | | *P. australis* in C3^d^ | | | | | | *P. australis* in T9^e^ | | | | | | *P. australis* in T10^f^ | | | | |
|  |  | Leaf | | Stem | | Rhizome | | Leaf | | Stem | | Rhizome | | Leaf | | Stem | Rhizome | |
| Aluminium | | 51.4±30.91 (48) | | 10.5±16.16 (24) | | 110.5±26.82 (24) | | 105.4±114.61 (51) | | 50.6±28.32 (24) | | 1450.2±124.73 (24) | | 104.4±147.50 (46) | | 18.9±21.73 (24) | 714.2±69.44 (24) | |
| Boron | | 14.5±5.96 (30) | | 3.1±3.54 (15) | | 1.6±2.37 (15) | | 18.8±4.49 (30) | | 10.5±3.25 (15) | | 15.9±4.27 (15) | | 10.6±4.10 (30) | | 2.7±2.93 (15) | 11.3±4.81 (15) | |
| Calcium | | 9196.9±690.76 (54) | | 2179.4±214.58 (27) | | 1709.0±141.14 (27) | | 6308.2±633.96 (54) | | 1648.2±172.03 (27) | | 4399.7±346.27 (27) | | 6039.2±504.45 (54) | | 1578.9±121.61 (27) | 13228.0±1847.21 (27) | |
| Cadmium | | 18.2±22.12 (36) | | 13.5±15.04 (18) | | 38.5±20.82 (18) | | 113.9±21.99 (36) | | 222.5±81.33 (18) | | 3548.8±381.14 (18) | | 95.7±128.98 (36) | | 40.2±18.11 (18) | 1209.1±201.80 (18) | |
| Chromium | | 7.1±4.16 (54) | | 7.7±3.51 (27) | | 39.1±3.71 (27) | | 55.3±8.16 (54) | | 54.2±9.51 (27) | | 2506.7±193.93 (27) | | 26.2±6.08 (54) | | 19.3±3.79 (27) | 538.4±72.01 (27) | |
| Copper | | 22.0±7.99 (54) | | 29.4±7.02 (27) | | 42.1±10.78 (27) | | 38.9±9.84 (54) | | 37.9±8.97 (27) | | 967.2±78.80 (27) | | 23.1±11.34 (54) | | 17.6±6.79 (27) | 261.8±32.08 (27) | |
| Iron | | 178.0±48.84 (42) | | 51.6±14.02 (21) | | 508.9±27.54 (21) | | 354.8±202.89 (42) | | 155.4±28.47 (21) | | 6352.9±471.40 (21) | | 312.4±199.93 (42) | | 76.9±12.72 (21) | 1572.4±215.68 (21) | |
| Magnesium | | 1589.4±84.58 (54) | | 1171.3±113.25 (27) | | 852.1±85.92 (27) | | 1771.4±166.71 (54) | | 1098.6±74.38 (27) | | 1096.9±56.29 (27) | | 1105.7±83.24 (54) | | 756.8±48.41 (27) | 1649.1±125.36 (27) | |
| Manganese | | 114.5±16.10 (54) | | 33.9±12.18 (27) | | 104.9±10.36 (27) | | 84.8±13.62 (54) | | 77.7±15.82 (27) | | 1348.4±86.37 (27) | | 54.8±11.59 (54) | | 30.7±12.12 (27) | 417.1±61.55 (27) | |
| Sodium | | 1087.8±368.86 (24) | | 1846.1±187.25 (12) | | 3130.6±376.17 (12) | | 1312.8±379.90 (24) | | 2508.7±127.92 (12) | | 1633.7±66.55 (12) | | 1011.1±382.83 (24) | | 1369.1±131.62 (12) | 1960.0±67.56 (12) | |
| Nickel | | 6.4±4.65 (49) | | 10.8±18.64 (27) | | 18.8±30.10 (27) | | 14.8±9.13 (49) | | 17.2±13.56 (27) | | 239.9±33.15 (27) | | 7.7±5.91 (49) | | 12.9±19.73 (27) | 56.0±21.82 (27) | |
| Zinc | | 159.3±50.96 (48) | | 460.4±83.21 (24) | | 129.2±55.66 (24) | | 209.9±73.11 (48) | | 350.1±65.48 (24) | | 1542.1±135.70 (24) | | 81.9±49.14 (48) | | 144.6±45.40 (24) | 498.5±160.44 (24) | |

* Data in table represented as; mean value ± Standard Deviation (number of tested samples)

^a^ C1, control wetland containing floating *P. australis* in tap water at 2‒day HRT

^b^ T1, HC‒SGW treatment systems 2-day with only floating *P. australis*

^c^ T2, HC‒SGW treatment systems 2-day with floating *P. australis* and ochre pellets

^d^ C3, control wetland containing floating *P. australis* in tap water at 7‒day HRT

^e^ T9, HC-SGW treatment systems 7-day with only floating *P. australis*

^f^ T10, HC-SGW treatment systems 7-day with floating *P. australis* and ochre pellets

**Online Resource 10** (Continued)

| b) LC‒SGW | | | | | | | | | | | | | |
| --- | --- | --- | --- | --- | --- | --- | --- | --- | --- | --- | --- | --- | --- |
|  | 2‒day hydraulic retention time (HRT)* | | | | | | | | | | | | |
| Element | *P. australis* in C1^a^ | | | *P. australis* in T5^b^ | | | | | *P. australis* in T6^c^ | | | | |
|  | Leaf | Stem | Rhizome | Leaf | Stem | | Rhizome | | Leaf | | Stem | | Rhizome |
| Aluminium | 25.8±22.51 (48) | 6.3±13.51 (24) | 25.9±26.60 (24) | 47.3±51.42 (47) | 9.7±12.53 (24) | | 2180.1±136.80 (24) | | 83.7±85.84 (45) | | 30.0±24.09 (24) | | 218.9±30.12 (24) |
| Boron | 18.0±5.04 (30) | 2.2±3.24 (15) | 1.9±2.70 (15) | 10.9±5.34 (30) | 3.4±3.82 (15) | | 9.2±4.96 (15) | | 10.7±4.17 (30) | | 2.5±2.48 (15) | | 2.1±2.30 (15) |
| Calcium | 7652.9±600.16 (54) | 1866.7±126.40 (27) | 1126.7±273.74 (27) | 5766.2±718.72 (54) | 1551.1±149.31 (27) | | 3856.4±661.27 (27) | | 7090.8±934.14 (54) | | 2795.8±275.16 (27) | | 10398.9±999.87 (27) |
| Cadmium | 24.3±28.99 (36) | 18.7±24.08 (18) | 17.3±18.62 (18) | 22.8±23.30 (36) | 21.0±20.06 (18) | | 1281.6±115.61 (18) | | 22.3±16.75 (36) | | 29.6±24.93 (18) | | 86.6±31.19 (18) |
| Chromium | 8.8±6.90 (54) | 12.6±10.39 (27) | 9.8±6.10 (27) | 15.1±10.34 (54) | 6.6±3.38 (27) | | 306.8±13.45 (27) | | 14.0±5.25 (54) | | 9.4±2.98 (27) | | 83.3±8.69 (27) |
| Copper | 29.5±27.39 (54) | 18.4±6.81 (27) | 30.5±13.97 (27) | 19.4±6.10 (54) | 23.7±6.95 (27) | | 734.9±31.63 (27) | | 16.0±5.87 (54) | | 13.6±5.35 (27) | | 48.9±7.81 (27) |
| Iron | 152.0±42.26 (42) | 42.6±6.89 (21) | 211.1±44.38 (21) | 166.2±61.57 (42) | 51.8±4.74 (21) | | 2850.5±612.90 (21) | | 194.1±82.58 (42) | | 105.9±18.67 (21) | | 697.7±64.40 (21) |
| Magnesium | 1495.4±78.17 (54) | 947.6±56.96 (27) | 864.3±100.65 (27) | 1229.9±143.27 (54) | 1183.2±79.25 (27) | | 972.7±55.30 (27) | | 670.0±225.44 (54) | | 351.0±17.77 (27) | | 566.3±39.11 (27) |
| Manganese | 41.8±18.10 (54) | 18.5±11.83 (27) | 74.8±13.51 (27) | 28.5±16.29 (54) | 19.2±12.09 (27) | | 963.0±150.64 (27) | | 53.3±18.51 (54) | | 32.3±13.59 (27) | | 113.2±11.04 (27) |
| Sodium | 1412.2±435.10 (24) | 2899.4±237.52 (12) | 4189.1±315.20 (12) | 880.3±286.12 (24) | 1016.9±33.66 (12) | | 1038.6±63.04 (12) | | 963.8±213.59 (24) | | 917.8±83.62 (12) | | 657.7±42.51 (12) |
| Nickel | 6.1±5.15 (49) | 10.2±23.75 (27) | 11.6±18.00 (27) | 5.3±3.03 (49) | 13.6±20.91 (27) | | 154.0±35.61 (27) | | 6.2±6.73 (49) | | 8.6±10.18 (27) | | 22.1±18.99 (27) |
| Zinc | 131.5±54.56 (48) | 407.8±55.44 (24) | 135.0±68.40 (24) | 196.3±64.09 (48) | 936.9±188.97 (24) | | 1032.6±130.47 (24) | | 87.0±57.8.0 (48) | | 81.7±35.62 (24) | | 122.7±49.37 (24) |
| Element | 7‒day hydraulic retention time (HRT) | | | | | | | | | | | | |
|  | *P. australis* in C3^d^ | | | *P. australis* in T13^e^ | | | | *P. australis* in T14^f^ | | | | | |
|  | Leaf | Stem | Rhizome | Leaf | Stem | Rhizome | | Leaf | | Stem | | Rhizome | |
| Aluminium | 51.4±30.91 (48) | 10.5±16.16 (24) | 110.5±26.82 (24) | 67.1±98.81 (44) | 14.2±18.83 (24) | 529.3±65.30 (24) | | 88.4±139.80 (48) | | 12.7±18.75 (24) | | 359.9±49.89 (24) | |
| Boron | 14.5±5.96 (30) | 3.1±3.54 (15) | 1.6±2.37 (15) | 15.7±5.77 (30) | 2.6±3.03 (15) | 5.7±4.92 (15) | | 9.9±3.83 (30) | | 2.5±2.44 (15) | | 2.8±2.48 (15) | |
| Calcium | 9196.9±690.76 (54) | 2179.4±214.58 (27) | 1709.0±141.14 (27) | 6876.1±574.30 (54) | 1354.8±78.18 (27) | 2302.2±258.14 (27) | | 6696.9±573.44 (54) | | 2119.7±377.36 (27) | | 21645.9±2540.44 (27) | |
| Cadmium | 18.2±22.12 (36) | 13.5±15.04 (18) | 38.5±20.82 (18) | 46.4±74.70 (36) | 28.1±19.00 (18) | 537.9±84.72 (18) | | 29.3±27.60 (36) | | 16.3±18.02 (18) | | 77.3±23.34 (18) | |
| Chromium | 7.1±4.16 (54) | 7.7±3.51 (27) | 39.1±3.71 (27) | 5.8±3.88 (54) | 9.5±4.01 (27) | 139.0±33.92 (27) | | 15.3±8.61 (54) | | 7.4±4.48 (27) | | 41.3±4.61 (27) | |
| Copper | 22.0±7.99 (54) | 29.4±7.02 (27) | 42.1±10.78 (27) | 20.7±8.19 (54) | 27.1±5.80 (27) | 229.5±25.71 (27) | | 16.0±8.16 (54) | | 13.1±8.21 (27) | | 44.0±12.74 (27) | |
| Iron | 178.0±48.84 (42) | 51.6±14.02 (21) | 508.9±27.54 (21) | 184.1±53.55 (42) | 60.5±6.66 (21) | 1194.0±157.61 (21) | | 206.9±56.80 (42) | | 58.8±8.75 (21) | | 594.6±25.88 (21) | |
| Magnesium | 1589.4±84.58 (54) | 1171.3±113.25 (27) | 852.1±85.92 (27) | 1334.3±97.65 (54) | 830.0±39.89 (27) | 991.7±59.51 (27) | | 592.9±47.24 (54) | | 388.3±72.28 (27) | | 411.3±19.46 (27) | |
| Manganese | 114.5±16.10 (54) | 33.9±12.18 (27) | 104.9±10.36 (27) | 46.4±14.54 (54) | 53.8±14.79 (27) | 675.8±70.68 (27) | | 51.0±12.79 (54) | | 32.4±9.57 (27) | | 108.1±9.95 (27) | |
| Sodium | 1087.8±368.86 (24) | 1846.1±187.25 (12) | 3130.6±376.17 (12) | 873.7±393.08 (24) | 701.0±86.37 (12) | 1627.8±101.80 (12) | | 952.8±400.13 (24) | | 1899.8±231.87 (12) | | 1119.3±61.48 (12) | |
| Nickel | 6.4±4.65 (49) | 10.8±18.64 (27) | 18.8±30.10 (27) | 6.2±5.11 (49) | 12.5±21.87 (27) | 47.3±16.37 (27) | | 7.3±7.09 (49) | | 7.6±9.66 (27) | | 23.5±14.91 (27) | |
| Zinc | 159.3±50.96 (48) | 460.4±83.21 (24) | 129.2±55.66 (24) | 102.2±66.30 (48) | 256.1±71.19 (24) | 446.3±91.84 (24) | | 78.2±49.81 (48) | | 117.8±89.31 (24) | | 136.6±59.78 (24) | |

* Data in table represented as; mean value ± Standard Deviation (number of tested samples)

^a^ C1, control wetland containing floating *P. australis* in tap water at 2‒day HRT

^b^ T5, LC-SGW treatment systems 2-day with only floating *P. australis*

^c^ T6, LC-SGW treatment systems 2-day with floating *P. australis* and ochre pellets

^d^ C3, control wetland containing floating *P. australis* in tap water at 7‒day HRT

^e^ T13, LC-SGW treatment systems 7-day with only floating *P. australis*

^f^ T14, LC-SGW treatment systems 7-day with floating *P. australis* and ochre pellets
